# High-Resolution LC-MS Simultaneous Quantification of Forty-Six Compounds from *Jatropha podagrica* Fruit Recommends Four Top Antioxidant Contributors as Q-Markers

**DOI:** 10.3390/molecules30030722

**Published:** 2025-02-05

**Authors:** Rongxin Cai, Xican Li, Honghong Liang, Shaoman Chen, Yuting Huang, Hanxiao Chai, Rongrong Lin, Guihua Jiang

**Affiliations:** 1 State Key Laboratory of Southwestern Chinese Medicine Resources, School of Pharmacy, Chengdu University of Traditional Chinese Medicine, Chengdu 611137, China; choi_sc@stu.cdutcm.edu.cn (R.C.); lianghonghong@stu.cdutcm.edu.cn (H.L.); 2 School of Chinese Herbal Medicine, Guangzhou University of Chinese Medicine, Guangzhou 510006, China; 20221110152@stu.gzucm.edu.cn (S.C.); 20231110153@stu.gzucm.edu.cn (Y.H.); 20221110228@stu.gzucm.edu.cn (H.C.); 20221110155@stu.gzucm.edu.cn (R.L.)

**Keywords:** corilagin, gallic acid, isomers distinction, *Jatropha podagrica*, quality control, quality assessment, UPLC-Q-Orbitrap MS/MS

## Abstract

There has been no chemical analysis of the fruit of medicinal plant *Jatropha podagrica* until now. The current study aimed to qualitatively and quantitatively analyze the *J. podagrica* fruit using a high-resolution LC-MS strategy, i.e., library-comparison ultra-high-performance liquid chromatography-Quadrupole-Orbitrap-tandem mass spectrometry. The strategy putatively identified 46 compounds from fresh fruit. During the putative identification, 10 isomers (e.g., (vitexin vs. isovitexin) were completely distinguished from each other. Thereafter, all 46 compounds were simultaneously quantified using authentic standard comparison method. Finally, they were also subjected to the 2,2′-azino bis (3-ethylbenzothiazolin-6-sulfonic acid radical (ABTS^+•^)-scavenging assay to characterize their relative antioxidant capacities. Their antioxidant capacities were thus multiplied by chemical contents to calculate their antioxidant contribution values, respectively. Corilagin, gallic acid, ellagic acid, and phillygenin exhibited the highest antioxidant contribution percentages and thereby were suggested as the four top antioxidant contributors. The four are recommended to build up a quality-markers (Q-markers) system of *J. podagrica* fruits. All these findings can help to develop *J. podagrica* fruits as a potential resource of natural medicine.

## 1. Introduction

**Figure 1:**
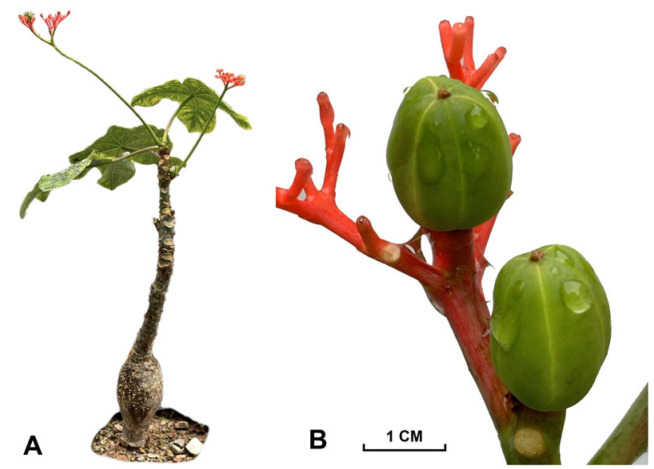
Photos of *Jatropha podagrica*. (**A**) the plant morphology; (**B**) the fruits.

*Jatropha podagrica* Hook (Figure 1) is a shrub widely distributed in Africa, Asia, and Latin America [1,2]. Due to its distinctive appearance, *J. podagrica* is cultivated as an ornamental plant. Additionally, it is also used as medicinal and edible plant species [3]. As a medicinal species, *J. podagrica* has been employed to treat skin infections [1], sexually transmitted diseases (e.g., gonorrhea) [1,4], and jaundice and fever [5]. Its seed oil is used as a crucial raw material for biodiesel production and industrial products [6,7]. Overall, *J. podagrica* has various traditional functions around the world.

Modern scientific research has mainly focused on its seeds [8], leaves [9], and bark [10]. For instance, Yin and co-workers analyzed 11 volatile compounds of *J. podagrica* seeds, including palmitic acid and linoleic acid [8]. Ghali’s team investigated the antioxidant, anti-proliferative, and cytoprotective potentials of the seeds and leaves of *J. podagrica*, suggesting that the seeds possessed higher total phenolic and flavonoid contents and exhibited stronger antioxidant capacities than the leaves [5].

Phenolics (especially flavonoids) occurring in fruits, however, have been found to be able to act as bioactive compounds and play a beneficial role in human health by previous studies. For example, *Terminalia chebula* fruits have been shown to contain high level of phenolic compounds and exhibit strong free-radical scavenging activity [11,12]. Similarly, *Morinda citrifolia* (noni) fruits were observed to possess potent immunomodulatory and antioxidant effects for the occurrence of bioactive phenolics [13]. These examples suggest the possibility that *J. podagrica* fruits enriched by phenolics may also be a library of bioactive compounds and may benefit human health as well.

As a carrier for the seed, *J. podagrica* fruits have been indeed reported to enrich various bioactive compounds. These fruits have demonstrated potential biological activity and medicinal value [14,15]. However, there is no systematic analysis of *J. podagrica* fruit. Considering that chemistry is the prerequisite for biological activity and medicinal value, it is necessary to offer a deep chemical insight into *J. podagrica* fruits.

The current study thus introduces a high-resolution LC-MS method, namely library-comparison UHPLC-Q-Exactive-Orbitrap-MS/MS analysis [16]. This method can putatively identify various compounds through MS/MS elucidation, mass spectral comparison, and retention time (R.T.) alignment with authentic standards [17]. Particularly, it is good at isomer distinction and simultaneous quantification of multiple compounds [18,19]. It is expected to provide an in-depth chemical insight into the fruit, which will further uncover the material basis of its traditional applications and promote its development as a bio-resource around the world.

## 2. Results and Discussion

In the current study, the extract of *J. podagrica* fruits was prepared and subject to a high-resolution LC-MS analysis, that is, UHPLC-Q-Exactive-Orbitrap MS/MS. In terms of effectiveness, this analytical approach significantly outperformed the conventional HPLC method, which could detect only a limited number of bioactive compounds [20]. Regarding reliability, the present high-resolution LC-MS identification was superior to previous identifications [21,22,23,24]. One principal reason is that earlier analyses lacked authentic standards for comparison and thus had to rely on documented data. For instance, a 2022 study attempted to match electrospray ionization tandem (ESI) MS data with Orbitrap MS data, but the two experimental setups were not directly comparable, undermining the validity of those identifications. Conventionally, these identifications are described as “tentative” rather than “putative”. Such tentative identification could not distinguish the isomer [23,24,25,26] and accordingly were incompetent for the analysis of *J. podagrica* fruits.

**Figure 2:**
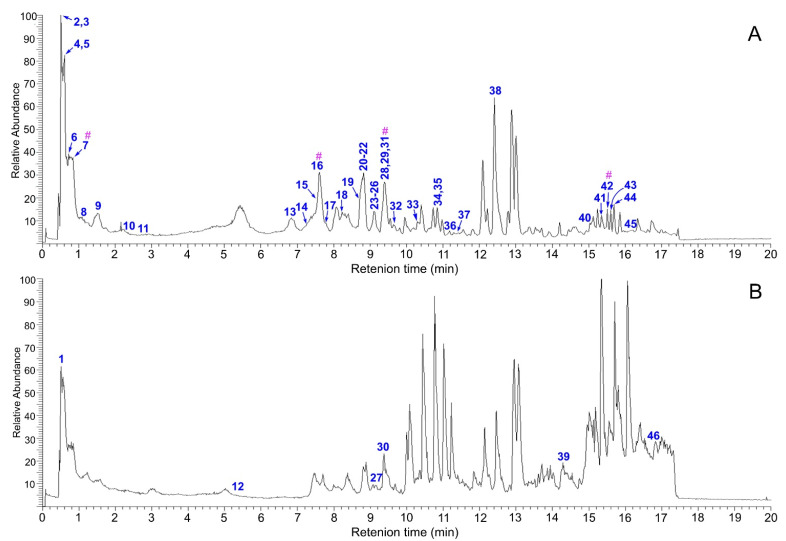
The total ion current (TIC) diagrams of *J. podagrica* fruits obtained by UHPLC-Q-Exactive-Orbitrap MS/MS analysis under two ionization modes: (**A**) negative ion mode and (**B**) positive ion mode. The positive ion mode was supplement of negative ion mode. **#**, potential Q-marker.

In the study, the cutting-edge UHPLC-Q-Exactive-Orbitrap MS/MS technology was combined with a TCM-specific library [27] to develop a novel library-comparison UHPLC-Q-Exactive-Orbitrap MS/MS identification method. Using the method, *J. podagrica* fruits were found to contain 40 compounds under negative ion mode. Additionally, six compounds were identified under positive ion mode. Thus, a total of 46 compounds were identified using the library-comparison UHPLC-Q-Exactive-Orbitrap MS/MS method (Table 1). The TIC diagrams are shown in Figure 2.

**Figure 3:**
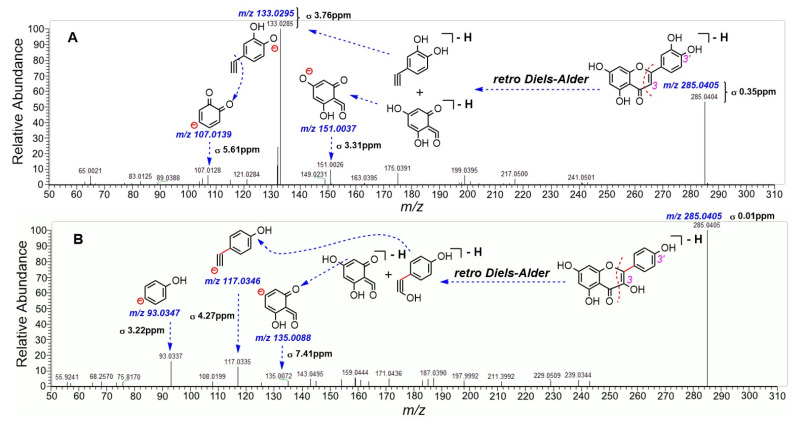
Differentiation of two isomers (**35** luteolin and **36** kaempferol). (**A**) MS/MS spectra of **35**; (**B**) MS/MS spectra of **36**. Chromatography and mass-spectrometer conditions are provided in Section 3.4. Blue italic digits denote the calculated *m/z* values of MS/MS fragments. The δ value represents the error between theoretical and observed *m/z* values, expressed in ppm. Due to space limitations, MS/MS fragments are not shown in their deprotonated forms.

During the identification process, we successfully distinguished two functional group isomers, i.e., luteolin (**35**) and kaempferol (**36**). As illustrated in Figure 3, the *m/z* value of compound (**35**) is 285.0404, while that of compound (**36**) is 285.0405; meanwhile, both isomers (**35** and **36**) exhibit different fragmentation patterns. Compound (**35**) undergoes classical *retro* Diels–Alder cleavage, producing three ion peaks (*m/z* 151, 133, and 107). In contrast, compound (**36**) undergoes the same cleavage to yield two ion peaks (*m/z* 135 and 117). Thus, in line with these differences and our previous principles [17], two functional group isomers were successfully distinguished in the study. On the other hand, during the process of fragmentation elucidations of the two isomers, we observed very low δ values between the theoretical and observed *m/z* values (0.01 ppm~7.41 ppm, Figure 3). This implies that our elucidations were adequately accurate in the study.

**Figure 4:**
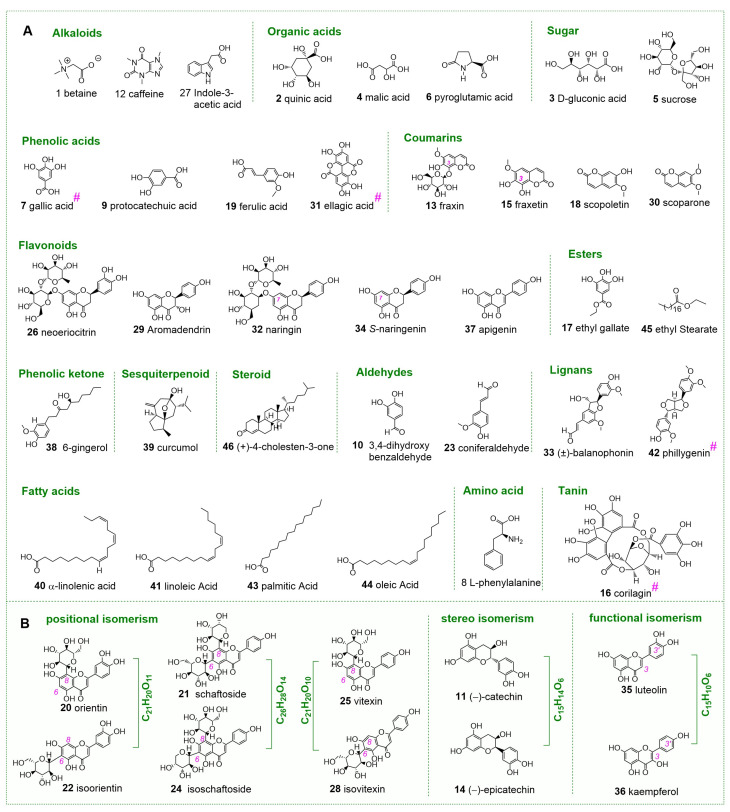
The structures and configurations of 46 identified compounds from *Jatropha podagrica* fruits by library-comparison UHPLC-Q-Orbitrap MS/MS method. (**A**) Non-isomeric compounds; (**B**) Isomeric compounds. #, potential Q-marker.

In addition, the current study successfully distinguished three pairs of positional isomers (vitexin **25** vs. isovitexin **28**, orientin **20** vs. isoorientin **22**, and schaftoside **21** vs. isoschaftoside **24**, Figure 4), and one pair of diastereoisomers ((−)-catechin **11** vs. (−)-epicatechin **14**, Figure 4). Besides isomers distinction, the study also comprehensively elucidated the MS/MS spectra of all identified compounds (Supplementary Data S2). The isomers distinction, along with the aforementioned low δ values in elucidation, validated the accuracy of compound identification.

This accurate identification also discovered 35 “unexcavated” compounds, including corilagin (**16**), ellagic acid (**31**), and phillygenin (**42**, Table 1). To our knowledge, these compounds have not been documented in previous studies concerning the *J. podagrica* plant [10,80,81].

After the qualitative analysis, a quantitative analysis was performed using the library-comparison UHPLC-Q-Exactive-Orbitrap-MS method. As shown in Table 1 and Supplementary Data S3, all identified compounds (**1**–**46**) had contents ranging from 0.03 ± 0.00 to 325.32 ± 11.46 μg/g. Five compounds showed the highest contents, including sucrose (**5**), corilagin (**16**), isoorientin (**22**), gallic acid (**7**), and ellagic acid (**31**). All these qualitative and quantitative analyses indicated that the fruit may differ from the seeds and other parts of *J. podagrica*, from the angle of chemistry [82,83].

On the other hand, these 46 compounds (**1**–**46**) have been documented to exhibit various bioactive (or nutritional) functions, such as antioxidation (Table 1, rightmost column). The study therefore further characterized their relative antioxidant capacities using the ABTS^+•^ scavenging assay under the same conditions. The results are expressed as “antioxidant percentages” in Table 1. It was found that the antioxidant percentages of the 46 compounds varied at the range of 0.00~100.00%. For instance, the antioxidant percentage of gallic acid (**7**) was 100.00%, and those of corilagin (**16**) and ellagic acid (**31**) were 59.12% and 75.03%, respectively.

However, the antioxidant percentage of individual compound alone cannot effectively assess its role within the entire fruit. Therefore, this study defined an equation (Equation (1)) to calculate the antioxidant contribution value of one antioxidant.

(1) Antioxidant contribution value=content×antioxidant percentage

**Figure 5:**
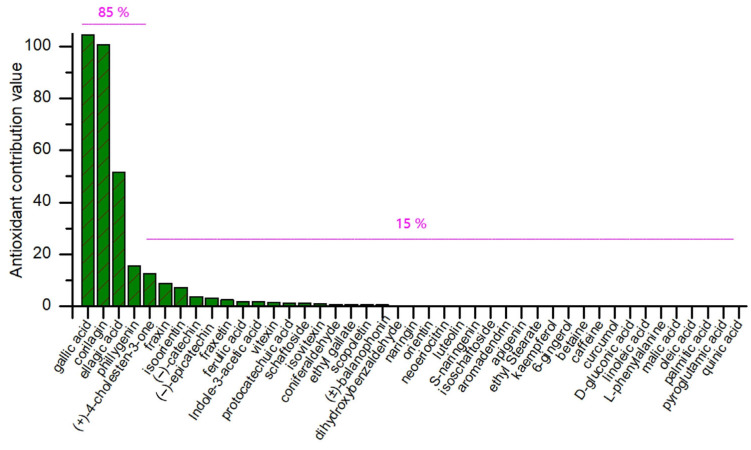
The antioxidant contribution distribution of 46 compounds (**1**–**46**) in *J. podagrica* fruits.

Based on the calculation, the antioxidant contribution values of the 46 compounds (**1**–**46**) are ranked in Figure 5, where four compounds (**7**, **16**, **31**, and **42**) are found to display much higher antioxidant contribution values than the other 42 compounds. These four (**7**, **16, 31**, and **42**) accounted for 85% of the total antioxidant contribution and thus were regarded as the top antioxidant contributors towards the whole *J. podagrica* fruit.

Antioxidation is well known to play a critical role in various pharmacological effects. In fact, the top four contributors (**7**, **16**, **31**, and **42**) indeed possess antioxidant-related activities, including anti-inflammatory, immunosuppressive, antitumor applications, and neuroprotection [35,75,84,85,86]. Thus, the selection of these Q-markers met the “pharmacological relevance” principle of Liu Changxiao [87].

From the perspective of analytical chemistry, the four Q-marker candidates (**7**, **16**, **31**, and **42**) showed high testability as well, because all of them exhibited strong peak signals in the TIC diagram (Figure 2). Of course, the peaks of compounds **7** and **31** were not so independent from the neighbors. However, under the selected ion monitoring (SIM) mode, their peaks were highly independent and robust (Supplementary Data S2, Figures S7C and S31C). Thus, they could be easily detected under the SIM mode using LC-MS technologies, such as UHPLC-Q-Orbitrap-MS/MS analysis. In short, the selection of four Q-marker candidates (**7**, **16**, **31**, and **42**) followed the so-called testability principle [87].

Of these Q-marker candidates (**7**, **16**, **31**, and **42**), the former three could be mutually transformed via bio-genetic approaches. For example, ellagitannin corilagin (**16**) could be hydrolyzed to produce ellagic acid (**31**); ellagic acid (**31**), however, could be further degraded to result in gallic acid (**7**) [84]. Therefore, the three (**7**, **16**, and **31**) usually co-exist in the same plant, e.g., *Terminaliae belliricae* [88], implying that they lack adequate specificity to characterize *J. podagrica* fruits. However, when lignan phillygenin (**42**) was integrated with the three, the combined Q-markers system, i.e., **7**, **16**, **31**, and **42**, was able to specifically characterize *J. podagrica* fruits. To our knowledge, no plant has been reported to simultaneously comprise these four Q-markers (**7**, **16**, **31**, and **42**). This indicates that the Q-marker (**7**, **16**, **31**, and **42**) system has adequate specificity for *J. podagrica* fruits.

**Figure 6:**
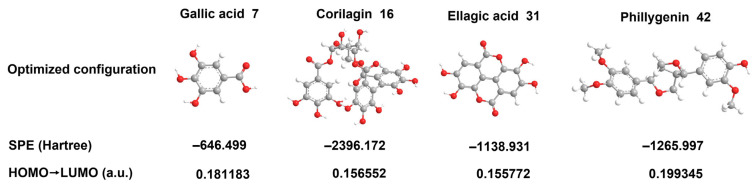
The optimized conformation, single point energy (SPE) values, and the energy gaps from the lowest unoccupied molecular orbital to the highest occupied molecular orbital (LUMO → HOMO) for gallic acid (**7**), corilagin (**16**), ellagic acid (**31**), and phillygenin (**42**). The quantum chemical calculation was conducted using RB3LYP-D3(BJ) method and at 6-31G(d,p) basis set without solvation.

Besides pharmacological relevance, testability, and specificity principles, the study also investigated the so-called “stability”, a principle proposed by our team [89], by means of quantum chemical calculations. As shown in Figure 6, the single point energy (SPE) value of gallic acid (**7**) was calculated to be −646.699 Hartree. However, it was stable enough to withstand heating treatment [90], meaning that it had adequate thermodynamic stability. The other three compounds (**16**, **31** and **42**) showed lower SPE values compared with gallic acid (**7**). Thus, the latter three exhibited higher stability than gallic acid, from a thermodynamic perspective. These could be responsible for the fact that the four Q-markers (**7**, **16**, **31**, and **42**) could be successfully detected by means of serum pharmacochemistry analysis [88,91]. Serum is the destination of these natural compounds, after the medicinal plant has undergone a series of treatments, including harvesting, drying, transport, storage, extraction, and gavage administration. The successful detection in serum apparently suggests a good traceability of the four Q-markers (**7**, **16**, **31**, and **42**). Now it is clear that the four Q-markers (**7**, **16**, **31**, and **42**) follow Liu’s traceability principle. This, however, can be further attributed to their thermodynamic stability. As seen in Figure 6, the LUMO → HOMO energy gaps of four Q-markers decreased in the following order: **42** > **7** > **16** > **31**. Ellagic acid (**31**), however, was selected as the Q-marker of *Granati Pericarpium* (Shiliupi) by Pharmacopoeia Commission [92], suggesting that ellagic acid (**31**) has enough thermodynamic stability. In fact, even in charred *Granati Pericarpium*, ellagic acid (**31**) could be successfully detected out [93]. In other words, heating and even charring could not easily facilitate ellagic acid to occur a chemical reaction. Obviously, the high reaction inertness was ultimately attributed to the high LUMO → HOMO energy gap of ellagic acid (**31**). The other three Q-markers (**42**, **7**, and **16**) with higher LUMO → HOMO energy gaps were assumed to possess higher thermodynamic stability than Q-marker (**31**). Now it is clear that all four Q-markers (**7**, **16**, **31**, and **42**) adhered to our stability principle as well.

Besides the stability principle, our team also proposed an “artificially unobtainability principle” to prevent the possible artificial addition of Q-marker compounds into plant materials [16]. Of the four Q-marker candidates, corilagin (**16**) and phillygenin (**42**) could not be easily obtained via artificial synthesis, because corilagin (**16**) contained a 12-membered ring, while phillygenin (**42**) comprised four chiral atoms (Figure 4). The two cannot become inexpensive industrial chemicals due to the lack of a mature synthesis route to date. This can thus effectively prevent the illegal addition similar to the Sanlu Melamine Incident in China (2008), because the essence of the Sanlu Melamine Incident was an illegal addition using inexpensive industrial chemicals. In summary, the four Q-marker candidates (**7**, **16**, **31**, and **42**) comply with the principles proposed by Liu’s and our teams. Therefore, the study recommends them as Q-markers of *J. podagrica* fruits.

It should be emphasized that (1) the Q-marker screening was only based on one *J. podagrica* species locating in Guangzhou (China). The chemical contents of *J. podagrica* species located in other countries may be different from it. Nevertheless, the four Q-markers (**7**, **16**, **31**, and **42**) are assumed to be detectable, owing to their high contents. Thus, the four Q-markers (**7**, **16**, **31**, and **42**) are also applicable for quality assessment of the plants in other countries or regions. (2) Although some nutrients (e.g., linoleic acid **41** and palmitic acid **43**) have also been mentioned in the previous study [8] and found in one study (Table 1), the current study does not plan to discuss them for the irrelevance to antioxidation.

## 3. Materials and Methods

### 3.1. Plant Materials, Chemicals, and Authentic Standards

Fruits of *J. podagrica* were collected on 3 January 2023 from the Guangdong Provincial Traditional Chinese Medicine Resource Conservation Garden, the Fourth National Survey of Chinese Materia Medica Resources. The species was identified by Prof. Xican Li. Methanol and water employed in this study were of mass spectrometry-grade purity. Detailed information on all authentic standards, including molecular formula, molecular weight, CAS number, purity, and supplier is provided in Supplementary Data S1.

### 3.2. Preparation of Lyophilized Aqueous Extract from J. podagrica Fruits and Sample Solution

**Figure 7:**
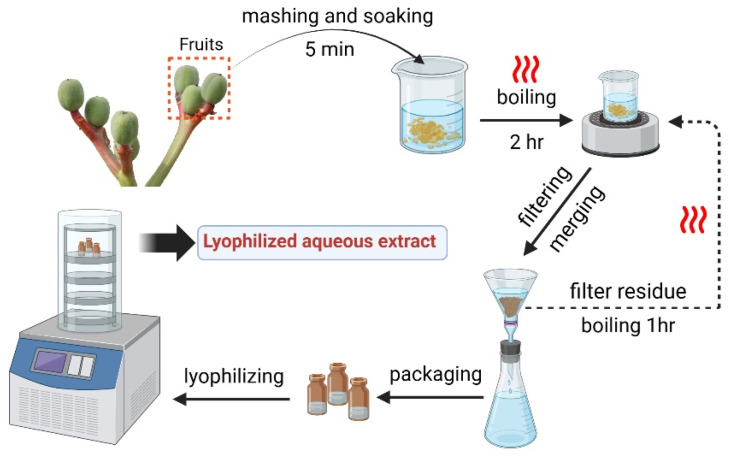
Flow chart of preparation of lyophilized aqueous extract from *Jatropha podagrica* fruits.

To minimize solvent-related effects [94], fresh fruits of *J. podagrica* were fragmented and subsequently extracted using distilled water. The extract was subjected to lyophilization using a freeze dryer (FDU-1200, Eyela Co., Ltd., Shanghai, China). The lyophilized powder was subsequently re-dissolved in methanol with ultrasonic assistance and filtered through a 0.22 μm nylon membrane to prepare a sample solution at a concentration of 30 mg/mL (Figure 7) [95].

### 3.3. Preparation of Authentic Standard Solutions

Each of the 46 authentic standards was individually dissolved in methanol using ultrasonic treatment. These standard solutions were filtered through 0.22 μm membranes to yield standard solutions at approximately 0.1 μg/mL. Both the sample solution and the standard solutions were stored at 4 °C until further analysis.

### 3.4. Chromatographic and Mass Spectrometric Conditions for UHPLC-Q-Orbitrap-MS Determination

Chromatographic separation was carried out using on an Accucore RP-MS LC C_18_ column (100 mm × 2.1 mm, 2.6 μm, Thermo Fisher, Waltham, MA, USA) incorporated into a UHPLC system (Thermo Fisher Scientific, Waltham, MA, USA). The chromatographic conditions were obtained from a previous study with minor modifications [89]. In the negative ion mode, mobile phase A consisted of 0.1% formic acid (HCOOH) and mobile phase B was 100% methanol (CH_3_OH). In the positive ion mode, mobile phase A was a mixture of 5 mmol/L ammonium acetate and 0.1% formic acid, while mobile phase B remained at 100% methanol. The mobile phases of the negative ion mode and the positive ion mode were run at the following gradient elution program: 0.0–5.0 min, 10% B; 5.0–14.5 min, linear increase from 10% to 100% B; 14.5–16.0 min, 100% B; 16.0–20.0 min, re-equilibration to 10% B. The column temperature was maintained at 40 °C, and the injection volume was 3 μL.

The UHPLC system was coupled to a high-resolution Q-Exactive Orbitrap mass spectrometer (Thermo Fisher Scientific, Waltham, MA, USA). Operating conditions included an auxiliary gas flow rate of 10 units, a sheath gas flow rate of 40 units, and a sweep gas flow rate of 0 units. The spray voltage was set to 4.5 kV. Both the auxiliary gas heater and capillary temperatures were held at 450 °C. Full MS and dd-MS^2^ scans were recorded at resolutions of 70,000 and 17,500, respectively, with an AGC target of 2 × 10⁵. Nitrogen (N_2_) was served as the spray stabilization gas and damping gas in the C-trap. Stepped normalized collision energies (NCEs) were set to 20, 50, and 100 V [96].

### 3.5. UHPLC-Q-Orbitrap-MS Analysis: Data Acquisition and Putative Identification

The acquisition of parameters (e.g., retention times, molecular ion peaks, and MS/MS spectra) was performed using the Xcalibur 4.1 software and TraceFinder 4.1 General Quan (Thermo Fisher Scientific Inc., Waltham, MA, USA) [96]. Analytical settings were established as follows: mass range from 100 to 1200 Da, mass tolerance of 10 ppm, signal-to-noise (S/N) ratio threshold of 5, retention time adjustment window of 30 min, and isotopic pattern matching threshold of 90%. Based on these data, compounds present in the fruits of *J. podagrica* were preliminarily identified using structural information. Manual fragmentation analysis of the MS spectra was then conducted to validate and complete the putative identification of these compounds.

### 3.6. Quantum Chemical Calculations

Quantum chemical calculations were performed on four compounds (gallic acid, corilagin, ellagic acid, and phillygenin) using density functional theory. These calculations included conformational optimization, determination of the lowest energies (i.e., single point energy) employing the RB3LYP/6–31G(d,p) basis set [97]. The most stable conformations were confirmed by verifying the absence of imaginary frequencies using Gaussian 16 software on a Linux (Ubuntu) platform. The optimized conformations were visualized and exported using GaussView 6.1.1 (Gaussian, Inc., Wallingford, CT, USA) [16].

### 3.7. Quantitative Analysis

Simultaneous qualitative and quantitative analyses of 46 compounds from *J. podagrica* fruits were conducted using UHPLC-Q-Orbitrap-MS/MS. Calibration curves were established by correlating the concentrations of 46 authentic standards with their respective peak areas. The peak areas of the identified compounds were measured using TraceFinder 4.1 General Quan (Thermo Fisher Scientific Inc., Waltham, MA, USA) and quantified against the calibration curves. The analysis parameters were set as follows: threshold override of 10,000; mass tolerance of 5 ppm; S/N threshold of 5; retention time window override of 100 min; fragment time of 1 min; and isotopic pattern fit threshold of 80%.

### 3.8. 2,2′-Azino bis(3-ethylbenzothiazolin-6-sulfonic acid radical (ABTS^+•^)-Scavenging Antioxidant Evaluation

The ABTS^+•^ scavenging activity was assessed following the procedure described by Xie et al. [98]. ABTS^+•^ radicals were produced by combining 0.2 mL of 7.4 mmol/L ABTS diammonium salt with 0.2 mL of 2.6 mmol/L potassium persulfate, and the mixture was incubated in the dark at room temperature for 12 h to ensure complete radical formation. The resulting solution was then diluted 1:20 with distilled water until its absorbance at 734 nm reached 0.83 ± 0.05, as measured using a microplate reader (Multiskan FC, Thermo Scientific, Shanghai, China). For the assay, 3 μL of each test sample (0.5 mg/mL) was mixed with 100 μL of distilled water and 80 μL of the ABTS^+•^ solution. After a 3 min reaction, the absorbance at 734 nm was recorded, using distilled water as the blank.

### 3.9. Statistical Analysis

All quantitative analyses were performed in triplicate, and results are presented as the mean ± standard deviation (SD) from three independent measurements. Correlation coefficients (R values) were calculated based on linear regression analysis using Origin 6.0 software (OriginLab Corporation, Northampton, MA, USA).

## 4. Conclusions

Through a library-comparison UHPLC-Q-Exactive-Orbitrap MS/MS analysis, *J. podagrica* fruits were found to enrich 46 bioactive (or nutritional) compounds, including 35 “unexcavated” ones and 10 isomeric ones. Forty-six compounds showed different chemical contents, relative antioxidant capacities, and antioxidant contribution values. Corilagin, gallic acid, ellagic acid, and phillygenin were the top four antioxidant contributors and accordingly are recommended to construct a Q-marker system for *J. podagrica* fruits. These findings promise the development potential of *J. podagrica* fruits, while the recommendation can help to establish a feasible quality assessment method.

## Figures and Tables

**Table 1:** The main experimental results of 46 identified compounds (**1**–**46**).

IDs	RT Min	Name	Molecular Ion	Observed *m/z* Value	Theoretical *m/z* Value	Error (δ ppm)	Characteristic MS/MS Fragments	Content (µg/g)	Antioxidant Percentage %	Documental Evidence	Bioactivity
**1**	0.51	betaine *	C_5_H_11_NO_2_	118.0865	118.0862	1.18	118.0865, 56.0503, 58.0660, 58.0738	0.20 ± 0.01	0.00 ± 0.71	Unexcavated	Antioxidation [28]
**2**	0.53	quinic acid	C_7_H_12_O_6_	191.0554	191.0561	3.13	191.0554, 127.0390, 85.0283, 59.0126	18.29 ± 0.30	0.00 ± 0.90	Unexcavated	Anti-inflammation [29]
**3**	0.53	D-gluconic acid	C_6_H_12_O_7_	195.0501	195.0510	3.15	195.0501, 129.0182, 99.0075, 75.0075	62.20 ± 0.28	0.00 ± 0.52	Unexcavated	Antidiabetic activity [30]
**4**	0.56	malic acid	C_4_H_6_O_5_	133.0133	133.0142	6.98	133.0133, 115.0026, 89.0229, 71.0126	54.03 ± 1.23	0.00 ± 0.34	Unexcavated	Antioxidation [31], Cardioprotection [32]
**5**	0.59	sucrose	C_12_H_22_O_11_	341.1095	341.1089	1.64	34.1096, 179.0549, 113.0233, 89.0233, 59.0127	325.32 ± 11.46	0.00 ± 0.59	Unexcavated	Analgesia [33]
**6**	0.67	pyroglutamic acid	C_5_H_7_NO_3_	128.0343	128.0353	7.35	82.0286, 128.0343, 129.0380	29.50 ± 9.17	0.00 ± 0.52	Unexcavated	Neuroprotection [34]
**7**	0.75	gallic acid #	C_7_H_6_O_5_	169.0131	169.0142	4.50	69.0334, 79.0177, 97.0284, 125.0234, 126.0267, 169.0134	104.57 ± 0.14	100.00 ± 0.34		Antioxidation, Anti-inflammation [35]
**8**	1.17	L-phenylalanine	C_9_H_11_NO_2_	164.0710	164.0717	4.73	164.0710, 147.0443, 164.0710	20.48 ± 1.37	0.00 ± 0.39	Unexcavated	Antibacteriality [36]
**9**	1.53	protocatechuic acid	C_7_H_6_O_4_	153.0185	153.0193	5.16	109.0284, 108.0206, 91.0178, 81.0335	1.32 ± 0.04	93.33 ± 0.98	Unexcavated	Immunomodulation [37]
**10**	2.32	3,4-dihydroxybenzaldehyde	C_7_H_6_O_3_	137.0236	137.0244	6.16	137.0235, 138.0269, 136.0156, 108.0206, 81.0334	0.16 ± 0.00	99.89 ± 0.52	Unexcavated	Antioxidation [38]
**11**	2.84	(−)-catechin	C_15_H_14_O_6_	289.0721	289.0718	1.22	289.0715, 203.0707, 109.0283, 57.0332	5.06 ± 0.06	84.63 ± 4.36	Unexcavated	Antioxidation [39]
**12**	5.36	caffeine *	C_8_H_10_N_4_O_2_	195.0871	193.0876	2.99	195.0870, 138.0657, 123.0428, 110.0716, 83.0609, 69.0455, 56.0503	0.12 ± 0.13	0.00 ± 0.20	Unexcavated	Antioxidation [40]
**13**	6.82	fraxin	C_16_H_18_O_10_	369.0826	369.0827	0.30	369.0823, 207.0294, 192.0058, 135.0078, 107.0128	33.35 ± 1.06	26.10 ± 2.37		Anti-inflammation [41]
**14**	7.26	(−)-epicatechin	C_15_H_14_O_6_	289.0716	289.0718	0.68	289.0716, 203.0705, 123.0441, 109.0284, 83.0124	3.30 ± 0.01	93.79 ± 0.20	Unexcavated	Anti-inflammation [42]
**15**	7.55	fraxetin	C_10_H_8_O_5_	207.0294	207.0299	2.32	207.0292, 192.0057, 164.0105, 108.0206	2.79 ± 0.04	84.63 ± 3.91		Antitumor activity [43]
**16**	7.58	corilagin #	C_27_H_22_O_18_	633.0733	633.0733	0.06	633.0724, 3000.9985, 201.0184, 145.0284	189.21 ± 5.61	59.12 ± 0.29	Unexcavated	Anti-inflammation [44]
**17**	7.79	ethyl gallate	C_9_H_10_O_5_	197.0451	197.0456	2.41	197.0446, 169.0130, 124.0154, 78.0098,	0.64 ± 0.01	94.69 ± 6.07		Anti-inflammation [45], Antidiabetic activity [46]
**18**	8.31	scopoletin	C_10_H_8_O_4_	191.0344	191.035	3.15	191.0339, 176.0108, 148.0158, 104.0255,	1.36 ± 0.03	42.60 ± 2.26	Unexcavated	Anti-inflammation [47]
**19**	8.62	ferulic acid	C_10_H_10_O_4_	193.0501	193.0506	2.93	193.0496, 178.0624, 149.0596, 134.0364	1.78 ± 0.12	97.85 ± 1.09	Unexcavated	Anti-inflammation [48]
**20**	8.78	orientin	C_21_H_20_O_11_	447.0934	447.0933	0.30	447.0921, 327.0507, 253.0506, 133.0284	0.48 ± 0.01	23.84 ± 0.85	Unexcavated	Anti-inflammation [49], Antineoplastic activity [50]
**21**	8.84	schaftoside	C_26_H_28_O_14_	563.1409	563.1406	0.42	563.1395, 353.0660, 97.0763, 117.0334	23.87 ± 0.23	4.41 ± 0.34	Unexcavated	Immunomodulation [51]
**22**	8.86	isoorientin	C_21_H_20_O_11_	447.0934	447.0933	0.30	447.0934, 357.0606, 299.0565, 133.0285	123.05 ± 1.42	5.76 ± 1.22	Unexcavated	Anti-inflammation [52]
**23**	8.99	coniferaldehyde	C_10_H_10_O_3_	177.0551	177.0557	3.42	178.0504, 162.0315, 134.0362, 109.0284	1.31 ± 0.04	52.88 ± 2.44	Unexcavated	Neuroprotection [53]
**24**	9.12	Isoschaftoside	C_26_H_28_O_14_	563.1432	563.1406	4.65	563.1399, 363.0661, 297.0763, 117.033	4.11 ± 0.12	2.03 ± 0.34	Unexcavated	Anti-inflammation [54]
**25**	9.15	vitexin	C_21_H_20_O_10_	431.0989	431.0984	1.25	431.0981, 311.0561, 283.0612, 117.0335	9.03 ± 0.17	14.69 ± 1.41		Antioxidation, Neuroprotection [55]
**26**	9.15	neoeriocitrin	C_27_H_32_O_15_	595.1675	595.1668	1.18	595.1663, 459.119, 135.0442, 65.0021	0.88 ± 0.02	12.77 ± 2.26	Unexcavated	Neuroprotection [56]
**27**	9.2	indole-3-acetic acid *	C_10_H_9_NO_2_	176.0700	176.0706	3.42	176.0711, 130.0655, 103.0548, 77.0394	3.20 ± 0.13	50.62 ± 1.28	Unexcavated	Anti-inflammation [57]
**28**	9.32	isovitexin	C_21_H_20_O_10_	431.0989	431.0984	1.18	431.0976, 311.0559, 283.0610, 117.0335	6.21 ± 0.03	14.69 ± 1.87		Antineoplastic activity [58]
**29**	9.36	aromadendrin	C_15_H_12_O_6_	287.0565	287.0561	1.21	287.0560, 259.0611, 177.0551, 125.0233	0.40 ± 0.01	18.76 ± 1.19	Unexcavated	Immunomodulation [59], Cardioprotection [60]
**30**	9.37	scoparone *	C_11_H_10_O_4_	207.0648	207.0652	2.01	207.0651, 151.0757, 107.0491, 91.0547	0.19 ± 0.00	0.00 ± 0.20		Antihepatitis [61]
**31**	9.45	ellagic acid #	C_14_H_6_O_8_	300.9992	300.9990	0.72	300.9985, 229.0131, 145.0285, 117.0334	68.87 ± 1.07	75.03 ± 3.97	Unexcavated	Antitumor activity [62]
**32**	9.64	naringin	C_27_H_32_O_14_	579.1724	579.1719	0.85	579.1708, 271.0610, 151.0026, 119.0490	1.84 ± 0.04	8.25 ± 0.52	Unexcavated	Antineoplastic activity [63]
**33**	10.37	(±)-balanophonin	C_20_H_20_O_6_	355.1190	355.1187	0.70	355.1180, 175.0391, 147.0441, 119.0490	1.31 ± 0.01	37.29 ± 2.96	Unexcavated	Neuroprotection [64]
**34**	10.86	naringenin	C_15_H_12_O_5_	271.0615	271.0612	1.08	271.0609, 151.0026, 119.0491, 65.0020	0.28 ± 0.00	30.40 ± 1.28	Unexcavated	Neuroprotection [65]
**35**	10.86	luteolin	C_15_H_10_O_6_	285.0404	285.0404	0.01	285.0401, 199.0392, 133.0284, 65.0020	0.31 ± 0.07	33.79 ± 1.37		Anti-inflammation [66] Antithrombosis [67]
**36**	11.27	kaempferol	C_15_H_10_O_6_	285.0408	285.0405	1.09	285.0406, 211.0388, 159.0441, 117.0334, 93.033	0.03 ± 0.00	39.44 ± 2.72	Unexcavated	Antibacteriality [68]
**37**	11.41	apigenin	C_15_H_10_O_5_	269.0458	269.0456	0.84	269.0451, 151.0025, 117.0334, 65.0020	0.06 ± 0.00	34.24 ± 2.77		Antineoplastic activity [69]
**38**	12.75	6-gingerol	C_17_H_26_O_4_	293.1762	293.1758	1.40	236.1049, 221.1541, 205.1228, 148.0520	42.67 ± 0.16	0.00 ± 1.99	Unexcavated	Anti-inflammation [70]
**39**	14.22	curcumol *	C_15_H_24_O_2_	237.1840	237.1849	3.68	237.1854, 181.085, 107.0496, 57.0708	0.65 ± 0.08	0.00 ± 1.93	Unexcavated	Antiviral activity [71], Cardioprotection [72]
**40**	15.11	α-Linolenic acid	C_18_H_30_O_2_	277.2175	277.2173	0.83	277.2171, 139.0811	20.61 ± 1.84	0.00 ± 1.99	Unexcavated	Anti-inflammation [73]
**41**	15.36	linoleic acid	C_18_H_32_O_2_	279.2329	279.2330	0.03	279.2329	15.92 ± 0.29	0.00 ± 2.06		Immunomodulation [74]
**42**	15.54	phillygenin #	C_21_H_24_O_6_	371.1534	371.1500	9.17	371.1525, 339.1631, 115.9197, 100.9325	29.53 ± 9.35	52.09 ± 3.40	Unexcavated	Anti-inflammation [75]
**43**	15.56	palmitic acid	C_16_H_32_O_2_	255.2330	255.2330	0.26	255.2328	0.27 ± 0.03	0.00 ± 0.71		Antineoplastic activity [76]
**44**	15.62	oleic acid	C_18_H_34_O_2_	281.2488	281.2486	0.85	281.2481, 170.5686, 66.2491	18.89 ± 2.87	0.00 ± 0.52	Unexcavated	Antioxidation [77]
**45**	16.15	ethyl Stearate	C_20_H_40_O_2_	311.2958	311.2956	0.83	183.0114, 119.0493, 79.9562	0.59 ± 0.07	3.16 ± 1.74	Unexcavated	Neuroprotection [78]
**46**	16.77	(+)-4-cholesten-3-one *	C_27_H_44_O	385.3472	385.3465	1.92	385.3459, 109.0651, 97.0652, 79.0548, 67.0550	37.13 ± 3.85	83.50 ± 3.59	Unexcavated	Antineoplastic activity [79]

* Note: Detailed information on the original MS spectra and the identification process are provided in Supplementary Data S2. Although the scan mode was set to a range of *m/z* 100–1500, the Xcalibur 4.1 Software package detected *m/z* values below 50. Compounds labeled with “*” were identified using the positive ion mode, whereas those without “*” were identified under the negative ion mode. “#” indicates Q-marker. Antioxidant percentages are presented as mean ± standard deviation (SD) from three replicates (*n* = 3). Chemical content is also reported as mean ± SD from three replicates (*n* = 3) with units in mg/g extract. Quantitative analysis was used to determine the chemical content.

## Data Availability

The original contributions presented in this study are included in the article/Supplementary Materials. Further inquiries can be directed to the corresponding authors.

## Supplementary Materials

The following supporting information can be downloaded at: https://www.mdpi.com/article/10.3390/molecules30030722/s1. Supplementary Data S1: Information of all authentic standards. Supplementary Data S2: MS spectra and identification of **1**–**46** (Figures S1–S46, see [99]). Supplementary Data S3: Quantification curves. Table S1: Quantification of compounds. Figure S47: A typical linear regression curve (taking gallic acid as the example).

## References

[1] Aiyelaagbe O.O., Adesogan K., Ekundayo O., Gloer J.B. (2007). Antibacterial Diterpenoids from *Jatropha podagrica* Hook. Phytochemistry.

[2] Yuan H.T., Li Q.F., Tian T., Zhang C.Y., Huang Z.Q., Fan C.X., Mei K., Zhou J., Zhai X.X., Li S.B. (2021). Lathyrane Diterpenoids from *Jatropha podagrica* and Their Antitumor Activities in Human Osteosarcoma Cells. Nat. Prod. Res..

[3] Sabandar C.W., Ahmat N., Jaafar F.M., Sahidin I. (2013). Medicinal Property, Phytochemistry and Pharmacology of Several *Jatropha* Species (Euphorbiaceae): A Review. Phytochemistry.

[4] Aiyelaagbe O.O., Gloer J.B. (2008). Japodic Acid, A Novel Aliphatic Acid from *Jatropha podagrica* Hook. Rec. Nat. Prod..

[5] Ghali W., Vaudry D., Jouenne T., Marzouki M.N. (2013). Assessment of Cyto-Protective, Antiproliferative and Antioxidant Potential of a Medicinal Plant *Jatropha podagrica*. Ind. Crops Prod..

[6] Gübitz G.M., Mittelbach M., Trabi M. (1999). Exploitation of the Tropical Oil Seed Plant *Jatropha curcas* L. Bioresour. Technol..

[7] Premjet D., Obeng A.K., Yoo H.Y., Kim S.W., Premjet S. (2021). Physicochemical Characterization of *Jatropha podagrica* Seed Oil for Potential Biodiesel Production and Other Industrial Applications in Thailand. Sains Malays..

[8] Yin Z.H., Zhang J.J., Kang W.Y. (2017). Volatile Composition of *Jatropha podagrica* Seeds and Flowers. Chem. Nat. Compd..

[9] Jang Y.S., Lee D.E., Ju D.U., Jeong S.Y., Ko Y.J., Pang C.H., Kang K.S., Gwon H.J., Yoo H.M., Kim K.H. (2023). Antiviral Effects of Secondary Metabolites from *Jatropha podagrica* Leaves against the Pseudotyped Virus of SARS-CoV-2 Omicron. Plants.

[10] Minh T.N., Xuan T.D., Tran H.D., Van T.M., Andriana Y., Khanh T.D., Quan N.V., Ahmad A. (2019). Isolation and Purification of Bioactive Compounds from the Stem Bark of *Jatropha podagrica*. Molecules.

[11] Khattar N., Sharma R., Kaur A., Chhabra R. (2024). Phytochemical Profile and GC–MS Analysis from Different Parts of Terminalia Chebula Retz. and *Terminalia bellirica* (Gaertn.) Roxb. Vegetos.

[12] Nigam M., Mishra A.P., Adhikari-Devkota A., Dirar A.I., Hassan M.M., Adhikari A., Belwal T., Devkota H.P. (2020). Fruits of *Terminalia chebula* Retz.: A Review on Traditional Uses, Bioactive Chemical Constituents and Pharmacological Activities. Phytother. Res. PTR.

[13] Przybylska D., Kucharska A.Z., Sozański T. (2023). A Review on Bioactive Iridoids in Edible Fruits—From Garden to Food and Pharmaceutical Products. Food Rev. Int..

[14] Nelson A.S., Whitehead S.R. (2021). Fruit Secondary Metabolites Shape Seed Dispersal Effectiveness. Trends Ecol. Evol..

[15] Hassane A.M.A., Hussien S.M., Abouelela M.E., Taha T.M., Awad M.F., Mohamed H., Hassan M.M., Hassan M.H.A., Abo-Dahab N.F., El-Shanawany A.R.A. (2022). In Vitro and In Silico Antioxidant Efficiency of Bio-Potent Secondary Metabolites From Different Taxa of Black Seed-Producing Plants and Their Derived Mycoendophytes. Front. Bioeng. Biotechnol..

[16] Chen S., Li X., Zeng J., Cai R., Li C., Chen C. (2023). Library-Based UHPLC-Q-Exactive-Orbitrap-MS Putative Identification of Isomeric and Non-Isomeric Bioactives from Zibushengfa Tablet and Pharmacopeia Quality-Marker Chemistry. J. Liq. Chromatogr. Relat. Technol..

[17] Li X., Chen S., Zeng J., Cai R., Liang Y., Chen C., Chen B., Li C. (2023). Database-Aided UHPLC-Q-Orbitrap MS/MS Strategy Putatively Identifies 52 Compounds from Wushicha Granule to Propose Anti-Counterfeiting Quality-Markers for Pharmacopoeia. Chin. Med..

[18] Lin R., Li X., Liu S., Cai R., Zeng J., Luo Z., He J. (2024). Integrated UHPLC-Q-Exactive-Orbitrap MS/MS and ABTS Analyses Screen Antioxidant and Anti-Counterfeiting Marker of Eucommiae Cortex (Duzhong) for Pharmacopoeias. Microchem. J..

[19] Li X.C., Zeng J.Y., Li C.H., Chai H.X., Chen S.M., Jin N.N., Chen T.S., Lin X.H., Khan S.B., Cai R.X. (2024). Simultaneous Qualitative and Quantitative Determination of 33 Compounds from *Rubus alceifolius* Poir Leaves Using UHPLC-Q-Orbitrap-MS/MSAnalysis. Curr. Anal. Chem..

[20] Zheng G.P. (2004). Determination of Emodin in Zibushengfa Tablets by RP-HPLC. Her. Med..

[21] Fu S., Cheng R.R., Deng Z.X., Liu T.G. (2021). Qualitative Analysis of Chemical Components in Lianhua Qingwen Capsule by HPLC-Q Exactive-Orbitrap-MS Coupled with GC-MS. J. Pharm. Anal..

[22] Wang S., Sun X.Z., An S., Sang F., Zhao Y.L., Yu Z.G. (2021). High-Throughput Identification of Organic Compounds from Polygoni multiflori Radix Praeparata (*Zhiheshouwu*) by UHPLC-Q-Exactive Orbitrap-MS. Molecules.

[23] Qian Z.M., Li H.J., Li P., Ren M.T., Tang D. (2007). Simultaneous Qualitation and Quantification of Thirteen Bioactive Compounds in Flos Lonicerae by High-Performance Liquid Chromatography with Diode Array Detector and Mass Spectrometry. Chem. Pharm. Bull..

[24] Fan R., Peng C., Zhang X.X., Qiu D.Y., Mao G.L., Lu Y.S., Zeng J.W. (2020). A Comparative UPLC-Q-Orbitrap-MS Untargeted Metabolomics Investigation of Different Parts of *Clausena lansium* (Lour.) Skeels. Food Sci. Nutr..

[25] Qiao X., Li R., Song W., Miao W.J., Liu J., Chen H., Guo D.A., Ye M. (2016). A Targeted Strategy to Analyze Untargeted Mass Spectral Data: Rapid Chemical Profiling of *Scutellaria baicalensis* Using Ultra-High Performance Liquid Chromatography Coupled with Hybrid Quadrupole Orbitrap Mass Spectrometry and Key Ion Filtering. J. Chromatogr. A.

[26] Gao X.X., Wang N., Jia J.P., Wang P.Y., Zhang A.R., Qin X.M. (2020). Chemical Profliling of Dingkun Dan by Ultra High Performance Liquid Chromatography Q Exactive Orbitrap High Resolution Mass Spectrometry. J. Pharm. Biomed. Anal..

[27] Li X.C., Zeng J.Y., Cai R.X., Li C.H., Chen X.S., Chen B., Zhao X.J., Khan S.B. (2025). Putative Identification of 47 Compounds from Jieyu Anshen Granule and Proposal of Pharmacopeia Quality-Assessment Strategy Using TCM-Specific Library with UHPLC-Q-Exactive-Orbitrap-MS. ChemistryOpen.

[28] Hassanpour S., Rezaei H., Razavi S.M. (2020). Anti-Nociceptive and Antioxidant Activity of Betaine on Formalin- and Writhing Tests Induced Pain in Mice. Behav. Brain Res..

[29] Li S., Cai Y.W., Guan T., Zhang Y., Huang K., Zhang Z., Cao W.Q., Guan X. (2024). Quinic Acid Alleviates High-Fat Diet-Induced Neuroinflammation by Inhibiting DR3/IKK/NF-κB Signaling via Gut Microbial Tryptophan Metabolites. Gut Microbes.

[30] Ament Z., Bevers M.B., Wolcott Z., Kimberly W.T., Acharjee A. (2021). Uric Acid and Gluconic Acid as Predictors of Hyperglycemia and Cytotoxic Injury after Stroke. Transl. Stroke Res..

[31] Koriem K., Tharwat H. (2023). Malic Acid Improves Behavioral, Biochemical, and Molecular Disturbances in the Hypothalamus of Stressed Rats. J. Integr. Neurosci..

[32] Tang X.L., Liu J.X., Dong W., Li P., Li L., Lin C.R., Zheng Y., Hou J.C., Li D. (2013). The Cardioprotective Effects of Citric Acid and L-Malic Acid on Myocardial Ischemia/Reperfusion Injury. Evid. Based Complement. Alternat. Med..

[33] Yamada J., Bueno M., Santos L., Haliburton S., Marsha C.Y., Stevens B. (2023). Sucrose Analgesia for Heel-Lance Procedures in Neonates. Cochrane Database Syst. Rev..

[34] Xiao X.Q., Liu G.Q. (1999). L-Pyroglutamic Acid Protects Rat Cortical Neurons against Sodium Glutamate-Induced Injury. Zhongguo Yao Li Xue Bao.

[35] Abarikwu S.O., Mgbudom-Okah C.J., Njoku R.C., Okonkwo C.J., Onuoha C.C., Wokoma A.F.S. (2022). Gallic Acid Ameliorates Busulfan-Induced Testicular Toxicity and Damage in Mature Rats. Drug Chem. Toxicol..

[36] Maesaka E., Kukuminato S., Aonishi k., Koyama K., Koseki S. (2023). Antibacterial Effect of Melanoidins Derived From Xylose and Phenylalanine Against *Bacillus cereus* and *Clostridium perfringens*. J. Food Prot..

[37] Guo Y.X., Zhang Q., Zuo Z.H., Chu J., Xiao H.Z., Javed M.T., He C. (2018). Protocatechuic Acid (PCA) Induced a Better Antiviral Effect by Immune Enhancement in SPF Chickens. Microb. Pathog..

[38] Anjum R., Maheshwari N., Mahmood R. (2022). 3,4-Dihydroxybenzaldehyde Mitigates Fluoride-Induced Cytotoxicity and Oxidative Damage in Human RBC. J. Trace Elem. Med. Biol..

[39] Bernatoniene J., Kopustinskiene D.M. (2018). The Role of Catechins in Cellular Responses to Oxidative Stress. Molecules.

[40] Li Y.F., Ouyang S.H., Tu L.F., Wang X., Yuan W.L., Wang G.E., Wu Y.P., Duan W.J., Yu H.M., Fang Z.Z. (2018). Caffeine Protects Skin from Oxidative Stress-Induced Senescence through the Activation of Autophagy. Theranostics.

[41] Li W.F., Li W.Q., Yu J.J., Liu F., Zang L.L., Xiao X., Zhao J.M., Yao Q., Niu X.F. (2020). Fraxin Inhibits Lipopolysaccharide-Induced Inflammatory Cytokines and Protects against Endotoxic Shock in Mice. Fundam. Clin. Pharmacol..

[42] Ma X., Li M.M., Lu G.C., Wang R.H., Wei Y.M., Guo Y.F., Yu Y.X., Jiang C.D. (2021). Anti-Inflammation of Epicatechin Mediated by TMEM35A and TMPO in Bovine Mammary Epithelial Cell Line Cells and Mouse Mammary Gland. J. Dairy Sci..

[43] Qu L.C., Lin P., Lin M.J., Ye S.M., Akuetteh P.D.P., Zhu Y.Y. (2021). Fraxetin Inhibits the Proliferation and Metastasis of Glioma Cells by Inactivating JAK2/STAT3 Signaling. Evid.-Based Complement. Altern. Med. ECAM.

[44] Luo T.Y., Zhou X.Y., Qin M.Y., Lin Y.Q., Lin J.F., Chen G.P., Liu A.J., Ouyang D.Y., Chen D.F., Pan H. (2022). Corilagin Restrains NLRP3 Inflammasome Activation and Pyroptosis through the ROS/TXNIP/NLRP3 Pathway to Prevent Inflammation. Oxid. Med. Cell. Longev..

[45] Chougule P.R., Sangaraju R., Patil P.B., Qadri S.S.Y.H., Panpatil V.V., Ghosh S., Mungamuri S.K., Bhanoori M., Sinha S.N. (2023). Effect of Ethyl Gallate and Propyl Gallate on Dextran Sulfate Sodium (DSS)-Induced Ulcerative Colitis in C57BL/6 J Mice: Preventive and Protective. Inflammopharmacology.

[46] Ahn D., Kim J., Nam G., Zhao X., Kwon J., Hwang J.Y., Kim J.K., Yoon S.Y., Chung S.J. (2022). Ethyl Gallate Dual-Targeting PTPN6 and PPARγ Shows Anti-Diabetic and Anti-Obese Effects. Int. J. Mol. Sci..

[47] Bak S.G., Lim H.J., Won Y.S., Lee S., Cheong S.H., Lee S.J., Bae E.Y., Lee S.W., Lee S.J., Rho M.C. (2022). Regulatory Effects of *Lycium barbarum* Extract and Isolated Scopoletin on Atopic Dermatitis-Like Skin Inflammation. BioMed Res. Int..

[48] Zhang D., Jing B., Chen Z.N., Li X., Shi H.M., Zheng Y.C., Chang S.Q., Gao L., Zhao G.P. (2023). Ferulic Acid Alleviates Sciatica by Inhibiting Neuroinflammation and Promoting Nerve Repair via the TLR4/NF-κB Pathway. CNS Neurosci. Ther..

[49] Wang X.R., An F., Wang S.L., An Z.X., Wang S.H. (2017). Orientin Attenuates Cerebral Ischemia/Reperfusion Injury in Rat Model through the AQP-4 and TLR4/NF-κB/TNF-α Signaling Pathway. J. Stroke Cerebrovasc. Dis..

[50] Tao J.Y., Li J., Wan L., Dong B.Z., Yu Y.J., Liu Y.M., Yi M.L., Wan L.P. (2023). Orientin Regulates the Proliferation and Migration of Hepatocellular Carcinoma Cells. Naunyn. Schmiedebergs Arch. Pharmacol..

[51] Yi Y., Zhang M., Xue H., Yu R., Bao Y.O., Kuang Y., Chai Y., Ma W., Wang J., Shi X.M. (2022). Schaftoside Inhibits 3CLpro and PLpro of SARS-CoV-2 Virus and Regulates Immune Response and Inflammation of Host Cells for the Treatment of COVID-19. Acta Pharm. Sin. B.

[52] Li Y.G., Zhao Y.J., Tan X.Q., Liu J.Y., Zhi Y.K., Yi L., Bai S.S., Du Q., Li Q.X., Dong Y. (2020). Isoorientin Inhibits Inflammation in Macrophages and Endotoxemia Mice by Regulating Glycogen Synthase Kinase 3β. Mediators Inflamm..

[53] Dong Y.Q., Stewart T., Bai L.D., Li X., Xu T., Iliff J., Shi M., Zheng D.F., Yuan L., Wei T.T. (2020). Coniferaldehyde Attenuates Alzheimer’s Pathology via Activation of Nrf2 and Its Targets. Theranostics.

[54] Guan S.Y., Sun L.B., Wang X.H., Huang X.R., Luo T. (2022). Isoschaftoside Inhibits Lipopolysaccharide-Induced Inflammation in Microglia through Regulation of HIF-1α-Mediated Metabolic Reprogramming. Evid.-Based Complement. Altern. Med. ECAM.

[55] Mustapha M., Mat Taib C.N. (2023). Beneficial Role of Vitexin in Parkinson’s Disease. Malays. J. Med. Sci. MJMS.

[56] Ahn J.S., Lee C.H., Liu X.Q., Hwang K.W., Oh M.H., Park S.Y., Whang W.K. (2024). Neuroprotective Effects of Phenolic Constituents from Drynariae Rhizoma. Pharmaceuticals.

[57] Shen J., Yang L.J., You K., Chen T., Su Z.H., Cui Z.F., Wang M., Zhang W.C., Liu B., Zhou K. (2022). Indole-3-Acetic Acid Alters Intestinal Microbiota and Alleviates Ankylosing Spondylitis in Mice. Front. Immunol..

[58] Li J.X., Shang L., Zhou F.Y., Wang S.H., Liu N., Zhou M.F., Lin Q.F., Zhang M.Q., Cai Y.J., Chen G. (2023). *Herba patriniae* and Its Component Isovitexin Show Anti-Colorectal Cancer Effects by Inducing Apoptosis and Cell-Cycle Arrest via P53 Activation. Biomed. Pharmacother..

[59] Lee H.S., Jeong G.S. (2020). Aromadendrin Inhibits T Cell Activation via Regulation of Calcium Influx and NFAT Activity. Molecules.

[60] Cui S.M., Cui Y.Q., Li Y., Zhang Y.T., Wang H., Qin W.D., Chen X.M., Ding S.F., Wu D.W., Guo H.P. (2018). Inhibition of Cardiac Hypertrophy by Aromadendrin through Down-Regulating NFAT and MAPKs Pathways. Biochem. Biophys. Res. Commun..

[61] Liu B.B., Deng X.L., Jiang Q.Q., Li G.X., Zhang J.L., Zhang N., Xin S.L., Xu K.S. (2020). Scoparone Improves Hepatic Inflammation and Autophagy in Mice with Nonalcoholic Steatohepatitis by Regulating the ROS/P38/Nrf2 Axis and PI3K/AKT/mTOR Pathway in Macrophages. Biomed. Pharmacother..

[62] Lu G.Y., Wang X.Z., Cheng M., Wang S.J., Ma K. (2023). The Multifaceted Mechanisms of Ellagic Acid in the Treatment of Tumors: State-of-the-Art. Biomed. Pharmacother..

[63] Ramesh E., Alshatwi A.A. (2013). Naringin Induces Death Receptor and Mitochondria-Mediated Apoptosis in Human Cervical Cancer (SiHa) Cells. Food Chem. Toxicol..

[64] Lim S.Y., Subedi L., Shin D.Y., Kim C.S., Lee K.R., Kim S.Y. (2017). A New Neolignan Derivative, Balanophonin Isolated from Firmiana Simplex Delays the Progress of Neuronal Cell Death by Inhibiting Microglial Activation. Biomol. Ther..

[65] Mohamed E.I., Zaki M.A., Chaurasiya N.D., Owis A.I., AbouZid S., Wang Y.H., Avula B., Seida A.A., Tekwani B.L., Ross S.A. (2018). Monoamine Oxidases Inhibitors from *Colvillea racemosa*: Isolation, Biological Evaluation, and Computational Study. Fitoterapia.

[66] Li B.L., Du P.L., Du Y., Zhao D.Y., Cai Y.R., Yang Q., Guo Z.J. (2021). Luteolin Alleviates Inflammation and Modulates Gut Microbiota in Ulcerative Colitis Rats. Life Sci..

[67] Chen T.R., Wei L.H., Guan X.Q., Huang C., Liu Z.Y., Wang F.J., Hou J., Jin Q., Liu Y.F., Wen P.H. (2019). Biflavones from Ginkgo Biloba as Inhibitors of Human Thrombin. Bioorganic Chem..

[68] Periferakis A., Periferakis K., Badarau I.A., Petran E.M., Popa D.C., Caruntu A., Costache R.S., Scheau C., Caruntu C., Costache D.O. (2022). Kaempferol: Antimicrobial Properties, Sources, Clinical, and Traditional Applications. Int. J. Mol. Sci..

[69] Imran M., Tanweer A.G., Atif M., Shahbaz M., Batool Q.T., Muhammad H.M., Salehi B., Martorell M., Javad S.R. (2020). Apigenin as an Anticancer Agent. Phytother. Res..

[70] Wu S.L., Zhu J.X., Wu G.H., Hu Z.Q., Ying P.X., Bao Z.J., Ding Z.P., Tan X.R. (2022). 6-Gingerol Alleviates Ferroptosis and Inflammation of Diabetic Cardiomyopathy via the Nrf2/HO-1 Pathway. Oxid. Med. Cell. Longev..

[71] Zheng J.G., Xu Y.L., Khan A., Sun P.P., Sun Y.G., Fan K.H., Yin W., Wang S.Y., Li H.Q., Sun N. (2021). Curcumol Inhibits Encephalomyocarditis Virus by Promoting IFN-β Secretion. BMC Vet. Res..

[72] Fang Z., Li S., Yushanjiang F., Feng G., Cui S.Y., Hu S., Jiang X.J., Liu C.J. (2023). Curcumol Alleviates Cardiac Remodeling via the AKT/NF-κB Pathway. Int. Immunopharmacol..

[73] Yang J., Liu S.H., Zhao Q., Li X.B., Jiang K.F. (2023). Gut Microbiota-Related Metabolite α-Linolenic Acid Mitigates Intestinal Inflammation Induced by Oral Infection with *Toxoplasma gondii*. Microbiome.

[74] Nava Lauson C.B., Tiberti S., Corsetto P.A., Conte F., Tyagi P., Machwirth M., Ebert S., Loffreda A., Scheller L., Sheta D. (2023). Linoleic Acid Potentiates CD8+ T Cell Metabolic Fitness and Antitumor Immunity. Cell Metab..

[75] Zhou M.T., Tang Y.Q., Liao L., Liu M.C., Deng Y., Zhao X.T., Li Y.X. (2021). Phillygenin Inhibited LPS-Induced RAW 264.7 Cell Inflammation by NF-κB Pathway. Eur. J. Pharmacol..

[76] Zhu S., Jiao W.H., Xu Y.L., Hou L.J., Li H., Shao J.R., Zhang X.L., Wang R., Kong D.X. (2021). Palmitic Acid Inhibits Prostate Cancer Cell Proliferation and Metastasis by Suppressing the PI3K/Akt Pathway. Life Sci..

[77] Mann J., Reznik E., Santer M., Fongheiser M.A., Smith N., Hirschhorn T., Zandkarimi F., Soni R.K., Dafré A.L., Antonio M.V. (2024). Ferroptosis Inhibition by Oleic Acid Mitigates Iron-Overload-Induced Injury. Cell Chem. Biol..

[78] Yi L., Ma H.S., Yang X.X., Zheng Q., Zhong J., Ye S., Li X.C., Chen D.F., Li H., Li C.X. (2024). Cotransplantation of NSCs and Ethyl Stearate Promotes Synaptic Plasticity in PD Rats by Drd1/ERK/AP-1 Signaling Pathway. J. Ethnopharmacol..

[79] Ma J.B., Fu G.B., Wu J., Han S.X., Zhang L.S., Yang M., Yu Y., Zhang M.Y., Lin Y.L., Wang Y.B. (2016). 4-Cholesten-3-One Suppresses Lung Adenocarcinoma Metastasis by Regulating Translocation of HMGB1, HIF1α and Caveolin-1. Cell Death Dis..

[80] Lee D., Lee S.Y., Ra M.J., Chen S.M., Yu J.N., Kang K.S., Kim K.H. (2024). Cancer Therapeutic Potential of Hovetrichoside C from *Jatropha podagrica* on Apoptosis of MDA-MB-231 Human Breast Cancer Cells. Food Chem. Toxicol..

[81] Huynh L., Nguyen T.N.T., Nguyen X.A.N., Tran A.D.L., Nguyen L.T.H., Van K.T.P., Tran M.H. (2024). A Mini Review on Botany, Phytochemistry, and Bioactivities of *Jatropha podagrica* Hook. (Euphorbiaceae). Trop. J. Nat. Prod. Res..

[82] Aubert C., Milhet C. (2007). Distribution of the Volatile Compounds in the Different Parts of a White-Fleshed Peach (*Prunus persica* L. Batsch). Food Chem..

[83] Khallouki F., Haubner R., Erben G., Ulrich C.M., Owen R.W. (2012). Phytochemical Composition and Antioxidant Capacity of Various Botanical Parts of the Fruits of *Prunus* × *Domestica* L. from the Lorraine Region of Europe. Food Chem..

[84] Li X.C., Liu J., Chen B., Chen Y.C., Dai W.J., Li Y.L., Zhu M.L. (2020). Covalent Bridging of Corilagin Improves Antiferroptosis Activity: Comparison with 1,3,6-tri- O -galloyl-β- D -glucopyranose. ACS Med. Chem. Lett..

[85] Golmohammadi M., Zamanian M.Y., Jalal S.M., Noraldeen S.A.M., Ramírez Coronel A.A., Oudaha K.H., Obaid R.F., Almulla A.F., Bazmandegan G., Kamiab Z. (2023). A Comprehensive Review on Ellagic Acid in Breast Cancer Treatment: From Cellular Effects to Molecular Mechanisms of Action. Food Sci. Nutr..

[86] Zhu H.Y., Yan Y.M., Jiang Y., Meng X.F. (2022). Ellagic Acid and Its Anti-Aging Effects on Central Nervous System. Int. J. Mol. Sci..

[87] Zhang T.J., Bai G., Chen C.Q., Xu J., Han Y.Q., Gong S.X., Zhang H.B., Liu C. (2018). Research Approaches of Quality Marker (Q-Marker) of Chinese Materia Medica Formula Based on “Five Principles”. Chin. Tradit. Herb. Drugs.

[88] Xu Y.H., Chen X.Y., Chen J. (2024). An Integrated Strategy for Deciphering Quality Markers of *Terminaliae Belliricae Fructus* Based on a Three-Dimensional Characteristic Model. J. Chromatogr. A.

[89] Zeng J., Li X., Cai R., Chen B., Li C., Hu Q., Sun Y. (2024). Proposing Anti-Counterfeiting Pharmacopoeia Quality Markers for Shenlingbaizhu Granule Based on UHPLC-Q-Orbitrap-MS Identification. Phytochem. Anal. PCA.

[90] Jung M.Y., Choi D.S. (2016). Protective Effect of Gallic Acid on the Thermal Oxidation of Corn and Soybean Oils during High Temperature Heating. Food Sci. Biotechnol..

[91] Wang J.H., Ni S.F., Zheng K., Zhao Y., Zhang P.H., Chang H. (2024). Phillygenin Alleviates Arthritis through the Inhibition of the NLRP3 Inflammasome and Ferroptosis by AMPK. Crit. Rev. Immunol..

[92] Chinese Pharmacopoeia Commission. https://english.nmpa.gov.cn/2019-07/19/c_389168.htm.

[93] Zhou Q., Dai Y.P., Sun L.L. (2014). Study on Content Variation of Gallic Acid and Ellagic Acid in Course of Processing of Charred Granati Pericarpium. Zhongguo Zhong Yao Za Zhi Zhongguo Zhongyao Zazhi China J. Chin. Mater. Medica.

[94] Li X. (2013). Solvent Effects and Improvements in the Deoxyribose Degradation Assay for Hydroxyl Radical-Scavenging. Food Chem..

[95] Li X.C. (2017). 2-Phenyl-4,4,5,5-Tetramethylimidazoline-1-Oxyl 3-Oxide (PTIO^•^) Radical Scavenging: A New and Simple Antioxidant Assay In Vitro. J. Agric. Food Chem..

[96] Cai R., Li X., Li C., Zhu J., Zeng J., Li J., Tang B., Li Z., Liu S., Yan Y. (2022). Standards-Based UPLC-Q-Exactive Orbitrap MS Systematically Identifies 36 Bioactive Compounds in *Ampelopsis grossedentata* (Vine Tea). Separations.

[97] Pritchard B.P., Altarawy D., Didier B., Gibson T.D., Windus T.L. (2019). New Basis Set Exchange: An Open, Up-to-Date Resource for the Molecular Sciences Community. J. Chem. Inf. Model..

[98] Xie Y., Li X., Xu J., Jiang Q., Xie H., He J., Chen D. (2017). Two Phenolic Antioxidants in Suoyang Enhance Viability of ^•^OH-Damaged Mesenchymal Stem Cells: Comparison and Mechanistic Chemistry. Chem. Cent. J..

[99] Gross J.H. (2013). Mass Spectrometry.

