# Supplementary material for: High-Resolution LC-MS Simultaneous Quantification of Forty-Six Compounds from Jatropha podagrica Fruit Recommends Four Top Antioxidant Contributors as Q-Markers
Source: Molecules. 2025 Feb 5;30(3):722. doi: 10.3390/molecules30030722 (PMC11821128; doi:10.3390/molecules30030722)
Supplement: Supplementary file 1 [file molecules-30-00722-s001.zip › Supplementary data S1.pdf]

Suppl. S1 Information of all authentic standards

Quinic acid (Cas. 77-95-2, $C_7H_{12}O_6$, M.W. 192.167), betaine (Cas. 107-43-7, $C_5H_{11}NO_2$, M.W. 117.148), malic acid (Cas. 6915-15-7, $C_4H_6O_5$, M.W. 134.087), pyroglutamic acid(Cas. 98-79-3, $C_5H_7NO_3$, M.W. 129.115), 3,4-dihydroxybenzaldehyde (Cas. 139-85-5,$C_7H_6O_3$, M.W. 138.122), fraxin (CAS. 524-30-1, $C_{16}H_{18}O_{10}$, M.W. 370.31), isoorientin (CAS. 4261-42-1, $C_{21}H_{20}O_{11}$, M.W. 448.38), neoeriocitrin(Cas. 13241-32-2, $C_{27}H_{32}O_{15}$, M.W. 596.538), indole-3-acetic acid (Cas. 87-51-4, $C_{10}H_9NO_2$, M.W. 175.187), aromadendrin (Cas. 480-20-6, $C_{15}H_{12}O_6$, M.W. 288.3), scoparone (Cas. 120-08-1, $C_{11}H_{10}O_4$, M.W.206.197), apigenin (Cas. 520-36-5, $C_{15}H_{10}O_5$, M.W. 270.24), curcumol (Cas. 4871-97-0, $C_{15}H_{24}O_2$, M.W. 236.355), α-Linolenic acid (Cas. 463-40-1, $C_{18}H_{30}O_2$, M.W. 278.436), linoleic Acid (Cas. 104777-68-6, $C_{18}H_{32}O_2$, M.W. 280.452), phillygenin (Cas. 487-39-8, $C_{21}H_{24}O_6$, M.W. 372.417), palmitic Acid (Cas. 57-10-3, $C_{16}H_{32}O_2$ M.W. 256.43) and oleic acid (Cas. 112-80-1, $C_{18}H_{34}O_2$, M.W. 282.468) were obtained from Baoji Herbest Bio-Tech Co., Ltd. Fraxetin (Cas. 574-84-5, $C_{10}H_8O_5$, M.W. 208.17), corilagin(Cas. 23094-69-1, $C_{27}H_{22}O_{18}$, M.W. 634.45), coniferaldehyde (Cas. 20649-42-7, $C_{10}H_{10}O_3$, M.W.178.18) and luteolin (Cas. 491-70-3, $C_{15}H_{10}O_6$, M.W. 286.24) were obtained from BioBioPha Co., Ltd. (Kunming, China). Gallic acid (Cas. 149-91-7, $C_7H_6O_5$ M.W. 170.12), ethyl gallate (Cas. 831-61-8, $C_9H_{10}O_5$, M.W. 198.17), orientin (Cas. 28608-75-5, $C_{21}H_{20}O_{11}$, M.W. 448.38), isovitexin(Cas. 38953-85-4, $C_{21}H_{20}O_{10}$, M.W. 432.3775), ellagic acid (Cas. 476-66-4, $C_{14}H_6O_8$, M.W. 302.28), naringin (Cas. 10236-47-2, $C_{27}H_{32}O_{14}$, M.W.580.53) and 6-gingerol (Cas. 23513-14-6, $C_{17}H_{26}O_3$, M.W. 293.39 were obtained from Chengdu Alfa Biotechnology Co., Ltd. (Chengdu, China). Protocatechuic acid (Cas. 99-50-3, $C_7H_6O_4$ M.W. 154.12), (-)-epicatechin (Cas. 490-46-0, $C_{15}H_{14}O_6$, M.W. 290.27), scopoletin (Cas. 92-61-5, $C_{10}H_8O_4$, M.W. 192.17), schaftoside (Cas. 51938-32-0, $C_{26}H_{28}O_{14}$, M.W. 564.5),

isoschaftoside (Cas. 52012-29-0, $C_{26}H_{28}O_{14}$, M.W. 564.49), Vitexin (Cas. 3681-93-4, $C_{21}H_{10}O_{10}$, M.W. 432.11), (±)-balanophonin(Cas. 118916-57-7, $C_{20}H_{20}O_6$ M.W.356.37) and naringenin (Cas. 480-41-1, $C_{15}H_{12}O_5$, M.W.272.25 were obtained from SichuanWeikeqi BiologicalTechnologyCo., Ltd. (Chengdu, China). D-gluconic acid (Cas. 526-95-4, $C_6H_{11}O_7$, M.W. 195.1479) was obtained from Sigma-Aldrich (Shanghai, China). Kaempferol (Cas. 520-18-3, $C_{15}H_{10}O_6$, M.W. 286.24) was obtained from Aladdin Chemistry Co. (Shanghai, China). Ethyl stearate (Cas. 111-61-5, $C_{20}H_{40}O_2$, M.W. 312.5) was obtained from GuangZhou Sopo Biological Technology Co.,LTD. L-phenylalanine(Cas. 63-91-2, $C_9H_{11}NO_2$, M.W. 165) was obtained from J&K Scientific Co., Ltd. (Beijing, China). (-)-catechin (Cas. 18829-70-4, $C_{15}H_{14}O_6$, M.W. 290.27) was obtained from Shaanxi Herbest, Biotech. Co. Ltd. (Boji, China). Ferulic acid (Cas. 1135-24-6, $C_{10}H_{10}O_4$ M.W. 194.19) was obtained from Chinese Medicine Solid State Manufacturing Technology (Nanchang, China). (+)-4-cholesten-3-one (Cas. 601-57-0, $C_{27}H_{44}O$, M.W. 384.65) was obtained from TCI Chemical Co. (Shanghai, China).
