# Supplementary material for: High-Resolution LC-MS Simultaneous Quantification of Forty-Six Compounds from Jatropha podagrica Fruit Recommends Four Top Antioxidant Contributors as Q-Markers
Source: Molecules. 2025 Feb 5;30(3):722. doi: 10.3390/molecules30030722 (PMC11821128; doi:10.3390/molecules30030722)
Supplement: Supplementary file 1 [file molecules-30-00722-s001.zip › Supplementary data S2.pdf]

Suppl. S2 **Identification of 46 compounds (1-46) based on MS comparison**

Suppl. S2.1 Identification of betaine (Cas. 107-43-7, $C_5H_{11}NO_2$, M.W. 117.148).

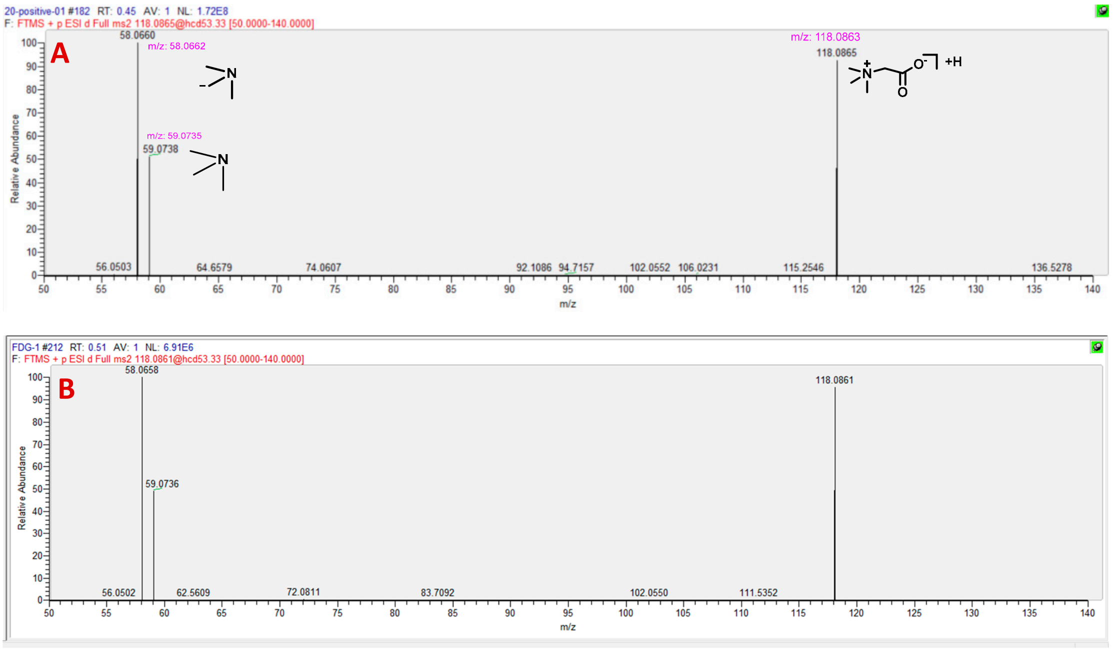

Figure S1. The main results of betaine (Cas. 107-43-7). (A) The MS/MS fragments of standard betaine with a retention time of 0.45 min. (B) The MS/MS spectra from chromatographic peak in the *Jatropha podagrica* fruits extract with a retention time of 0.51 min.

Note： The m/z values in purple are the calculated ones. The m/z calculation was based on the relative atomic masses of C (12.0000), H (1.007825), O (15.994915), and N (14.003074)[1]

Identification: As seen in Figure S1, the retention time, MS/MS spectra, and characteristic pears were highly similar. Thus, the chromatographic peaks in the *Jatropha podagrica* fruits extracts were identified as D-gluconic acid (Cas. 526-95-4).

Suppl. S2.2 Identification of quinic acid (Cas. 77-95-2, $C_7H_{12}O_6$, M.W. 192.167).

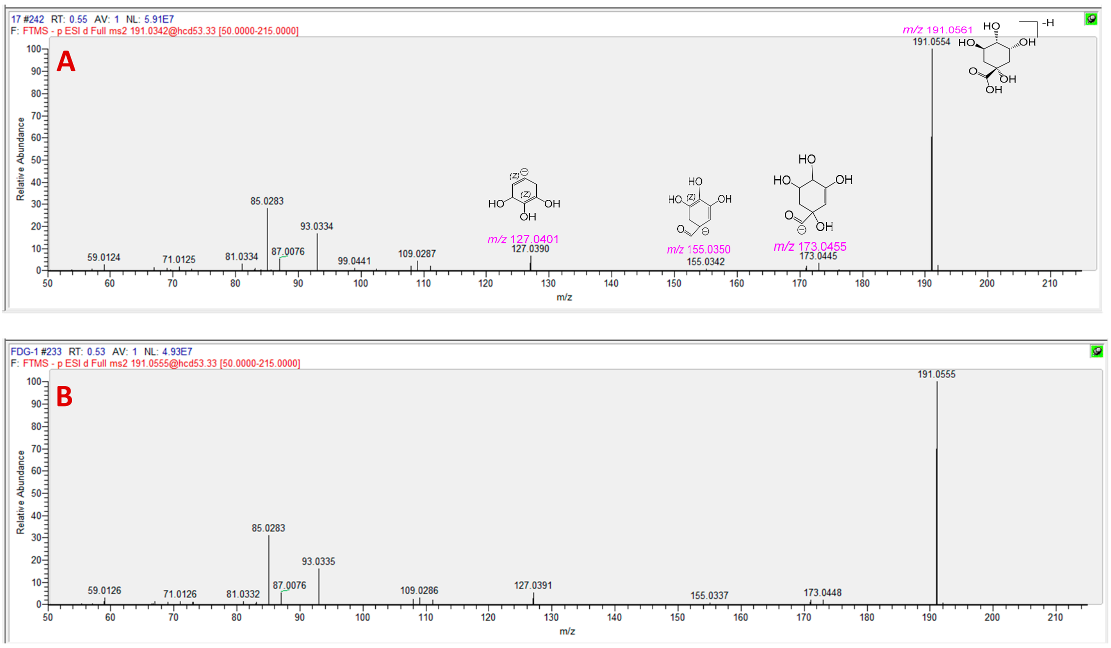

Figure S2. The main results of quinic acid (Cas. 77-95-2, $C_7H_{12}O_6$). (A) The MS/MS fragments of standard quinic acid with a retention time of 0.55 min. (B) The MS/MS spectra from chromatographic peak in the *Jatropha podagrica* fruits extract with a retention time of 0.53 min.

Note： The m/z values in purple are the calculated ones. The m/z calculation was based on the relative atomic masses of C (12.0000), H (1.007825), O (15.994915), and N (14.003074)[1]

Identification: As seen in Figure S2, the retention time, MS/MS spectra, and characteristic pears were highly similar. Thus, the chromatographic peaks in the *Jatropha podagrica* fruits extracts were identified as quinic acid (Cas. 77-95-2).

Suppl. S2.3 Identification of D-gluconic acid (Cas. 526-95-4, $C_6H_{11}O_7$, M.W. 195.1479).

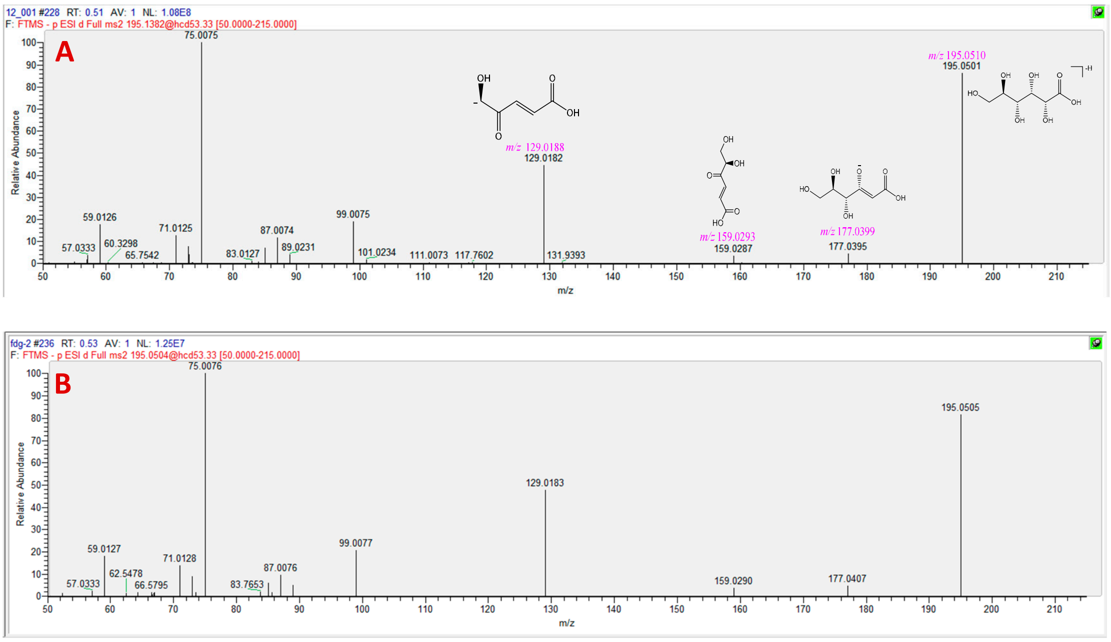

Figure S3. The main results of D-gluconic acid (Cas. 526-95-4, $C_6H_{11}O_7$). (A) The MS/MS fragments of standard D-gluconic acid with a retention time of 0.51 min. (B) The MS/MS spectra from chromatographic peak in the *Jatropha podagrica* fruits extract with a retention time of 0.53 min.

Note： The m/z values in purple are the calculated ones. The m/z calculation was based on the relative atomic masses of C (12.0000), H (1.007825), O (15.994915), and N (14.003074)[1]

Identification: As seen in Figure S3, the retention time, MS/MS spectra, and characteristic pears were highly similar. Thus, the chromatographic peaks in the *Jatropha podagrica* fruits extracts were identified as D-gluconic acid (Cas. 526-95-4).

Suppl. S2.4 Identification of malic acid (Cas. 6915-15-7, $C_4H_6O_5$, M.W.134.087).

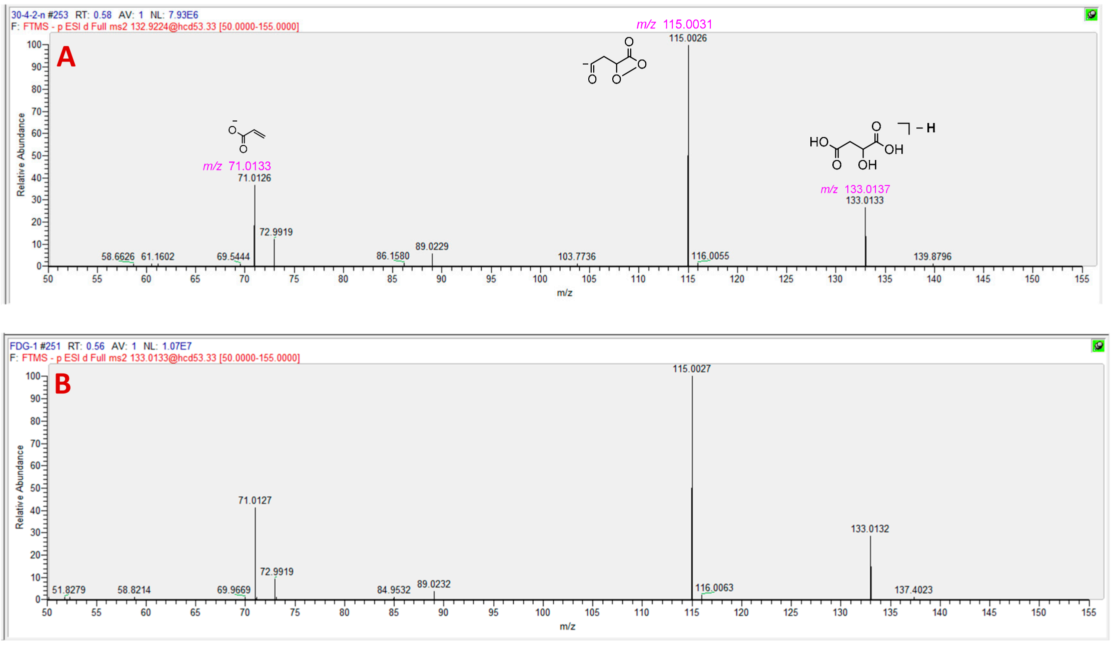

Figure S4. The main results of malic acid (Cas. 6915-15-7, $C_4H_6O_5$). (A) The MS/MS fragments of standard malic acid with a retention time of 0.58 min. (B) The MS/MS spectra from chromatographic peak in the *Jatropha podagrica* fruits extract with a retention time of 0.56 min.

Note：The m/z values in purple are the calculated ones. The m/z calculation was based on the relative atomic masses of C (12.0000), H (1.007825), O (15.994915), and N (14.003074)[1]

Identification: As seen in Figure S4, the retention time, MS/MS spectra, and characteristic pears were highly similar. Thus, the chromatographic peaks in the *Jatropha podagrica* fruits extracts were identified as malic acid (Cas. 6915-15-7).

Suppl. S2.5 Identification of sucrose (Cas. 57-50-1, $C_{12}H_{22}O_{11}$, M.W. 342.3).

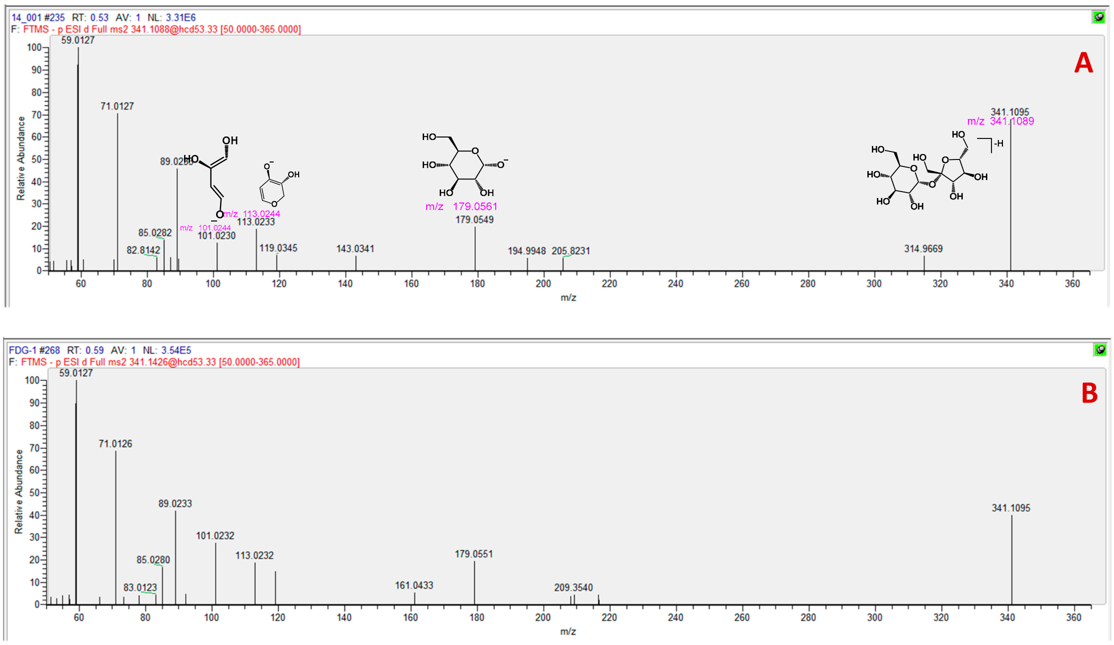

Figure S5. The main results of sucrose (Cas. 57-50-1, $C_{12}H_{22}O_{11}$). (A) The MS/MS fragments of standard sucrose with a retention time of 0.53 min. (B) The MS/MS spectra from chromatographic peak in the *Jatropha podagrica* fruits extract with a retention time of 0.59 min.

Note： The m/z values in purple are the calculated ones. The m/z calculation was based on the relative atomic masses of C (12.0000), H (1.007825), O (15.994915), and N (14.003074)[1]

Identification: As seen in Figure S5, the retention time, MS/MS spectra, and characteristic pears were highly similar. Thus, the chromatographic peaks in the *Jatropha podagrica* fruits extracts were identified as sucrose (Cas. 57-50-1).

Suppl. S2.6 Identification of pyroglutamic acid (Cas. 98-79-3, $C_5H_7NO_3$, M.W. 129.115).

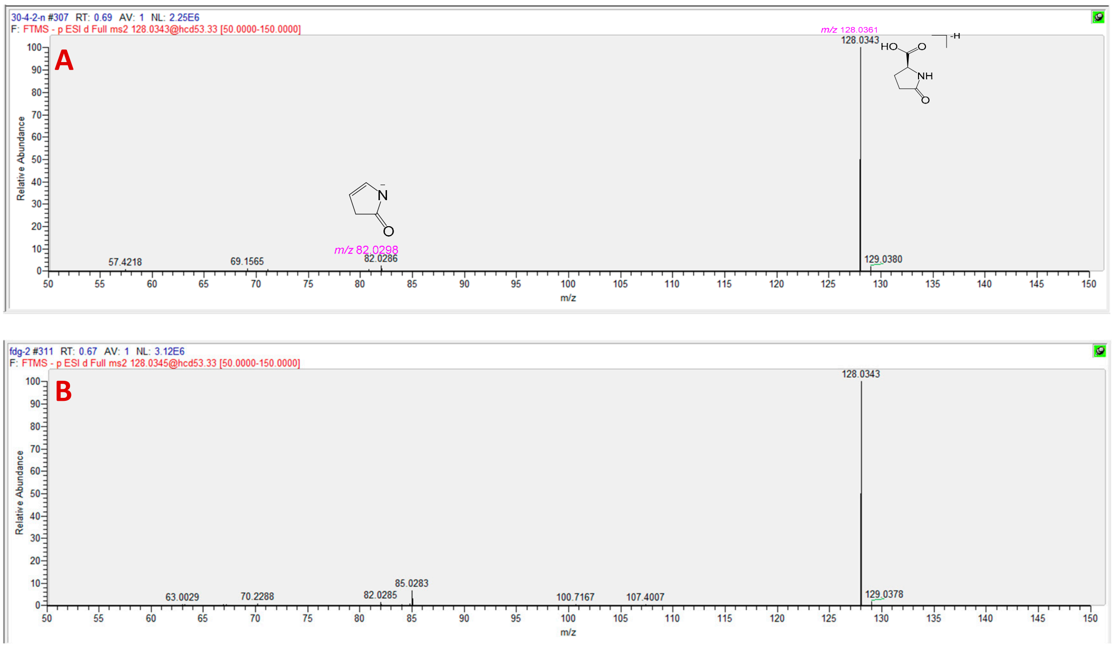

Figure S6. The main results of pyroglutamic acid (Cas. 98-79-3, $C_5H_7NO_3$). (A) The MS/MS fragments of standard pyroglutamic acid with a retention time of 0.69 min. (B) The MS/MS spectra from chromatographic peak in the *Jatropha podagrica* fruits extract with a retention time of 0.67 min.

Note：The m/z values in purple are the calculated ones. The m/z calculation was based on the relative atomic masses of C (12.0000), H (1.007825), O (15.994915), and N (14.003074)[1]

Identification: As seen in Figure S6, the retention time, MS/MS spectra, and characteristic pears were highly similar. Thus, the chromatographic peaks in the *Jatropha podagrica* fruits extracts were identified as pyroglutamic acid (Cas. 98-79-3).

Suppl. S2. 7 Identification of gallic acid (Cas. 149-91-7, $C_7H_6O_5$, M.W. 170.12).

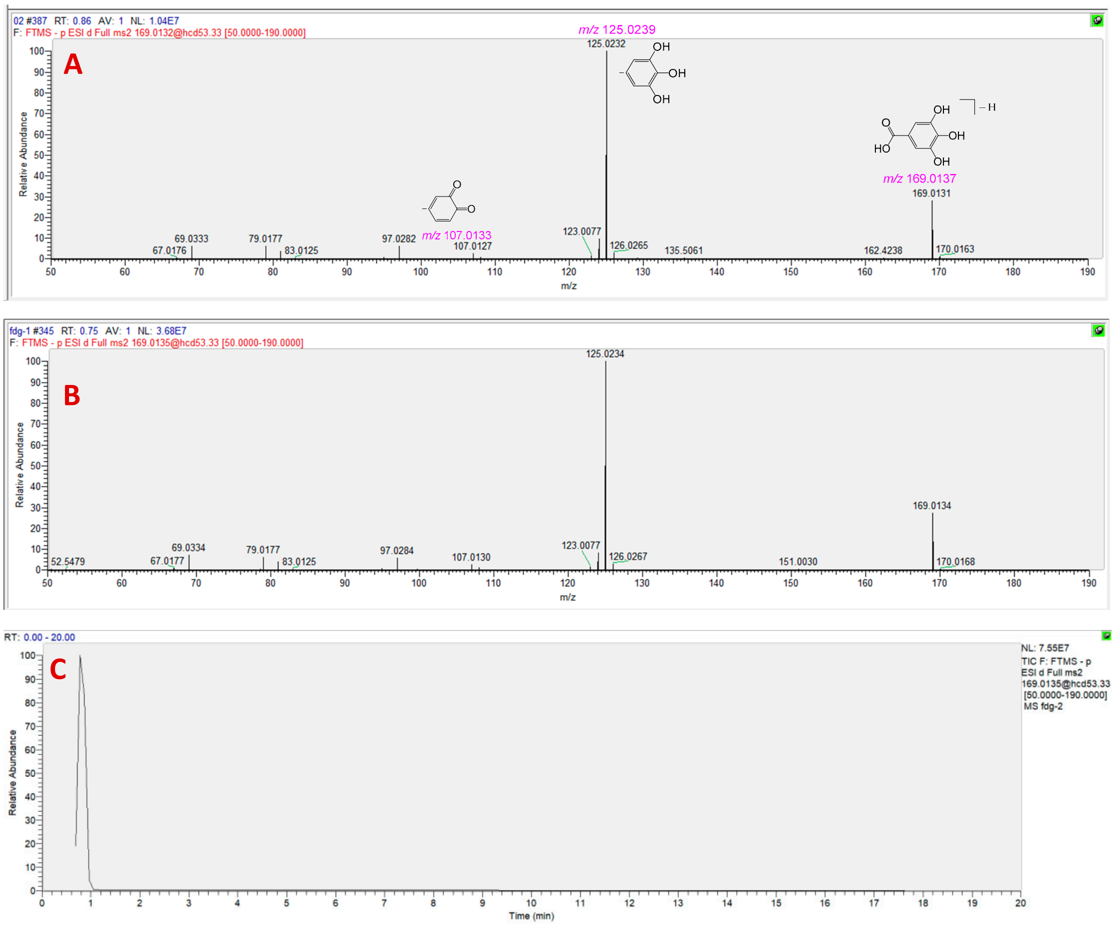

Figure S7. The main results of gallic acid (Cas. 149-91-7, $C_7H_6O_5$) and its corresponding peak in the TIC diagram using UPLC-Q-Orbitrap-MS analysis. (A) The MS/MS fragments of standard gallic acid with a retention time of 0.86 min. (B) The MS/MS spectra from chromatographic peak in the *Jatropha podagrica* fruits extract with a retention time of 0.75 min. (C) Extracted ion chromatogram of m/z 169.01 from the *Jatropha podagrica* fruits extract.

Note： The m/z values in purple are the calculated ones. The m/z calculation was based on the relative atomic masses of C (12.0000), H (1.007825), O (15.994915), and N (14.003074)[1]

Identification: As seen in Figure S7, the retention time, MS/MS spectra, and characteristic pears were highly similar. Thus, the chromatographic peaks in the *Jatropha podagrica* fruits extracts were identified as gallic acid (Cas. 149-91-7).

Suppl. S2.8 Identification of L-phenylalanine (Cas. 63-91-2, $C_9H_{11}NO_2$, M.W. 165).

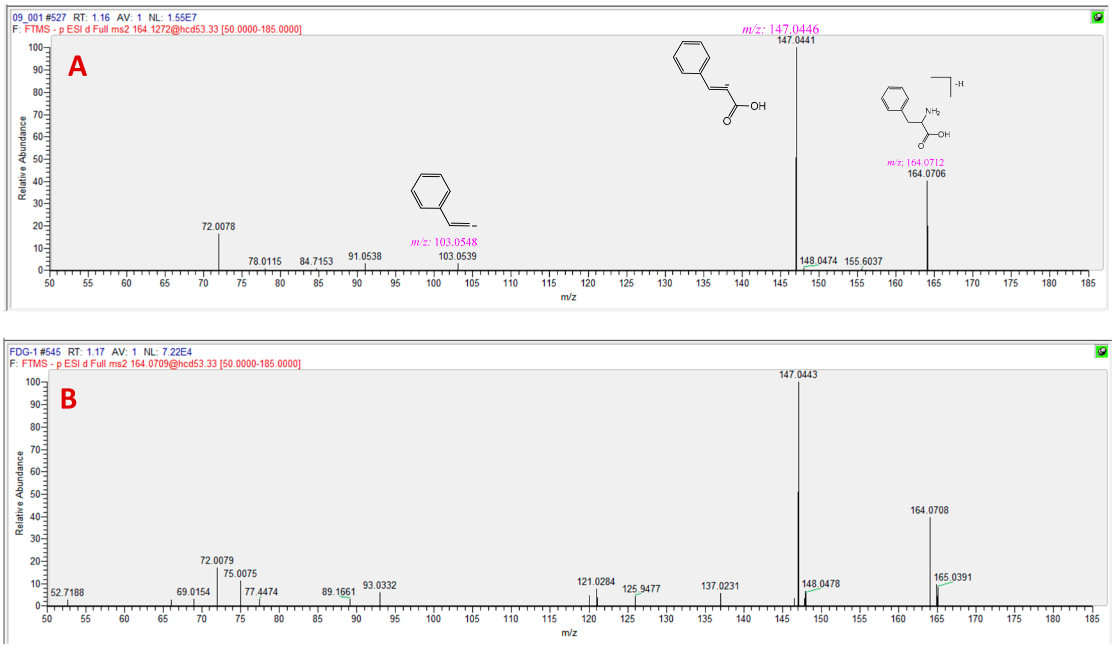

Figure S8. The main results of L-phenylalanine (Cas. 63-91-2, $C_9H_{11}NO_2$). (A) The MS/MS fragments of standard L-phenylalanine with a retention time of 1.16 min. (B) The MS/MS spectra from chromatographic peak in the *Jatropha podagrica* fruits extract with a retention time of 1.17 min.

Note: The m/z values in purple are the calculated ones. The m/z calculation was based on the relative atomic masses of C (12.0000), H (1.007825), O (15.994915), and N (14.003074)[1]

Identification: As seen in Figure S8, the retention time, MS/MS spectra, and characteristic pears were highly similar. Thus, the chromatographic peaks in the *Jatropha podagrica* fruits extracts were identified as L-phenylalanine (Cas. 63-91-2).

Suppl. S2.9 Identification of protocatechuic acid (Cas. 99-50-3, $C_7H_6O_4$, M.W. 154.12).

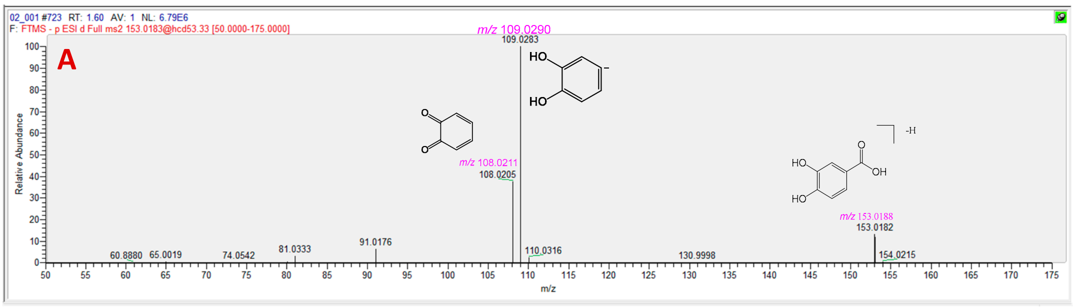

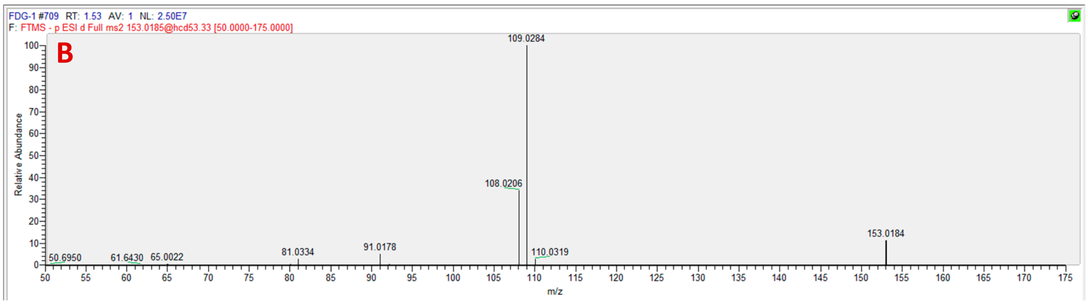

Figure S9. The main results of protocatechuic acid (Cas. 99-50-3, $C_7H_6O_4$). (A) The MS/MS fragments of standard protocatechuic acid with a retention time of 1.60 min. (B) The MS/MS spectra from chromatographic peak in the *Jatropha podagrica* fruits extract with a retention time of 1.53 min.

Note： The m/z values in purple are the calculated ones. The m/z calculation was based on the relative atomic masses of C (12.0000), H (1.007825), O (15.994915), and N (14.003074)[1]

Identification: As seen in Figure S9, the retention time, MS/MS spectra, and characteristic pears were highly similar. Thus, the chromatographic peaks in the *Jatropha podagrica* fruits extracts were identified as protocatechuic acid (Cas. 99-50-3).

Suppl. S2.10 Identification of 3,4-dihydroxybenzaldehyde (Cas. 139-85-5, $C_7H_6O_3$, M.W. 138.122).

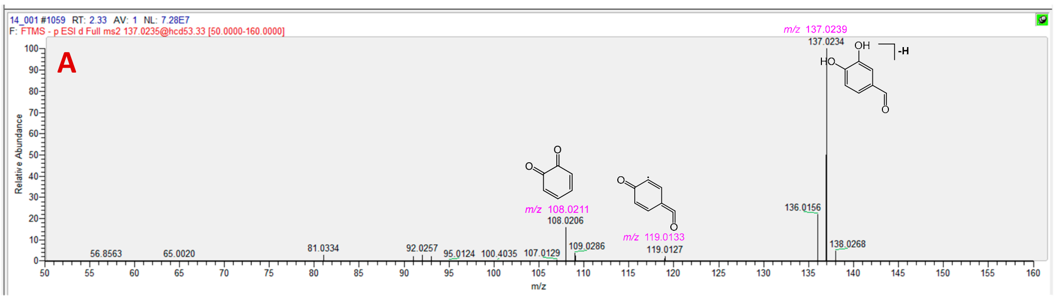

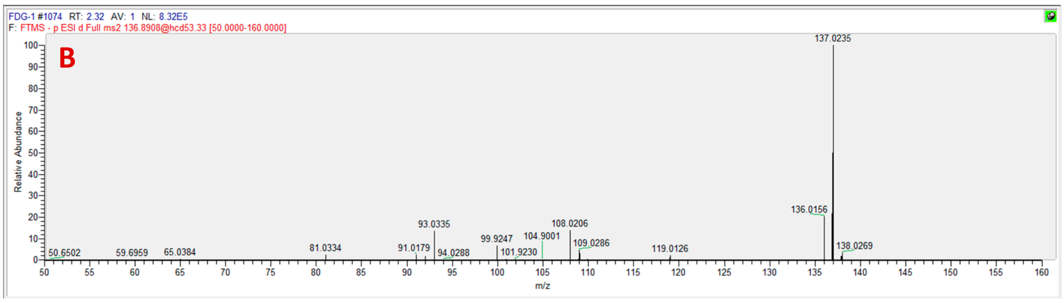

Figure S10. The main results of 3,4-dihydroxybenzaldehyde (Cas. 139-85-5, $C_7H_6O_3$). (A) The MS/MS fragments of standard 3,4-dihydroxybenzaldehyde with a retention time of 2.33 min. (B) The MS/MS spectra from chromatographic peak in the *Jatropha podagrica* fruits extract with a retention time of 2.32 min.

Note: The m/z values in purple are the calculated ones. The m/z calculation was based on the relative atomic masses of C (12.0000), H (1.007825), O (15.994915), and N (14.003074)[1]

Identification: As seen in Figure S10, the retention time, MS/MS spectra, and characteristic pears were highly similar. Thus, the chromatographic peaks in the *Jatropha podagrica* fruits extracts were identified as 3,4-dihydroxybenzaldehyde (Cas. 139-85-5).

Suppl. S2.11 Identification of (-)-catechin (Cas. 18829-70-4, $C_{15}H_{14}O_6$, M.W. 290.27).

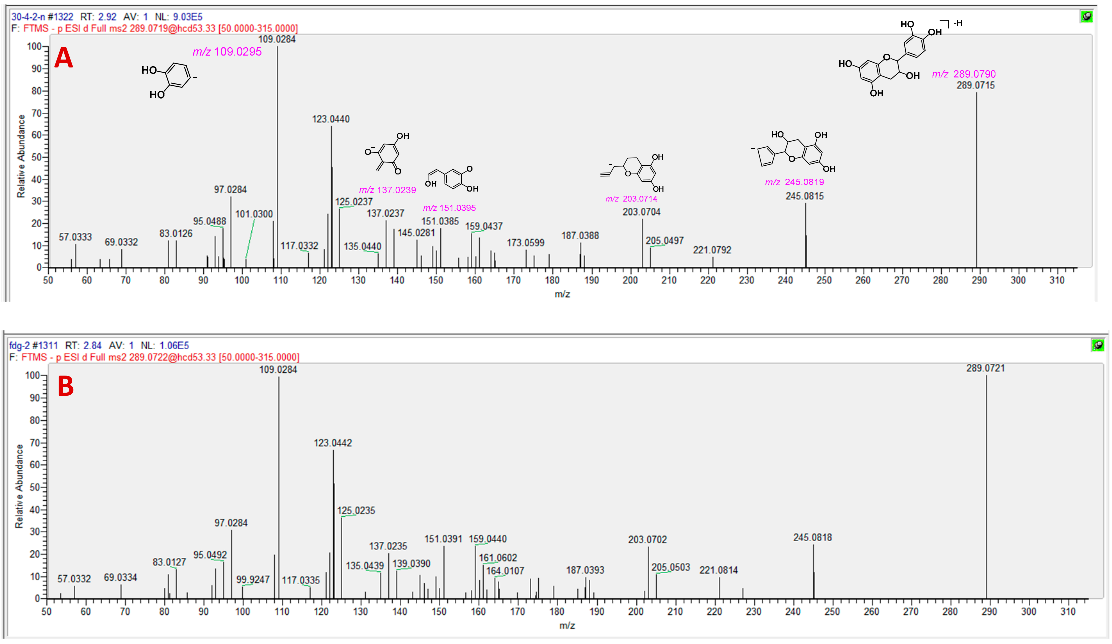

Figure S11. The main results of (-)-catechin (Cas. 18829-70-4, $C_{15}H_{14}O_6$). (A) The MS/MS fragments of standard (-)-catechin with a retention time of 2.92 min. (B) The MS/MS spectra from chromatographic peak in the *Jatropha podagrica* fruits extract with a retention time of 2.84 min.

Note: The m/z values in purple are the calculated ones. The m/z calculation was based on the relative atomic masses of C (12.0000), H (1.007825), O (15.994915), and N (14.003074)[1]

Identification: As seen in Figure S11, the retention time, MS/MS spectra, and characteristic pears were highly similar. Thus, the chromatographic peaks in the *Jatropha podagrica* fruits extracts were identified as (-)-catechin (Cas. 18829-70-4).

Suppl. S2.12 Identification of caffeine (Cas. 58-08-2, $C_8H_{10}N_4O_2$, M.W. 194.194).

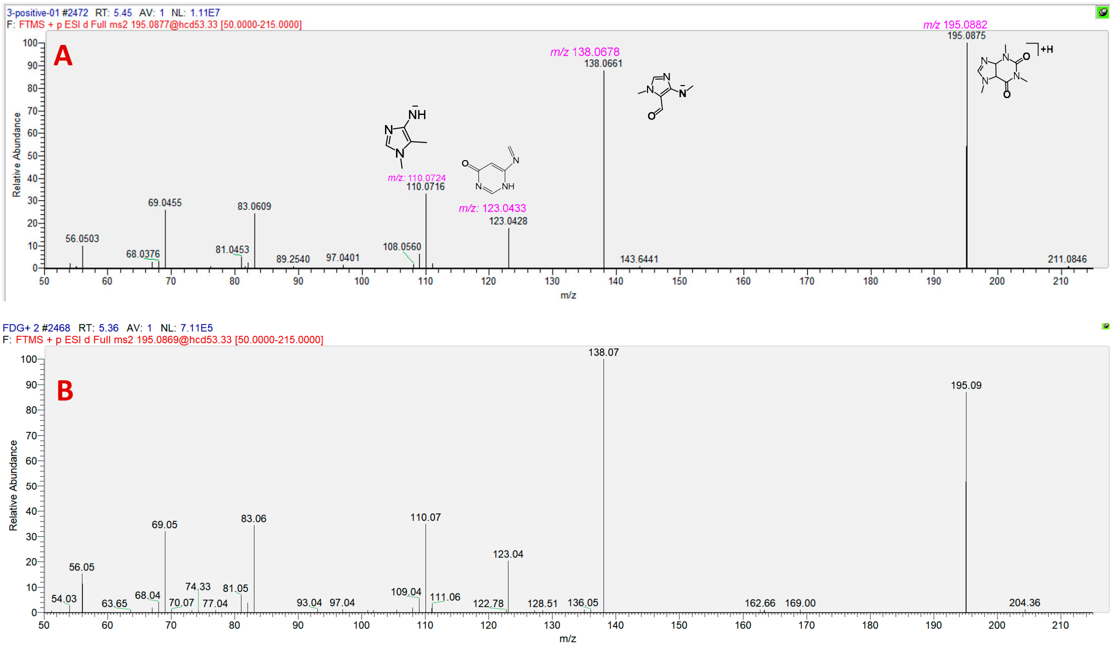

Figure S12. The main results of caffeine (Cas. 58-08-2, $C_8H_{10}N_4O_2$). (A) The MS/MS fragments of standard caffeine with a retention time of 5.45 min. (B) The MS/MS spectra from chromatographic peak in the *Jatropha podagrica* fruits extract with a retention time of 5.36 min.

Note： The m/z values in purple are the calculated ones. The m/z calculation was based on the relative atomic masses of C (12.0000), H (1.007825), O (15.994915), and N (14.003074)[1]

Identification: As seen in Figure S12, the retention time, MS/MS spectra, and characteristic pears were highly similar. Thus, the chromatographic peaks in the *Jatropha podagrica* fruits extracts were identified as caffeine (Cas. 58-08-2).

Suppl. S2.13 Identification of fraxin (Cas. 524-30-1, $C_{16}H_{18}O_{10}$, M.W.370.31).

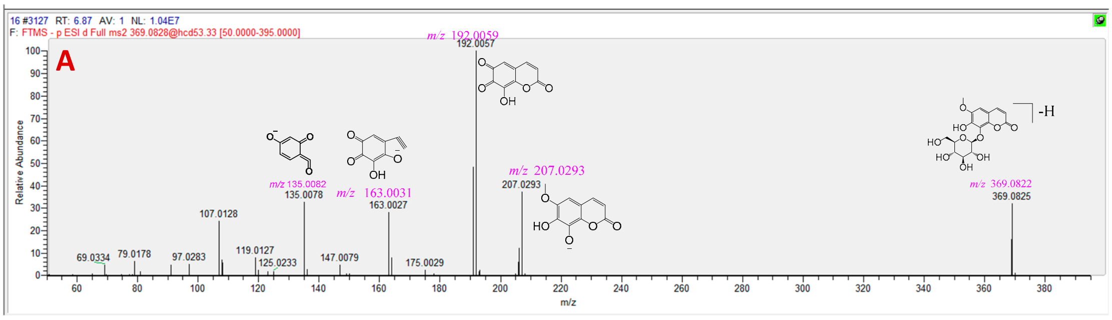

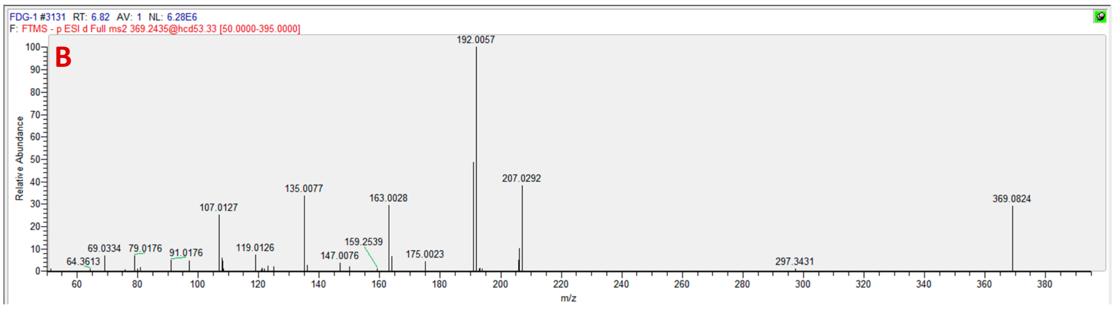

Figure S13. The main results of fraxin (Cas. 524-30-1, $C_{16}H_{18}O_{10}$). (A) The MS/MS fragments of standard fraxin with a retention time of 6.87 min. (B) The MS/MS spectra from chromatographic peak in the *Jatropha podagrica* fruits extract with a retention time of 6.82 min.

Note: The m/z values in purple are the calculated ones. The m/z calculation was based on the relative atomic masses of C (12.0000), H (1.007825), O (15.994915), and N (14.003074)[1]

Identification: As seen in Figure S13, the retention time, MS/MS spectra, and characteristic pears were highly similar. Thus, the chromatographic peaks in the *Jatropha podagrica* fruits extracts were identified as fraxin (Cas. 524-30-1).

Suppl. S2.14 Identification of (-)-epicatechin (Cas. 490-46-0, $C_{15}H_{14}O_6$, M.W. 290.27).

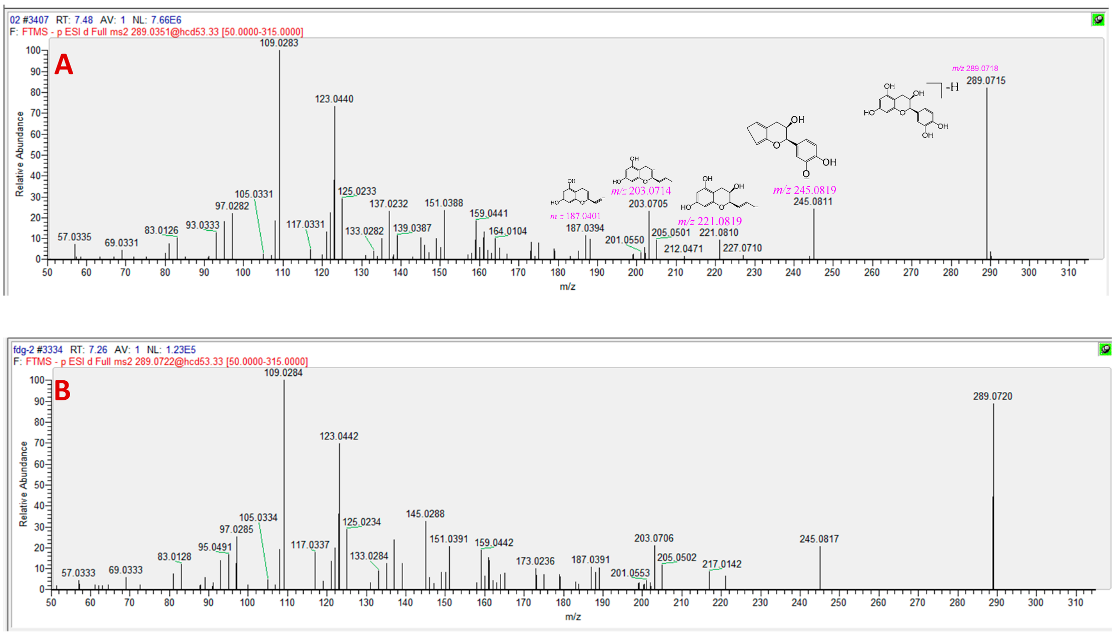

Figure S14. The main results of (-)-epicatechin (Cas. 490-46-0, $C_{15}H_{14}O_6$). (A) The MS/MS fragments of standard (-)-epicatechin with a retention time of 7.48 min. (B) The MS/MS spectra from chromatographic peak in the *Jatropha podagrica* fruits extract with a retention time of 7.26 min.

Note: The m/z values in purple are the calculated ones. The m/z calculation was based on the relative atomic masses of C (12.0000), H (1.007825), O (15.994915), and N (14.003074)[1]

Identification: As seen in Figure S14, the retention time, MS/MS spectra, and characteristic pears were highly similar. Thus, the chromatographic peaks in the *Jatropha podagrica* fruits extracts were identified as (-)-epicatechin (Cas. 490-46-0).

Suppl. S2.15 Identification of fraxetin (Cas.574-84-5, $C_{10}H_8O_5$, M.W. 208.17).

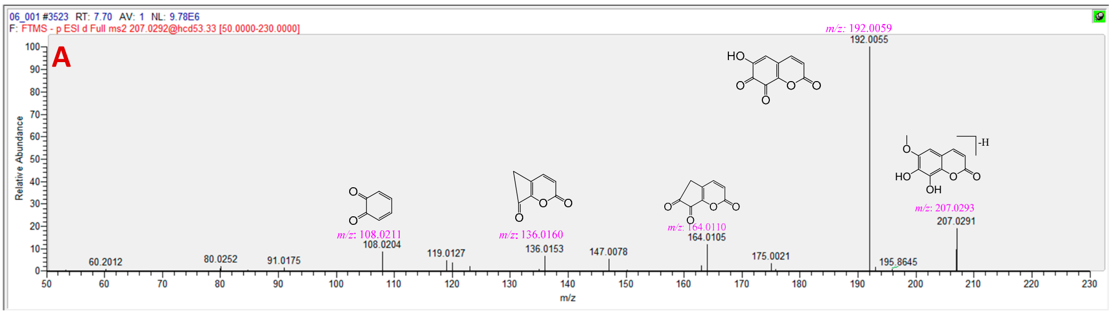

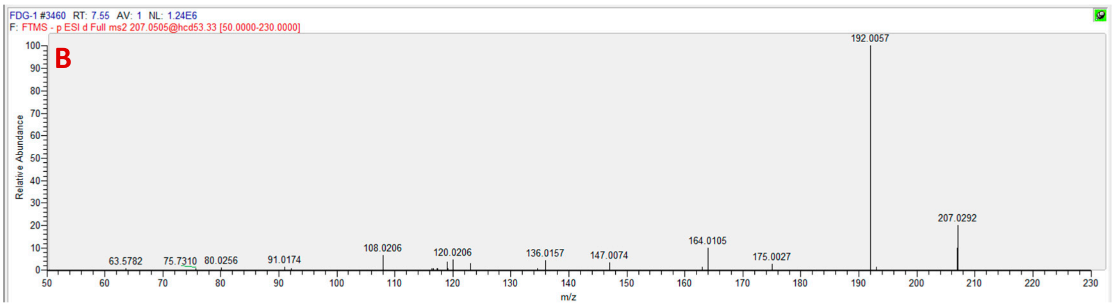

Figure S15. The main results of fraxetin (Cas. 574-84-5, $C_{10}H_8O_5$). (A) The MS/MS fragments of standard fraxetin with a retention time of 7.70 min. (B) The MS/MS spectra from chromatographic peak in the *Jatropha podagrica* fruits extract with a retention time of 7.55 min.

Note: The m/z values in purple are the calculated ones. The m/z calculation was based on the relative atomic masses of C (12.0000), H (1.007825), O (15.994915), and N (14.003074)[1]

Identification: As seen in Figure S15, the retention time, MS/MS spectra, and characteristic pears were highly similar. Thus, the chromatographic peaks in the *Jatropha podagrica* fruits extracts were identified as fraxetin (Cas. 574-84-5).

Suppl. S2.16 Identification of corilagin (Cas. 23094-69-1, $C_{27}H_{22}O_{18}$, M.W. 634.45).

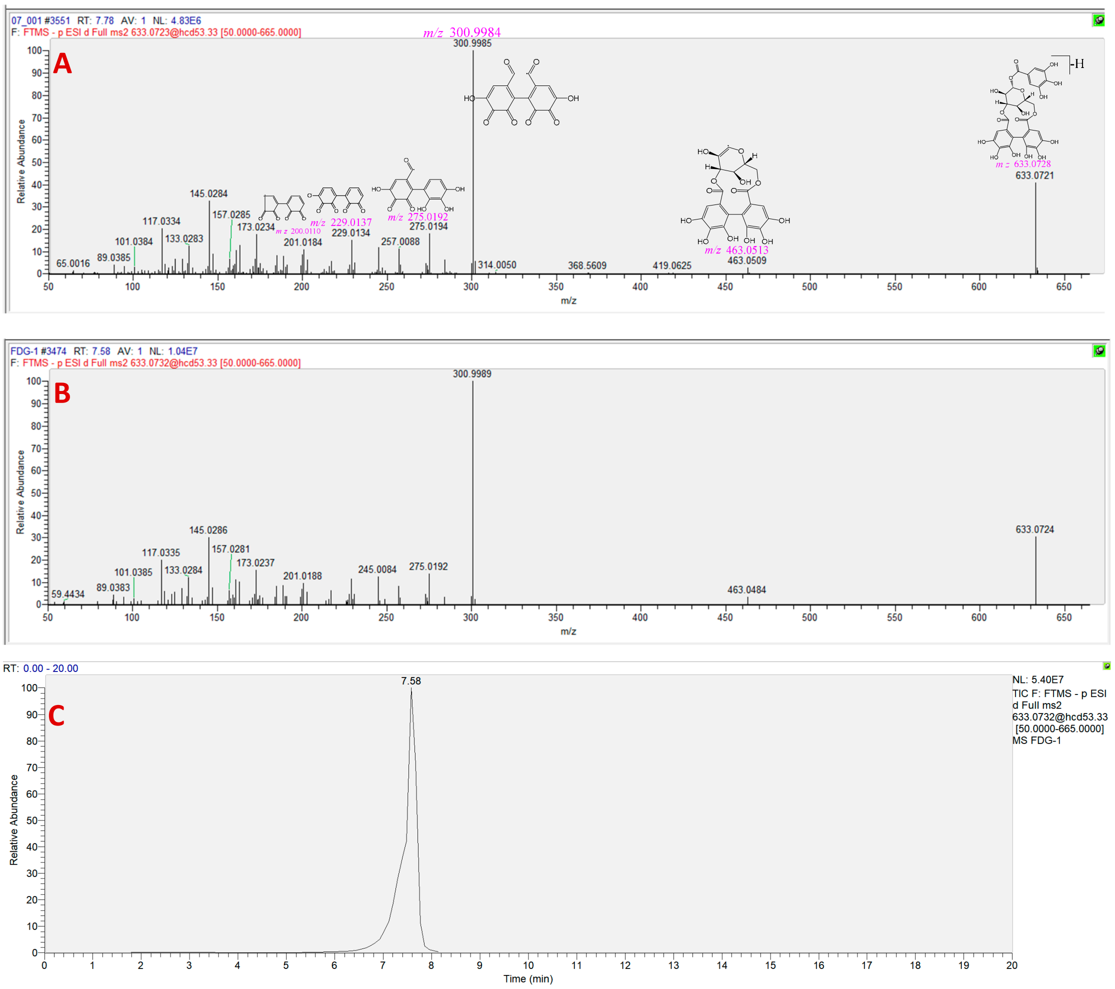

Figure S16. The main results of corilagin (Cas. 23094-69-1, $C_{27}H_{22}O_{18}$) and its corresponding peak in the TIC diagram using UPLC-Q-Orbitrap-MS analysis. (A) The MS/MS fragments of standard corilagin with a retention time of 7.78 min. (B) The MS/MS spectra from chromatographic peak in the *Jatropha podagrica* fruits extract with a retention time of 7.58 min. (C) Extracted ion chromatogram of m/z 633.07 from the *Jatropha podagrica* fruits extract.

Note：The m/z values in purple are the calculated ones. The m/z calculation was based on the relative atomic masses of C (12.0000), H (1.007825), O (15.994915), and N (14.003074)[1]

Identification: As seen in Figure S16, the retention time, MS/MS spectra, and characteristic pears were highly similar. Thus, the chromatographic peaks in the *Jatropha podagrica* fruits extracts were identified as corilagin (Cas. 23094-69-1).

Suppl. S2.17 Identification of ethyl gallate (Cas. 831-61-8, $C_9H_{10}O_5$, M.W. 198.17).

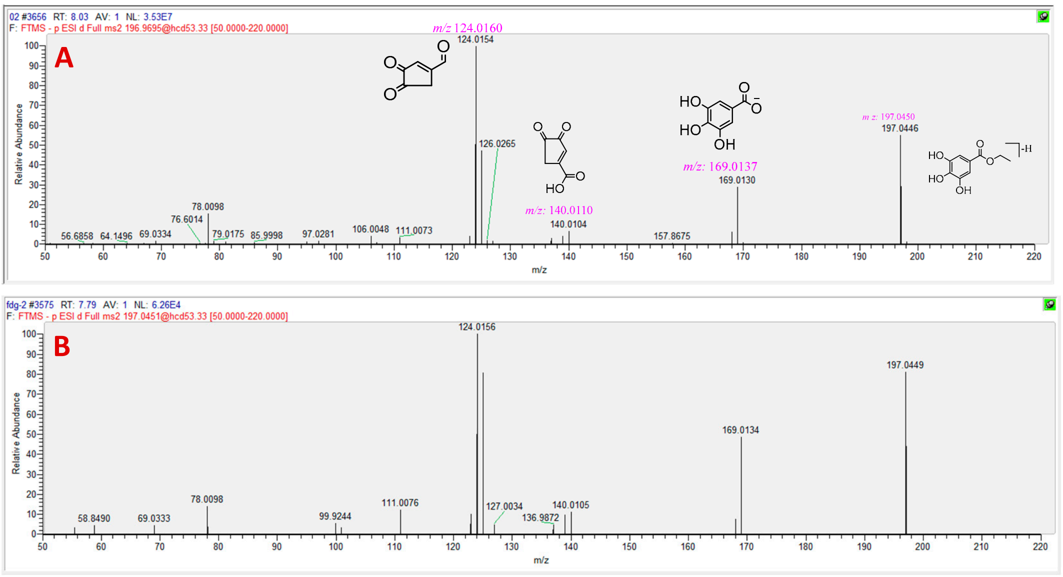

Figure S17. The main results of ethyl gallate (Cas. 831-61-8, $C_9H_{10}O_5$). (A) The MS/MS fragments of standard ethyl gallate with a retention time of 8.03 min. (B) The MS/MS spectra from chromatographic peak in the *Jatropha podagrica* fruits extract with a retention time of 7.79 min.

Note： The m/z values in purple are the calculated ones. The m/z calculation was based on the relative atomic masses of C (12.0000), H (1.007825), O (15.994915), and N (14.003074)[1]

Identification: As seen in Figure S17, the retention time, MS/MS spectra, and characteristic pears were highly similar. Thus, the chromatographic peaks in the *Jatropha podagrica* fruits extracts were identified as ethyl gallate (Cas. 831-61-8).

Suppl. S2.18 Identification of scopoletin (Cas. 92-61-5, $C_{10}H_8O_4$, M.W. 192.17).

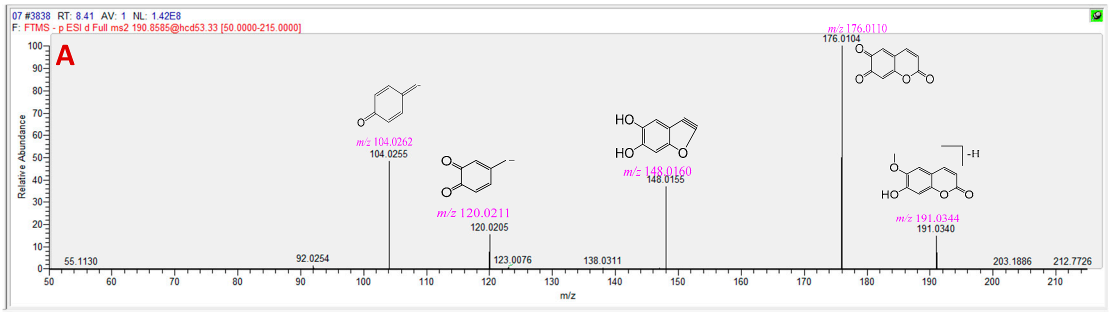

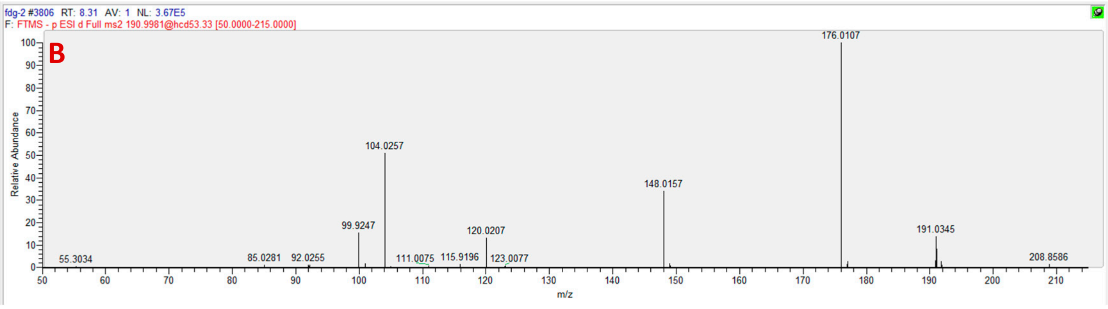

Figure S18. The main results of scopoletin (Cas. 92-61-5, $C_{10}H_8O_4$). (A) The MS/MS fragments of standard scopoletin with a retention time of 8.41 min. (B) The MS/MS spectra from chromatographic peak in the *Jatropha podagrica* fruits extract with a retention time of 8.31 min.

Note： The m/z values in purple are the calculated ones. The m/z calculation was based on the relative atomic masses of C (12.0000), H (1.007825), O (15.994915), and N (14.003074)[1]

Identification: As seen in Figure S18, the retention time, MS/MS spectra, and characteristic pears were highly similar. Thus, the chromatographic peaks in the *Jatropha podagrica* fruits extracts were identified as scopoletin (Cas. 92-61-5).

Suppl. S2.19 Identification of ferulic acid (Cas. 1135-24-6, $C_{10}H_{10}O_4$, M.W. 194.19).

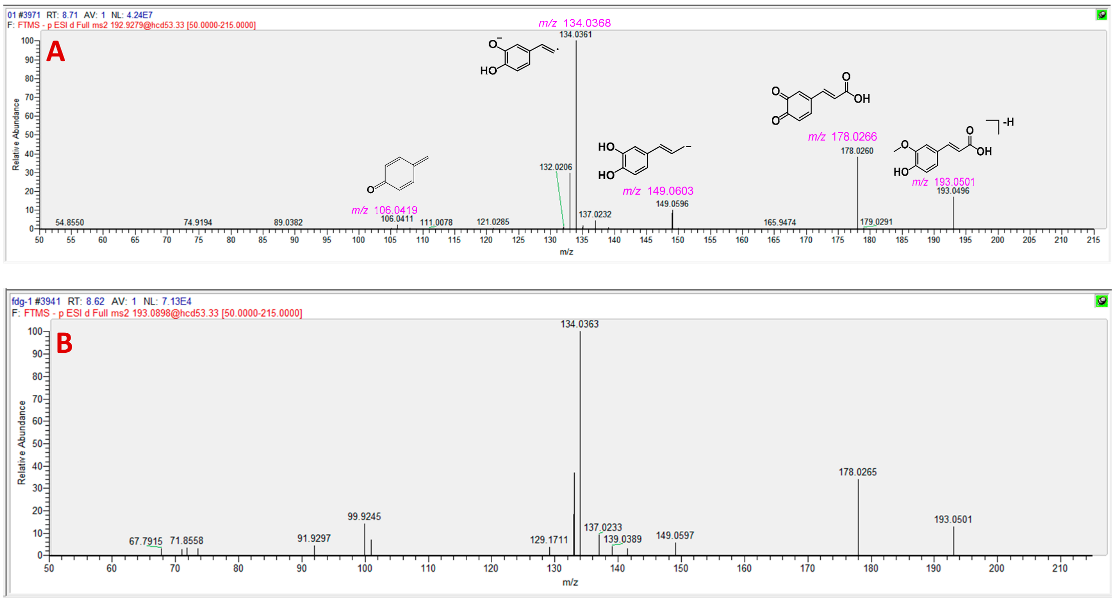

Figure S19. The main results of ferulic acid (Cas. 1135-24-6, $C_{10}H_{10}O_4$). (A) The MS/MS fragments of standard ferulic acid with a retention time of 8.71 min. (B) The MS/MS spectra from chromatographic peak in the *Jatropha podagrica* fruits extract with a retention time of 8.62 min.

Note: The m/z values in purple are the calculated ones. The m/z calculation was based on the relative atomic masses of C (12.0000), H (1.007825), O (15.994915), and N (14.003074)[1]

Identification: As seen in Figure S19, the retention time, MS/MS spectra, and characteristic pears were highly similar. Thus, the chromatographic peaks in the *Jatropha podagrica* fruits extracts were identified as ferulic acid (Cas. 1135-24-6).

Suppl. S2.20 Identification of orientin (Cas. 28608-75-5, $C_{21}H_{20}O_{11}$, M.W. 448.38).

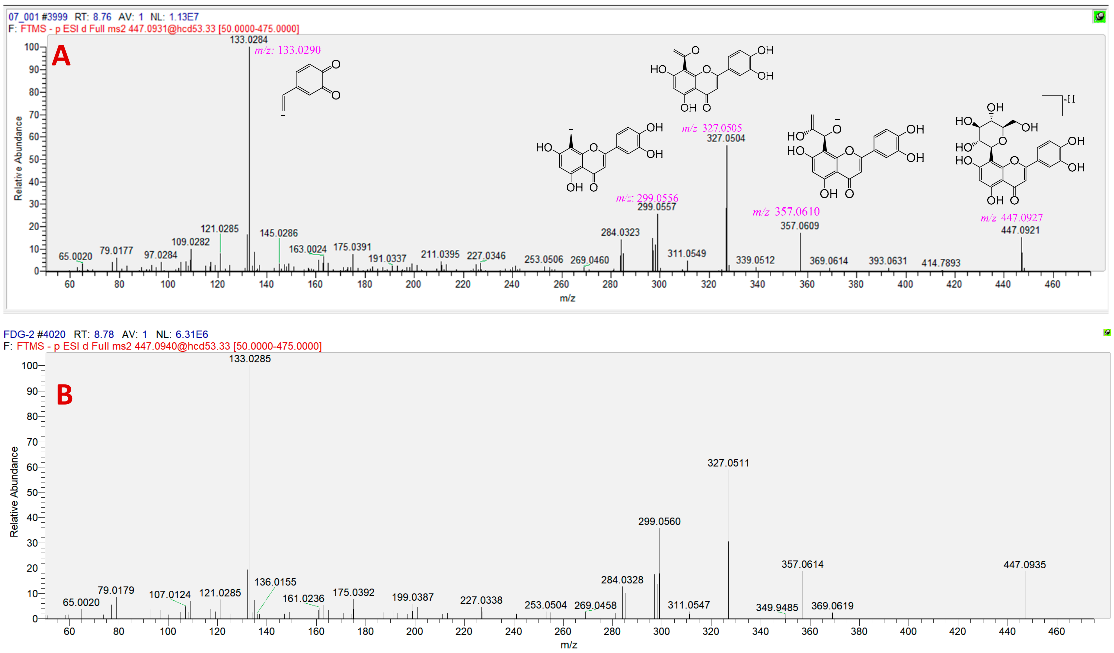

Figure S20. The main results of orientin (Cas. 28608-75-5, $C_{21}H_{20}O_{11}$). (A) The MS/MS fragments of standard orientin with a retention time of 8.76 min. (B) The MS/MS spectra from chromatographic peak in the *Jatropha podagrica* fruits extract with a retention time of 8.78 min.

Note: The m/z values in purple are the calculated ones. The m/z calculation was based on the relative atomic masses of C (12.0000), H (1.007825), O (15.994915), and N (14.003074)[1]

Identification: As seen in Figure S20, the retention time, MS/MS spectra, and characteristic pears were highly similar. Thus, the chromatographic peaks in the *Jatropha podagrica* fruits extracts were identified as orientin (Cas. 28608-75-5).

Suppl. S2.21 Identification of schaftoside (Cas. 51938-32-0, $C_{26}H_{28}O_{14}$, M.W. 564.5).

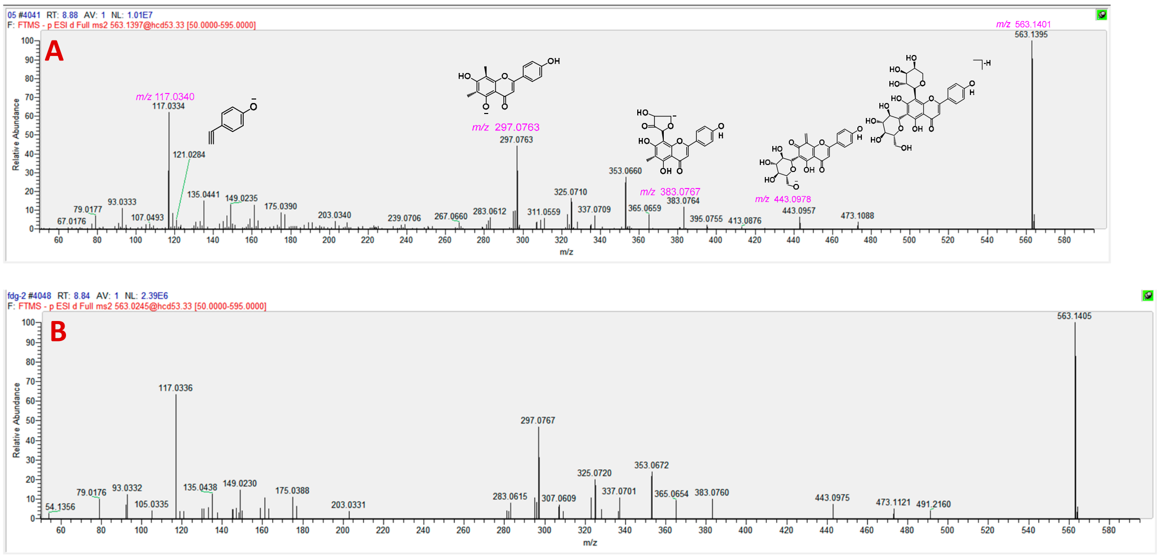

Figure S21. The main results of schaftoside (Cas. 51938-32-0, $C_{26}H_{28}O_{14}$). (A) The MS/MS fragments of standard schaftoside with a retention time of 8.88 min. (B) The MS/MS spectra from chromatographic peak in the *Jatropha podagrica* fruits extract with a retention time of 8.84 min.

Note： The m/z values in purple are the calculated ones. The m/z calculation was based on the relative atomic masses of C (12.0000), H (1.007825), O (15.994915), and N (14.003074)[1]

Identification: As seen in Figure S21, the retention time, MS/MS spectra, and characteristic pears were highly similar. Thus, the chromatographic peaks in the *Jatropha podagrica* fruits extracts were identified as schaftoside (Cas. 51938-32-0).

Suppl. S2.22 Identification of isoorientin (Cas. 4261-42-1, $C_{21}H_{20}O_{11}$, M.W. 448.38).

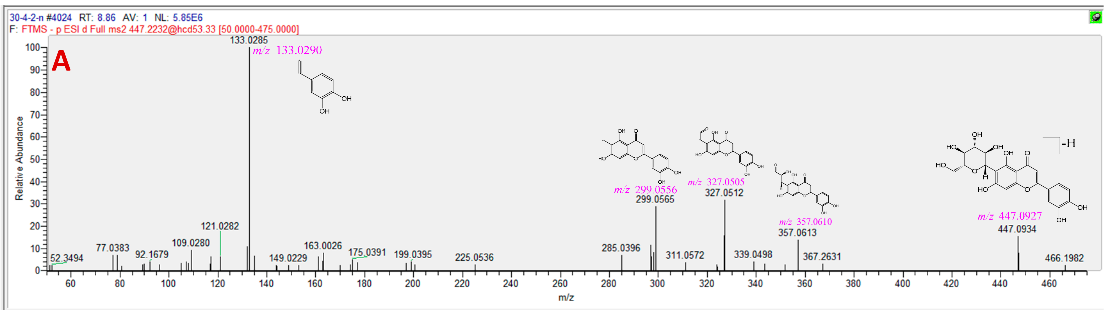

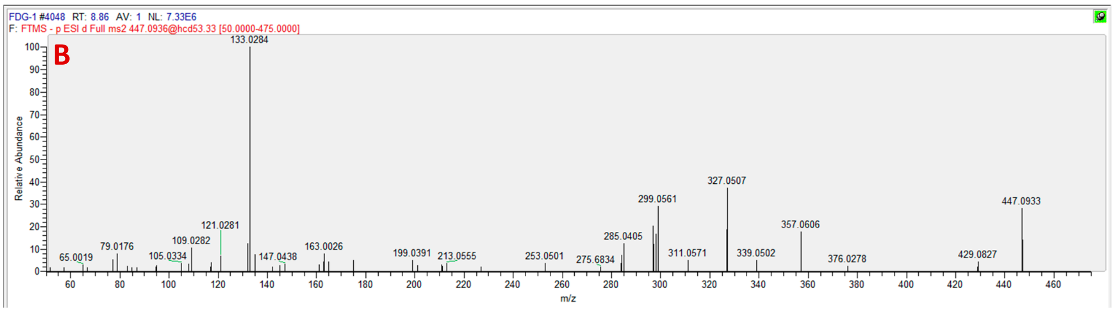

Figure S22. The main results of isoorientin (Cas. 4261-42-1, $C_{21}H_{20}O_{11}$). (A) The MS/MS fragments of standard isoorientin with a retention time of 8.86 min. (B) The MS/MS spectra from chromatographic peak in the *Jatropha podagrica* fruits extract with a retention time of 8.86 min.

Note： The m/z values in purple are the calculated ones. The m/z calculation was based on the relative atomic masses of C (12.0000), H (1.007825), O (15.994915), and N (14.003074)[1]

Identification: As seen in Figure S22, the retention time, MS/MS spectra, and characteristic pears were highly similar. Thus, the chromatographic peaks in the *Jatropha podagrica* fruits extracts were identified as isoorientin (Cas. 4261-42-1).

Suppl. S2.23 Identification of coniferaldehyde (Cas. 20649-42-7, $C_{10}H_{10}O_3$, M.W. 178.18).

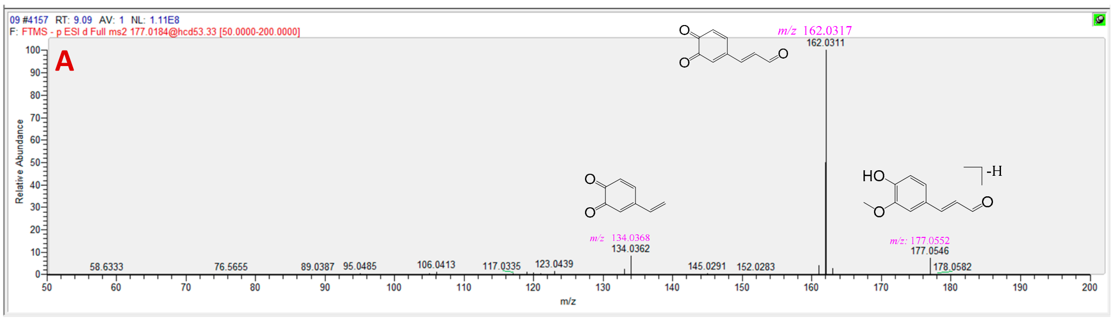

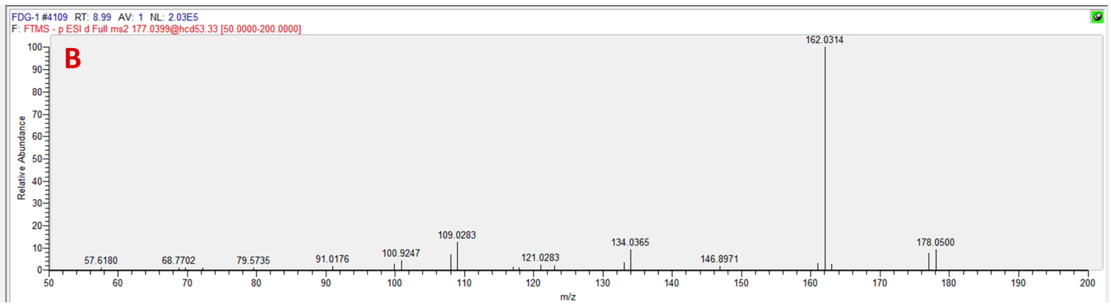

Figure S23. The main results of coniferaldehyde (Cas. 20649-42-7, $C_{10}H_{10}O_3$). (A) The MS/MS fragments of standard coniferaldehyde with a retention time of 9.09 min. (B) The MS/MS spectra from chromatographic peak in the *Jatropha podagrica* fruits extract with a retention time of 8.99 min.

Note: The m/z values in purple are the calculated ones. The m/z calculation was based on the relative atomic masses of C (12.0000), H (1.007825), O (15.994915), and N (14.003074)[1]

Identification: As seen in Figure S23, the retention time, MS/MS spectra, and characteristic pears were highly similar. Thus, the chromatographic peaks in the *Jatropha podagrica* fruits extracts were identified as coniferaldehyde (Cas. 20649-42-7).

Suppl. S2.24 Identification of isoschaftoside (Cas. 52012-29-0, $C_{26}H_{28}O_{14}$, M.W. 564.49).

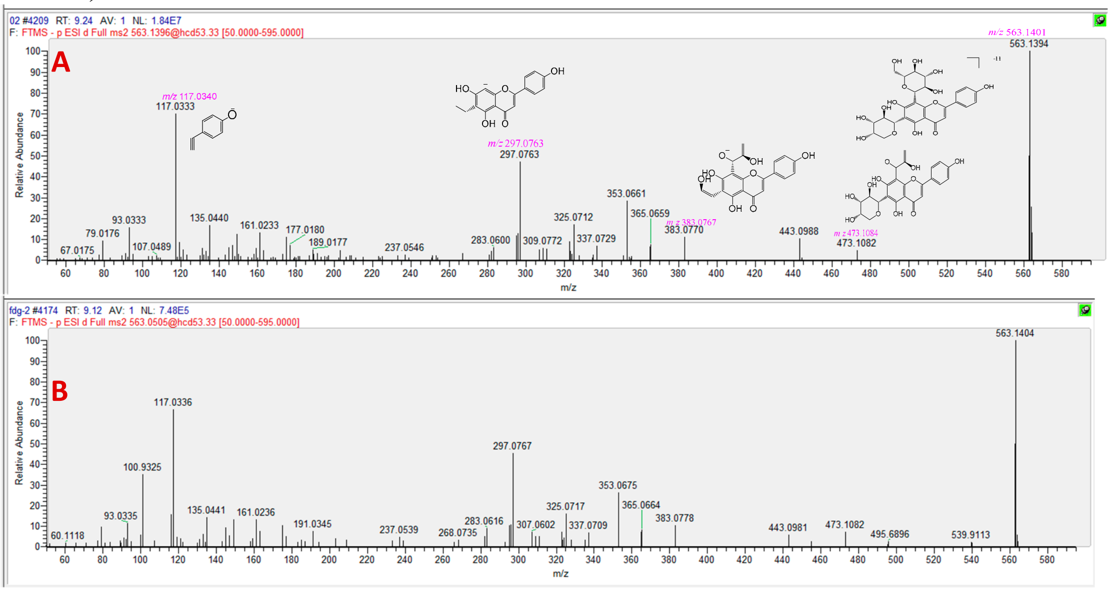

Figure S24. The main results of isoschaftoside (Cas. 52012-29-0, $C_{26}H_{28}O_{14}$). (A) The MS/MS fragments of standard isoschaftoside with a retention time of 9.24 min. (B) The MS/MS spectra from chromatographic peak in the *Jatropha podagrica* fruits extract with a retention time of 9.12 min.

Note: The m/z values in purple are the calculated ones. The m/z calculation was based on the relative atomic masses of C (12.0000), H (1.007825), O (15.994915), and N (14.003074)[1]

Identification: As seen in Figure S24, the retention time, MS/MS spectra, and characteristic pears were highly similar. Thus, the chromatographic peaks in the *Jatropha podagrica* fruits extracts were identified as isoschaftoside (Cas. 52012-29-0).

Suppl. S2.25 Identification of vitexin (Cas. 3681-93-4, $C_{21}H_{10}O_{10}$, M.W. 432.11).

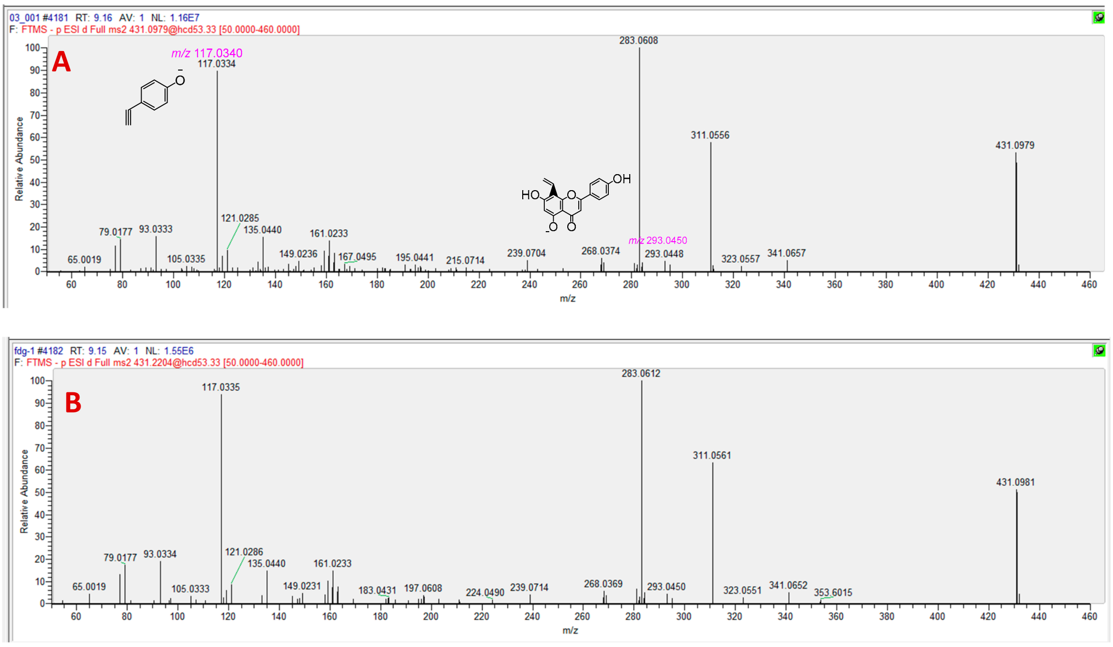

Figure S25. The main results of vitexin (Cas. 3681-93-4, $C_{21}H_{10}O_{10}$). (A) The MS/MS fragments of standard vitexin with a retention time of 9.16 min. (B) The MS/MS spectra from chromatographic peak in the *Jatropha podagrica* fruits extract with a retention time of 9.15 min.

Note: The m/z values in purple are the calculated ones. The m/z calculation was based on the relative atomic masses of C (12.0000), H (1.007825), O (15.994915), and N (14.003074)[1]

Identification: As seen in Figure S25, the retention time, MS/MS spectra, and characteristic pears were highly similar. Thus, the chromatographic peaks in the *Jatropha podagrica* fruits extracts were identified as vitexin (Cas. 3681-93-4).

Suppl. S2.26 Identification of neoeriocitrin (Cas. 13241-32-2, $C_{27}H_{32}O_{15}$, M.W. 596.538).

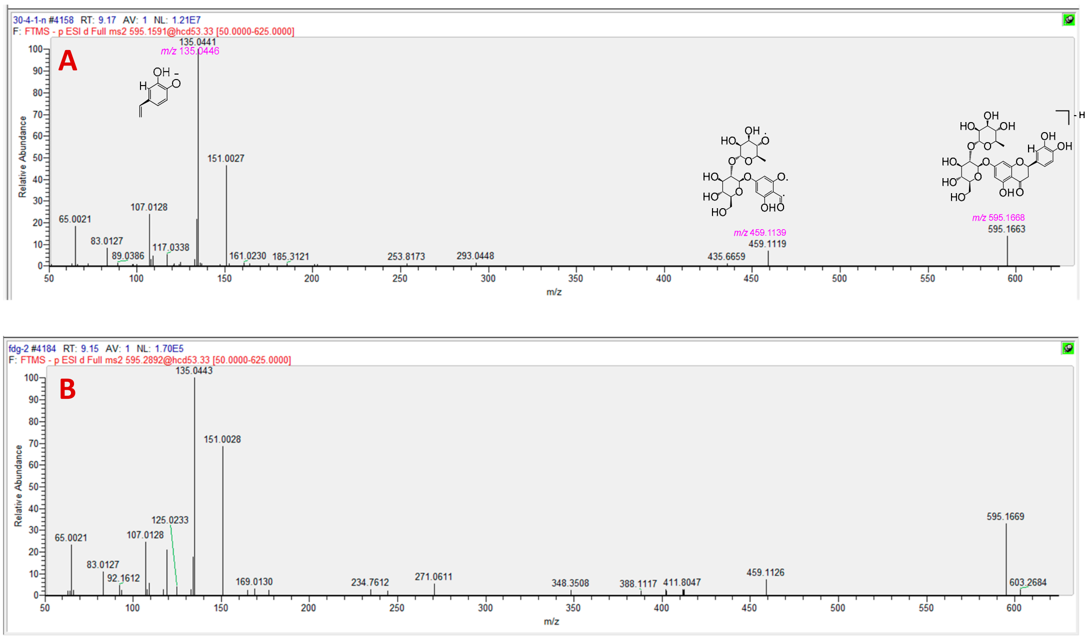

Figure S26. The main results of neoeriocitrin (Cas. 13241-32-2, $C_{27}H_{32}O_{15}$). (A) The MS/MS fragments of standard neoeriocitrin with a retention time of 9.17 min. (B) The MS/MS spectra from chromatographic peak in the *Jatropha podagrica* fruits extract with a retention time of 9.15 min.

Note： The m/z values in purple are the calculated ones. The m/z calculation was based on the relative atomic masses of C (12.0000), H (1.007825), O (15.994915), and N (14.003074)[1]

Identification: As seen in Figure S26, the retention time, MS/MS spectra, and characteristic pears were highly similar. Thus, the chromatographic peaks in the *Jatropha podagrica* fruits extracts were identified as neoeriocitrin (Cas. 13241-32-2).

Suppl. S2.27 Identification of indole-3-acetic acid (Cas. 87-51-4, $C_{10}H_9NO_2$, M.W. 175.187).

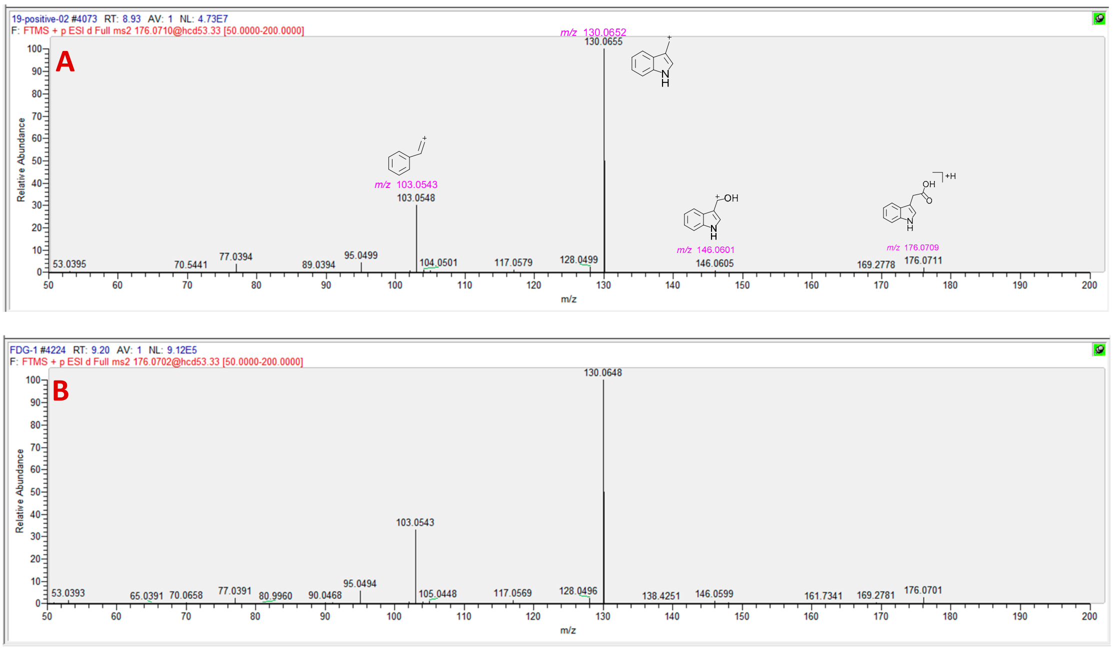

Figure S27. The main results of indole-3-acetic acid (Cas. 87-51-4,$C_{10}H_9NO_2$). (A) The MS/MS fragments of standard indole-3-acetic acid with a retention time of 8.93 min. (B) The MS/MS spectra from chromatographic peak in the *Jatropha podagrica* fruits extract with a retention time of 9.20 min.

Note: The m/z values in purple are the calculated ones. The m/z calculation was based on the relative atomic masses of C (12.0000), H (1.007825), O (15.994915), and N (14.003074)[1]

Identification: As seen in Figure S27, the retention time, MS/MS spectra, and characteristic pears were highly similar. Thus, the chromatographic peaks in the *Jatropha podagrica* fruits extracts were identified as indole-3-acetic acid (Cas. 87-51-4).

Suppl. S2.28 Identification of isovitexin (Cas. 38953-85-4, $C_{21}H_{20}O_{10}$, M.W. 432.3775).

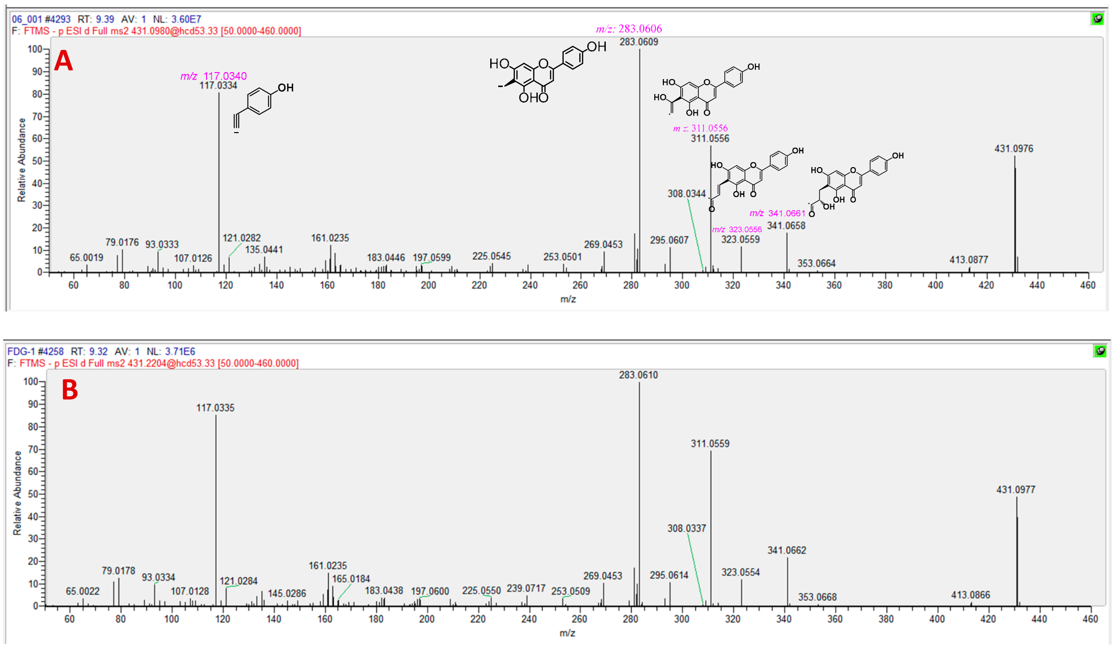

Figure S28. The main results of isovitexin (Cas. 38953-85-4, $C_{21}H_{20}O_{10}$). (A) The MS/MS fragments of standard isovitexin with a retention time of 9.39 min. (B) The MS/MS spectra from chromatographic peak in the *Jatropha podagrica* fruits extract with a retention time of 9.32 min.

Note: The m/z values in purple are the calculated ones. The m/z calculation was based on the relative atomic masses of C (12.0000), H (1.007825), O (15.994915), and N (14.003074)[1]

Identification: As seen in Figure S28, the retention time, MS/MS spectra, and characteristic pears were highly similar. Thus, the chromatographic peaks in the *Jatropha podagrica* fruits extracts were identified as isovitexin (Cas. 38953-85-4).

References:

[1] Jürgen H. Gross. Mass spectrometry. 2013, Beijing: Science press.

Suppl. S2.29 Identification of aromadendrin (Cas. 480-20-6, $C_{15}H_{12}O_6$, M.W. 288.3).

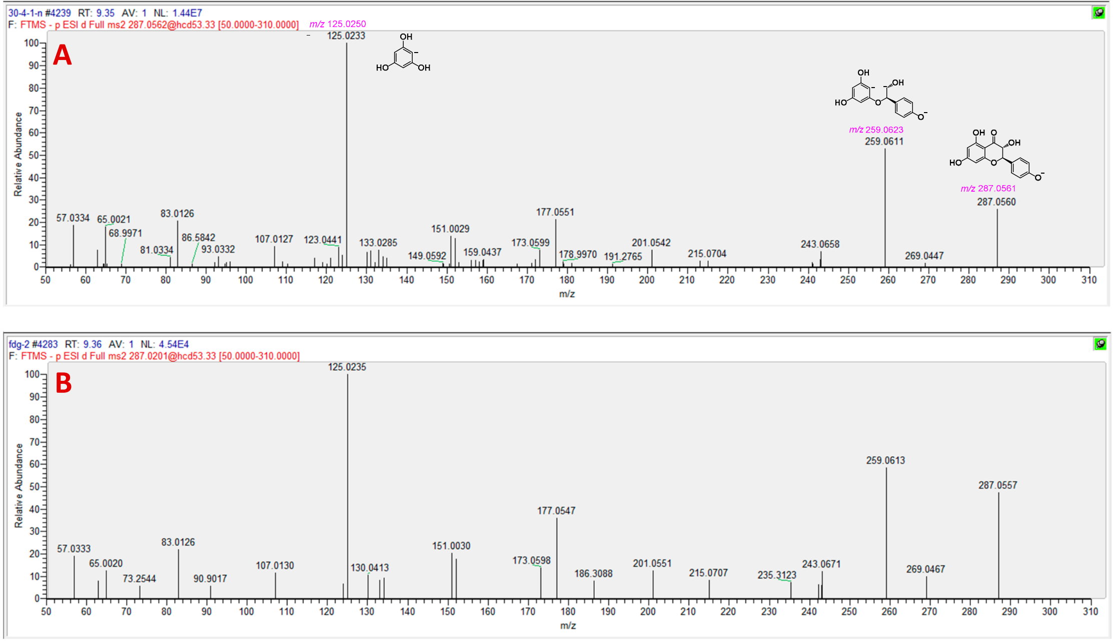

Figure S29. The main results of aromadendrin (Cas. 480-20-6, $C_{15}H_{12}O_6$). (A) The MS/MS fragments of standard aromadendrin with a retention time of 9.35 min. (B) The MS/MS spectra from chromatographic peak in the *Jatropha podagrica* fruits extract with a retention time of 9.36 min.

Note: The m/z values in purple are the calculated ones. The m/z calculation was based on the relative atomic masses of C (12.0000), H (1.007825), O (15.994915), and N (14.003074)[1].

Identification: As seen in Figure S29, the retention time, MS/MS spectra, and characteristic pears were highly similar. Thus, the chromatographic peaks in the *Jatropha podagrica* fruits extracts were identified as aromadendrin (Cas. 480-20-6).

References:

[1] Jürgen H. Gross. Mass spectrometry. 2013, Beijing: Science press.

Suppl. S2.30 Identification of scoparone (Cas. 120-08-1, $C_{11}H_{10}O_4$, M.W. 206.197).

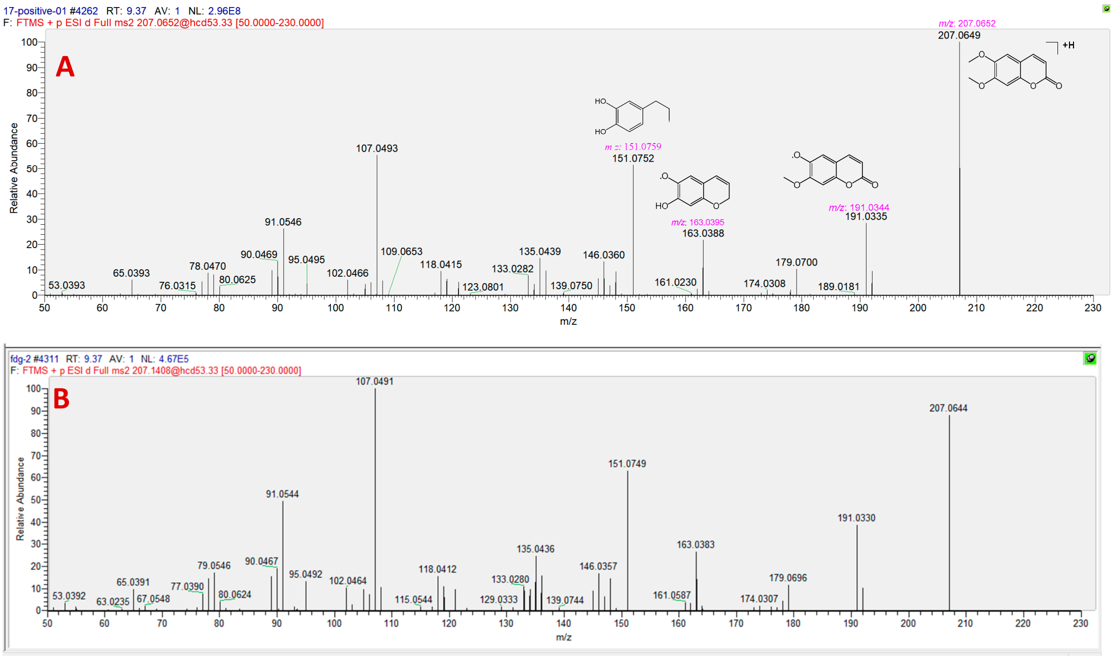

Figure S30. The main results of scoparone (Cas. 120-08-1, $C_{11}H_{10}O_4$). (A) The MS/MS fragments of standard scoparone with a retention time of 9.37 min. (B) The MS/MS spectra from chromatographic peak in the *Jatropha podagrica* fruits extract with a retention time of 9.37min.

Note： The m/z values in purple are the calculated ones. The m/z calculation was based on the relative atomic masses of C (12.0000), H (1.007825), O (15.994915), and N (14.003074)[1]

Identification: As seen in Figure S30, the retention time, MS/MS spectra, and characteristic pears were highly similar. Thus, the chromatographic peaks in the *Jatropha podagrica* fruits extracts were identified as scoparone (Cas. 120-08-1).

Suppl. S2.31 Identification of ellagic acid (Cas. 476-66-4, $C_{14}H_6O_8$, M.W. 302.28).

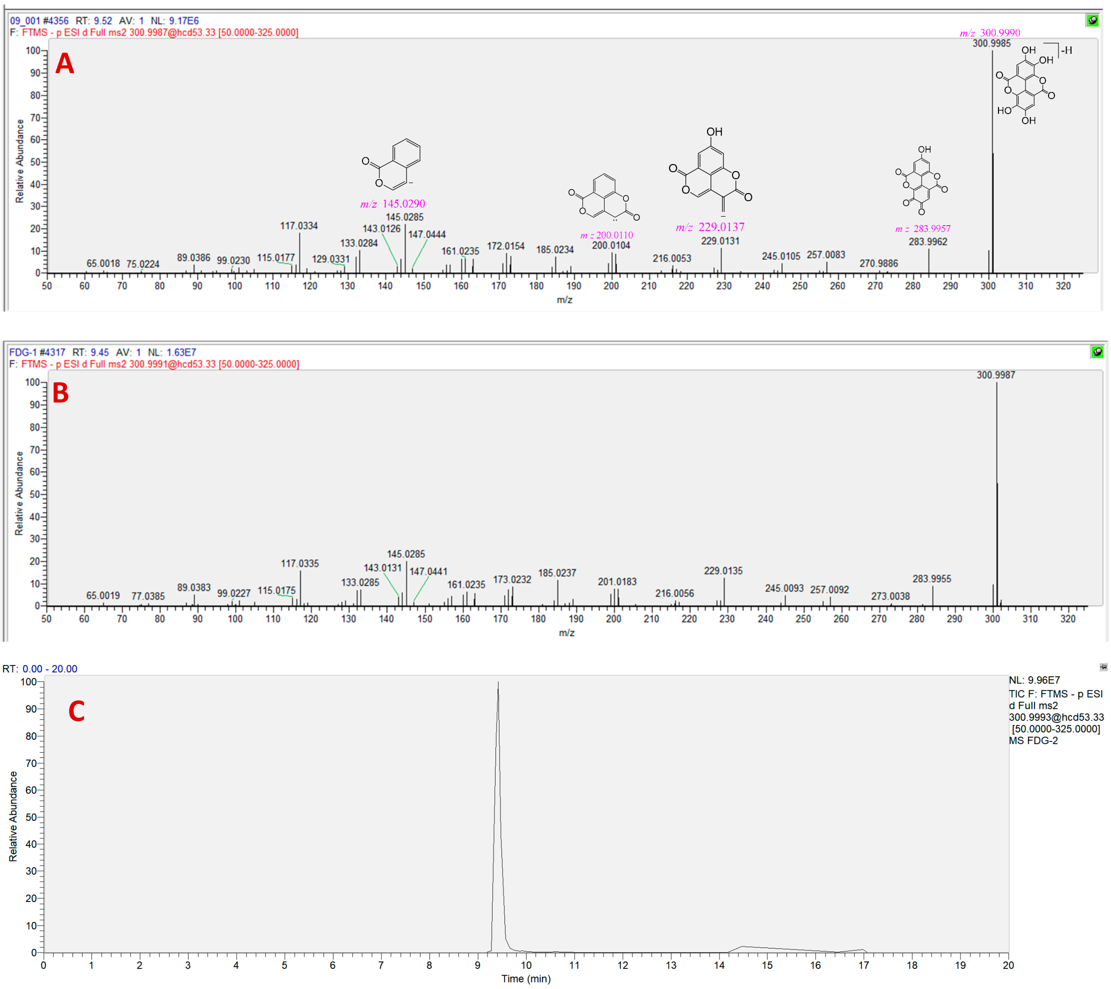

Figure S31. The main results of ellagic acid (Cas. 476-66-4, $C_{14}H_6O_8$) and its corresponding peak in the TIC diagram using UPLC-Q-Orbitrap-MS analysis. (A) The MS/MS fragments of standard ellagic acid with a retention time of 9.52 min. (B) The MS/MS spectra from chromatographic peak in the *Jatropha podagrica* fruits extract with a retention time of 9.45 min. (C) Extracted ion chromatogram of m/z 300.99 from the *Jatropha podagrica* fruits extract.

Note: The m/z values in purple are the calculated ones. The m/z calculation was based on the relative atomic masses of C (12.0000), H (1.007825), O (15.994915), and N (14.003074)[1]

Identification: As seen in Figure S31, the retention time, MS/MS spectra, and characteristic pears were highly similar. Thus, the chromatographic peaks in the *Jatropha podagrica* fruits extracts were identified as ellagic acid (Cas. 476-66-4).

Suppl. S2.32 Identification of naringin (Cas. 10236-47-2, $C_{27}H_{32}O_{14}$, M.W. 580.53).

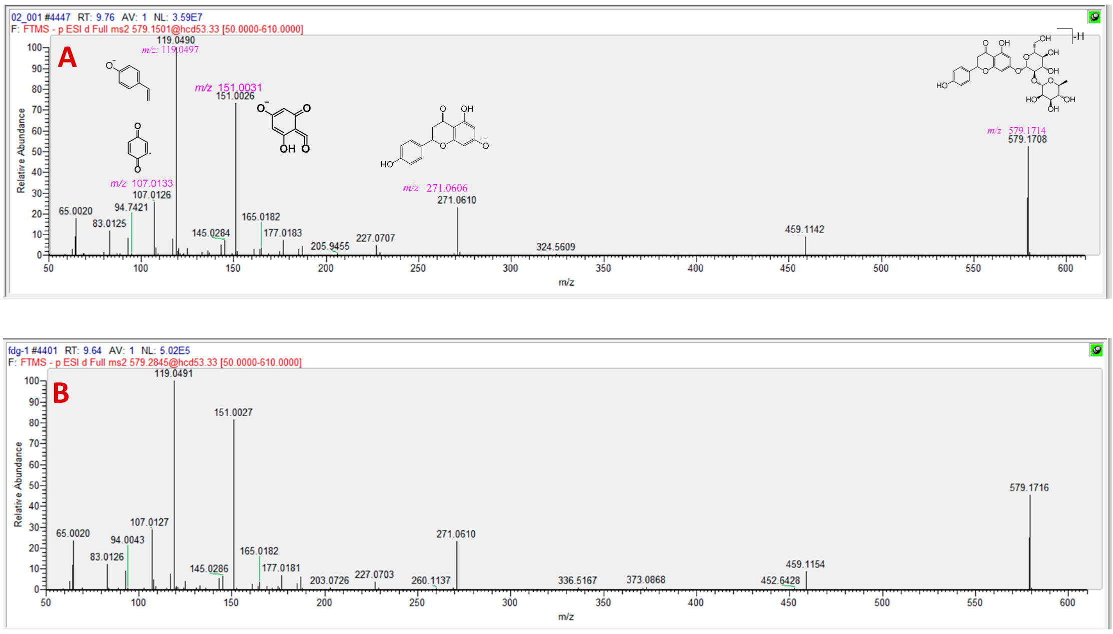

Figure S32. The main results of naringin (Cas. 10236-47-2, $C_{27}H_{32}O_{14}$). (A) The MS/MS fragments of standard naringin with a retention time of 9.76 min. (B) The MS/MS spectra from chromatographic peak in the *Jatropha podagrica* fruits extract with a retention time of 9.64 min.

Note： The m/z values in purple are the calculated ones. The m/z calculation was based on the relative atomic masses of C (12.0000), H (1.007825), O (15.994915), and N (14.003074)[1]

Identification: As seen in Figure S32, the retention time, MS/MS spectra, and characteristic pears were highly similar. Thus, the chromatographic peaks in the *Jatropha podagrica* fruits extracts were identified as naringin (Cas. 10236-47-2).

Suppl. S2.33 Identification of (±)-balanophonin(Cas. 118916-57-7, $C_{20}H_{20}O_6$, M.W. 356.37).

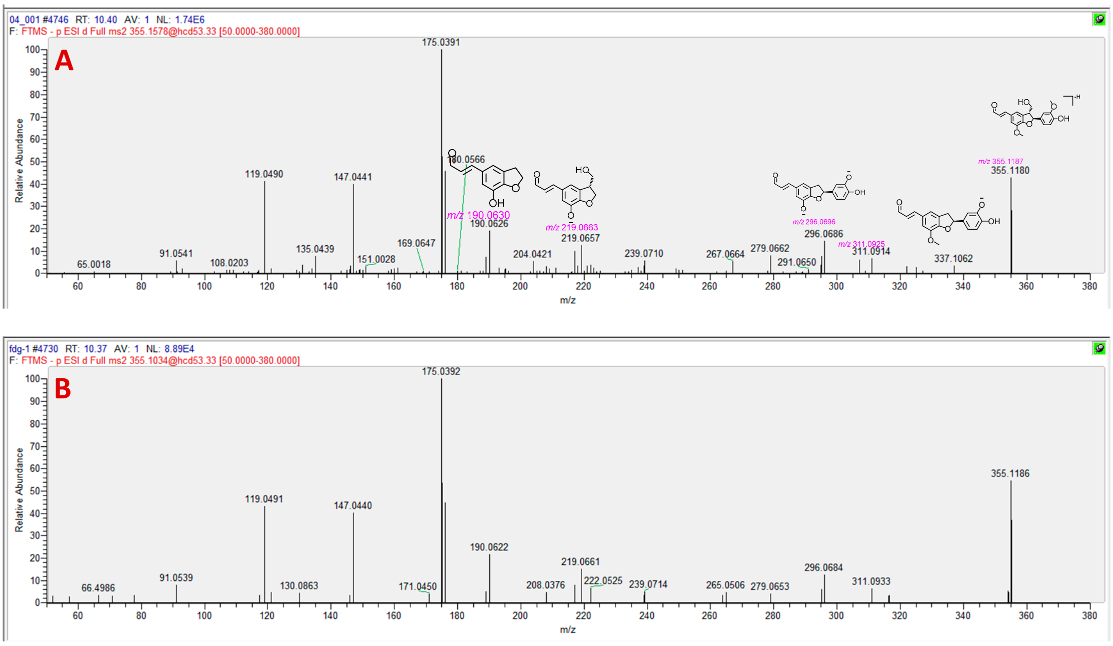

Figure S33. The main results of (±)-balanophonin (Cas. 118916-57-7, $C_{20}H_{20}O_6$). (A) The MS/MS fragments of standard (±)-balanophonin with a retention time of 10.40 min. (B) The MS/MS spectra from chromatographic peak in the *Jatropha podagrica* fruits extract with a retention time of 10.37 min.

Note: The m/z values in purple are the calculated ones. The m/z calculation was based on the relative atomic masses of C (12.0000), H (1.007825), O (15.994915), and N (14.003074)[1]

Identification: As seen in Figure S33, the retention time, MS/MS spectra, and characteristic pears were highly similar. Thus, the chromatographic peaks in the *Jatropha podagrica* fruits extracts were identified as (±)-balanophonin (Cas. 118916-57-7).

Suppl. S2.34 Identification of S-naringenin (Cas. 480-41-1, $C_{15}H_{12}O_5$, M.W. 272.25).

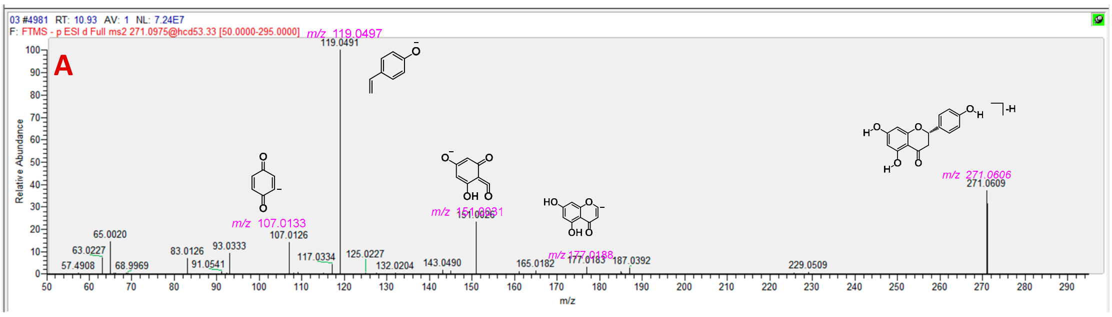

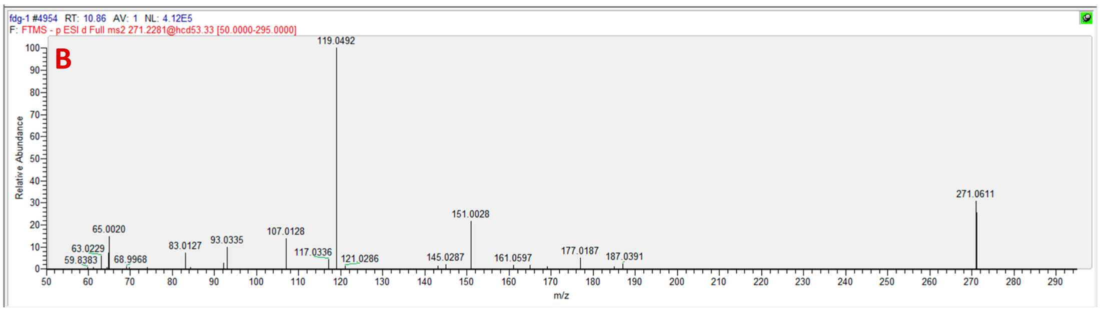

Figure S34. The main results of S-naringenin (Cas. 480-41-1, $C_{15}H_{12}O_5$). (A) The MS/MS fragments of standard S-naringenin with a retention time of 10.93 min. (B) The MS/MS spectra from chromatographic peak in the *Jatropha podagrica* fruits extract with a retention time of 10.86 min.

Note： The m/z values in purple are the calculated ones. The m/z calculation was based on the relative atomic masses of C (12.0000), H (1.007825), O (15.994915), and N (14.003074)[1]

Identification: As seen in Figure S34, the retention time, MS/MS spectra, and characteristic pears were highly similar. Thus, the chromatographic peaks in the *Jatropha podagrica* fruits extracts were identified as S-naringenin (Cas. 480-41-1).

Suppl. S2.35 Identification of luteolin (Cas. 491-70-3, $C_{15}H_{10}O_6$, M.W. 286.24).

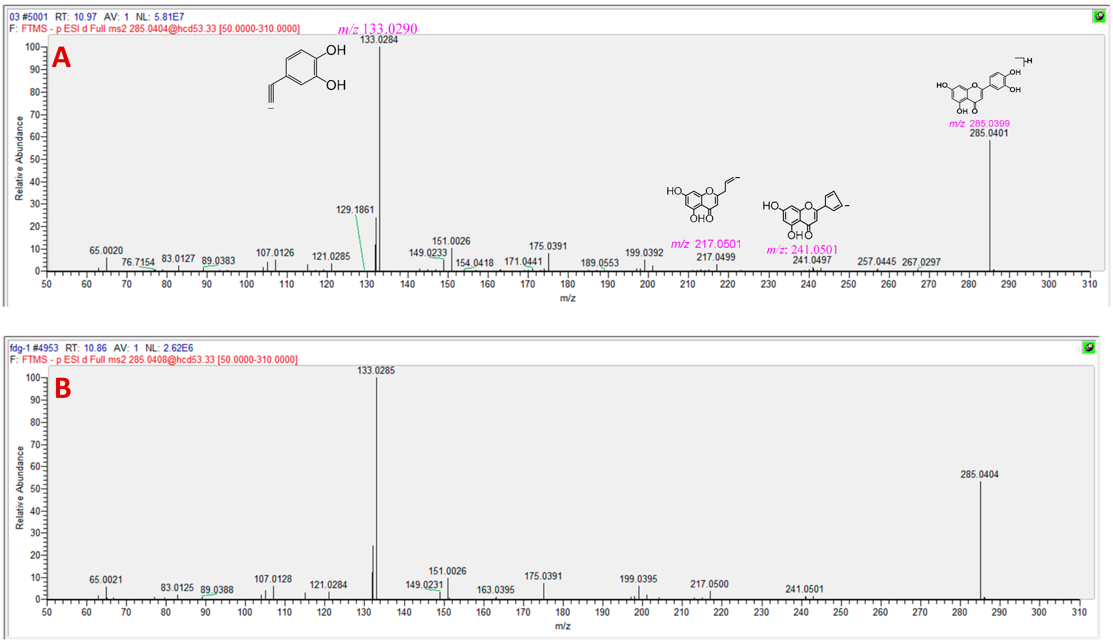

Figure S35. The main results of luteolin (Cas. 491-70-3, $C_{15}H_{10}O_6$). (A) The MS/MS fragments of standard luteolin with a retention time of 10.97 min. (B) The MS/MS spectra from chromatographic peak in the *Jatropha podagrica* fruits extract with a retention time of 10.86 min.

Note: The m/z values in purple are the calculated ones. The m/z calculation was based on the relative atomic masses of C (12.0000), H (1.007825), O (15.994915), and N (14.003074)[1]

Identification: As seen in Figure S35, the retention time, MS/MS spectra, and characteristic pears were highly similar. Thus, the chromatographic peaks in the *Jatropha podagrica* fruits extracts were identified as luteolin (Cas. 491-70-3).

Suppl. S2.36 Identification of kaempferol (Cas. 520-18-3, $C_{15}H_{10}O_6$, M.W. 286.24).

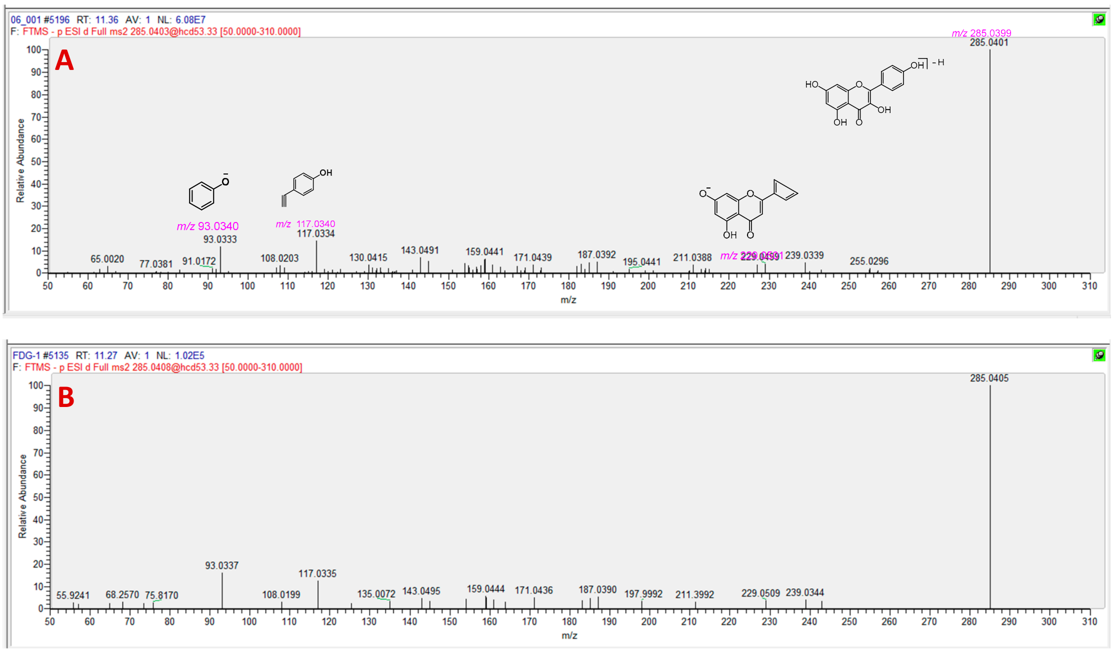

Figure S36. The main results of kaempferol (Cas. 520-18-3, $C_{15}H_{10}O_6$). (A) The MS/MS fragments of standard kaempferol with a retention time of 11.36 min. (B) The MS/MS spectra from chromatographic peak in the *Jatropha podagrica* fruits extract with a retention time of 11.27 min.

Note： The m/z values in purple are the calculated ones. The m/z calculation was based on the relative atomic masses of C (12.0000), H (1.007825), O (15.994915), and N (14.003074)[1]

Identification: As seen in Figure S36, the retention time, MS/MS spectra, and characteristic pears were highly similar. Thus, the chromatographic peaks in the *Jatropha podagrica* fruits extracts were identified as kaempferol (Cas. 520-18-3).

Suppl. S2.37 Identification of apigenin (Cas. 520-36-5, $C_{15}H_{10}O_5$, M.W. 270.24).

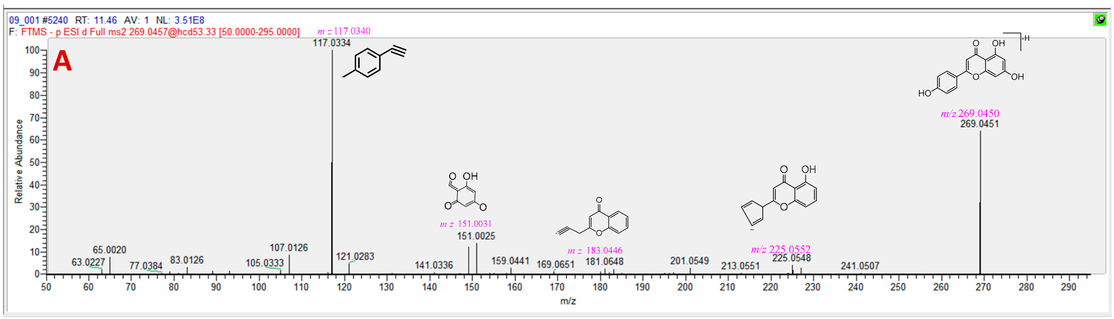

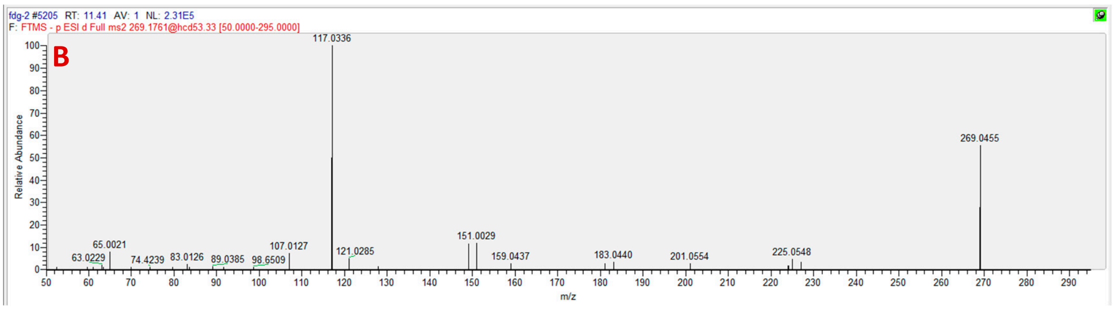

Figure S37. The main results of apigenin (Cas. 520-36-5, $C_{15}H_{10}O_5$). (A) The MS/MS fragments of standard apigenin with a retention time of 11.46 min. (B) The MS/MS spectra from chromatographic peak in the *Jatropha podagrica* fruits extract with a retention time of 11.41 min.

Note: The m/z values in purple are the calculated ones. The m/z calculation was based on the relative atomic masses of C (12.0000), H (1.007825), O (15.994915), and N (14.003074)[1]

Identification: As seen in Figure S37, the retention time, MS/MS spectra, and characteristic pears were highly similar. Thus, the chromatographic peaks in the *Jatropha podagrica* fruits extracts were identified as apigenin (Cas. 520-36-5).

Suppl. S2.38 Identification of 6-gingerol (Cas. 23513-14-6, $C_{17}H_{26}O_3$, M.W. 293.39).

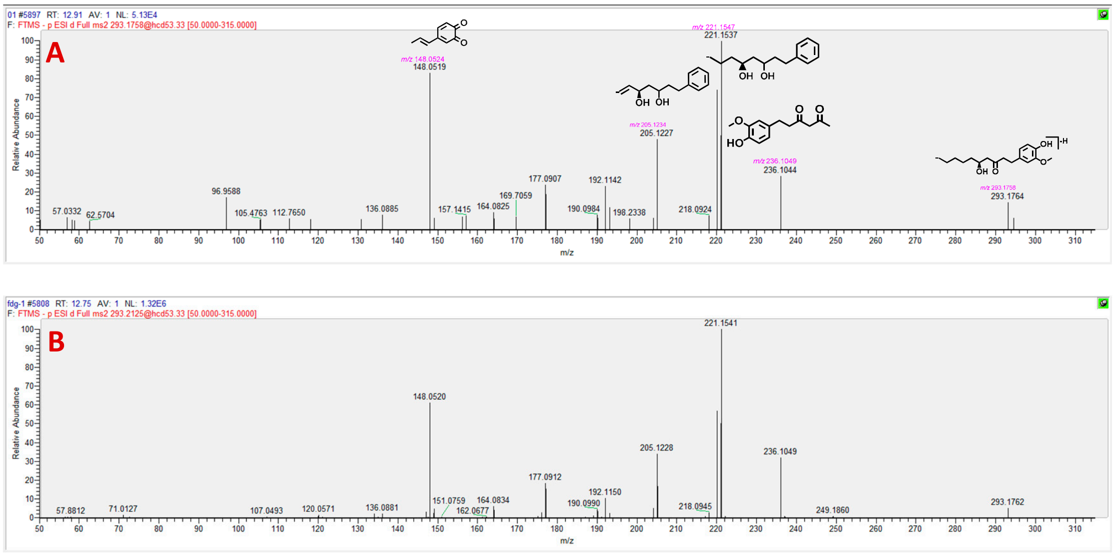

Figure S38. The main results of 6-gingerol (Cas. 23513-14-6, $C_{17}H_{26}O_3$). (A) The MS/MS fragments of standard 6-gingerol with a retention time of 12.91 min. (B) The MS/MS spectra from chromatographic peak in the *Jatropha podagrica* fruits extract with a retention time of 12.75 min.

Note: The m/z values in purple are the calculated ones. The m/z calculation was based on the relative atomic masses of C (12.0000), H (1.007825), O (15.994915), and N (14.003074)[1]

Identification: As seen in Figure S38, the retention time, MS/MS spectra, and characteristic pears were highly similar. Thus, the chromatographic peaks in the *Jatropha podagrica* fruits extracts were identified as 6-gingerol (Cas. 23513-14-6).

Suppl. S2.39 Identification of curcumol (Cas. 4871-97-0, C15H24O2, M.W.236.355).

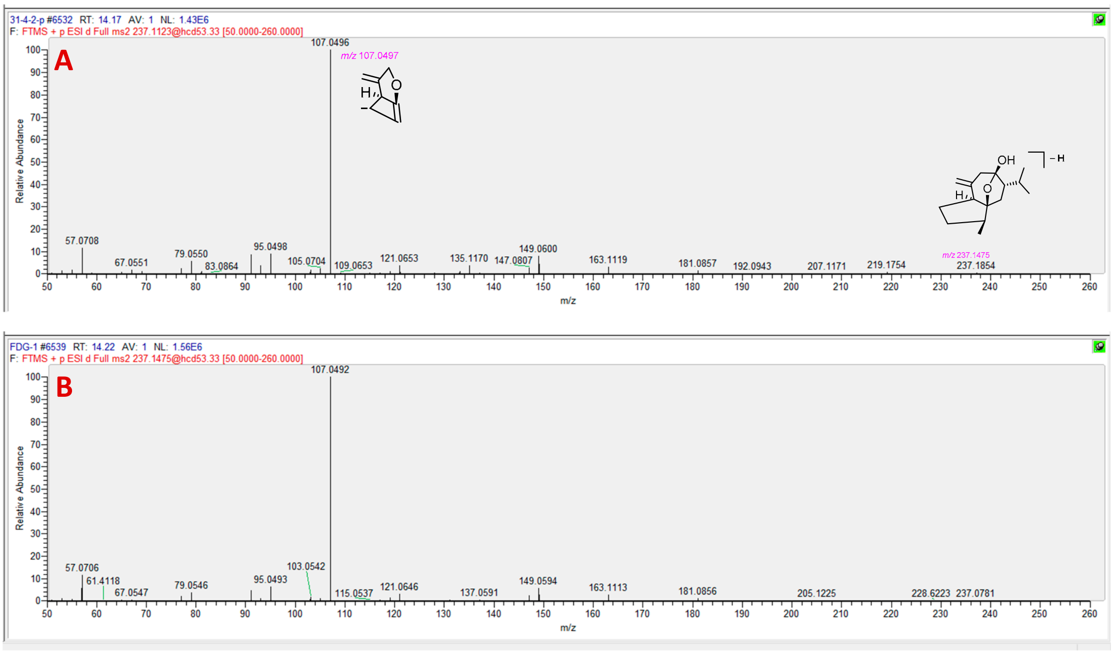

Figure S39. The main results of curcumoln (Cas. 4871-97-0, $C_{15}H_{24}O_2$). (A) The MS/MS fragments of standard curcumol with a retention time of 14.17 min. (B) The MS/MS spectra from chromatographic peak in the *Jatropha podagrica* fruits extract with a retention time of 14.22 min.

Note: The m/z values in purple are the calculated ones. The m/z calculation was based on the relative atomic masses of C (12.0000), H (1.007825), O (15.994915), and N (14.003074)[1]

Identification: As seen in Figure S39, the retention time, MS/MS spectra, and characteristic pears were highly similar. Thus, the chromatographic peaks in the *Jatropha podagrica* fruits extracts were identified as curcumol (Cas. 4871-97-0).

Suppl. S2.40 Identification of α-linolenic acid (Cas. 463-40-1, $C_{18}H_{30}O_2$, M.W. 278.436).

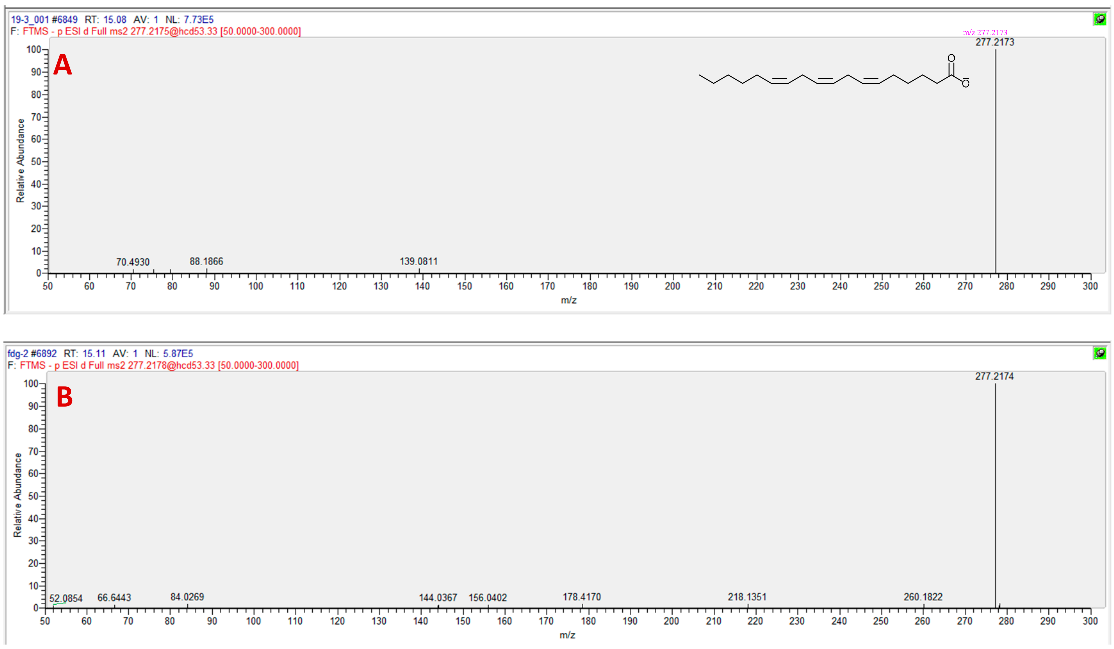

Figure S40. The main results of α-linolenic acid (Cas. 463-40-1, $C_{18}H_{30}O_2$). (A) The MS/MS fragments of standard α-linolenic acid with a retention time of 15.08 min. (B) The MS/MS spectra from chromatographic peak in the *Jatropha podagrica* fruits extract with a retention time of 15.11 min.

Note: The m/z values in purple are the calculated ones. The m/z calculation was based on the relative atomic masses of C (12.0000), H (1.007825), O (15.994915), and N (14.003074)[1]

Identification: As seen in Figure S40, the retention time, MS/MS spectra, and characteristic pears were highly similar. Thus, the chromatographic peaks in the *Jatropha podagrica* fruits extracts were identified as α-linolenic acid (Cas. 463-40-1).

Suppl. S2.41 Identification of linoleic acid (Cas. 60-33-3, $C_{18}H_{32}O_2$, M.W. 280.452).

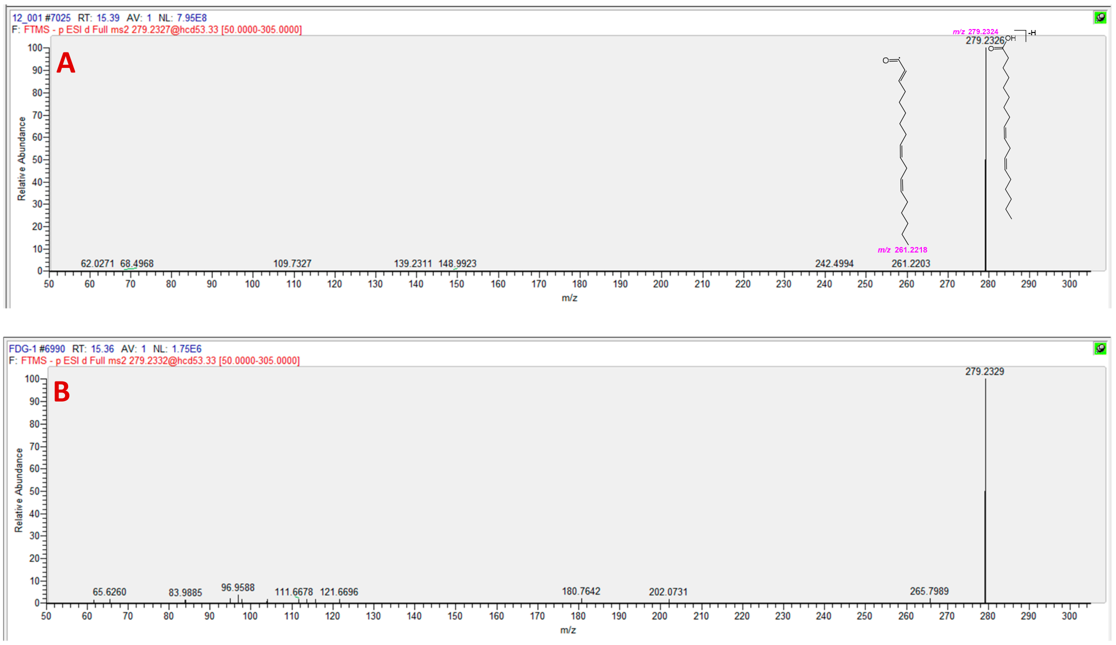

Figure S41. The main results of linoleic acid (Cas. 60-33-3, $C_{18}H_{32}O_2$). (A) The MS/MS fragments of standard linoleic acid with a retention time of 15.39 min. (B) The MS/MS spectra from chromatographic peak in the *Jatropha podagrica* fruits extract with a retention time of 15.36 min.

Note: The m/z values in purple are the calculated ones. The m/z calculation was based on the relative atomic masses of C (12.0000), H (1.007825), O (15.994915), and N (14.003074)[1]

Identification: As seen in Figure S41, the retention time, MS/MS spectra, and characteristic pears were highly similar. Thus, the chromatographic peaks in the *Jatropha podagrica* fruits extracts were identified as linoleic acid (Cas. 60-33-3).

Suppl. S2.42 Identification of phillygenin (Cas. 487-39-8, $C_{21}H_{24}O_6$, M.W. 372.417).

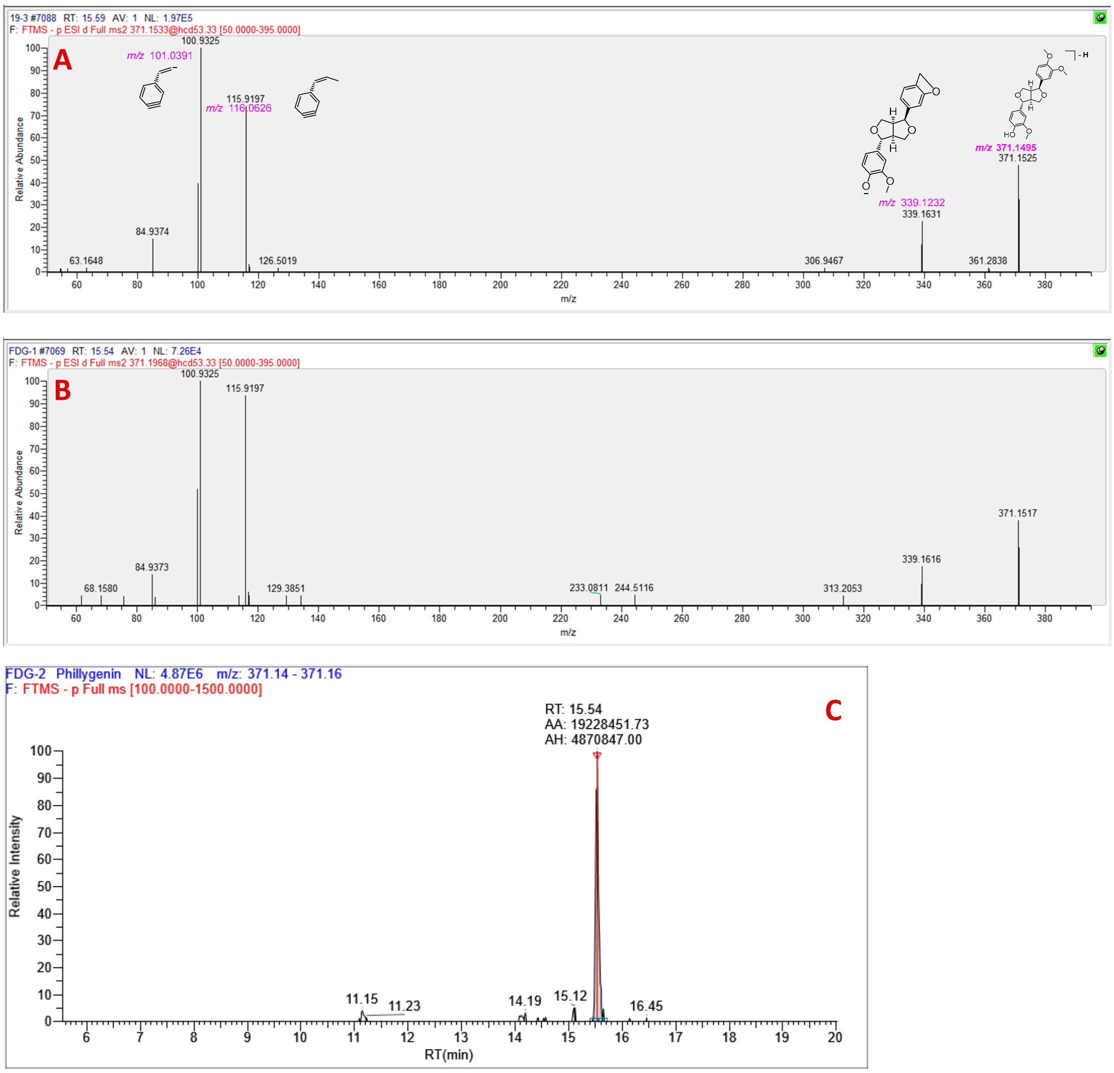

Figure S42. The main results of phillygenin (Cas. 487-39-8, $C_{21}H_{24}O_6$) and its corresponding peak in the TIC diagram using UPLC-Q-Orbitrap-MS analysis. (A) The MS/MS fragments of standard phillygenin with a retention time of 15.59 min. (B) The MS/MS spectra from chromatographic peak in the *Jatropha podagrica* fruits extract with a retention time of 15.54 min. (C) Extracted ion chromatogram of m/z 371.15 from the *Jatropha podagrica* fruits extract.

Note: The m/z values in purple are the calculated ones. The m/z calculation was based on the relative atomic masses of C (12.0000), H (1.007825), O (15.994915), and N (14.003074)[1]

Identification: As seen in Figure S42, the retention time, MS/MS spectra, and characteristic pears were highly similar. Thus, the chromatographic peaks in the *Jatropha podagrica* fruits extracts were identified as phillygenin (Cas. 487-39-8).

Suppl. S2.43 Identification of palmitic acid (Cas. 57-10-3, $C_{16}H_{32}O_2$, M.W. 256.43).

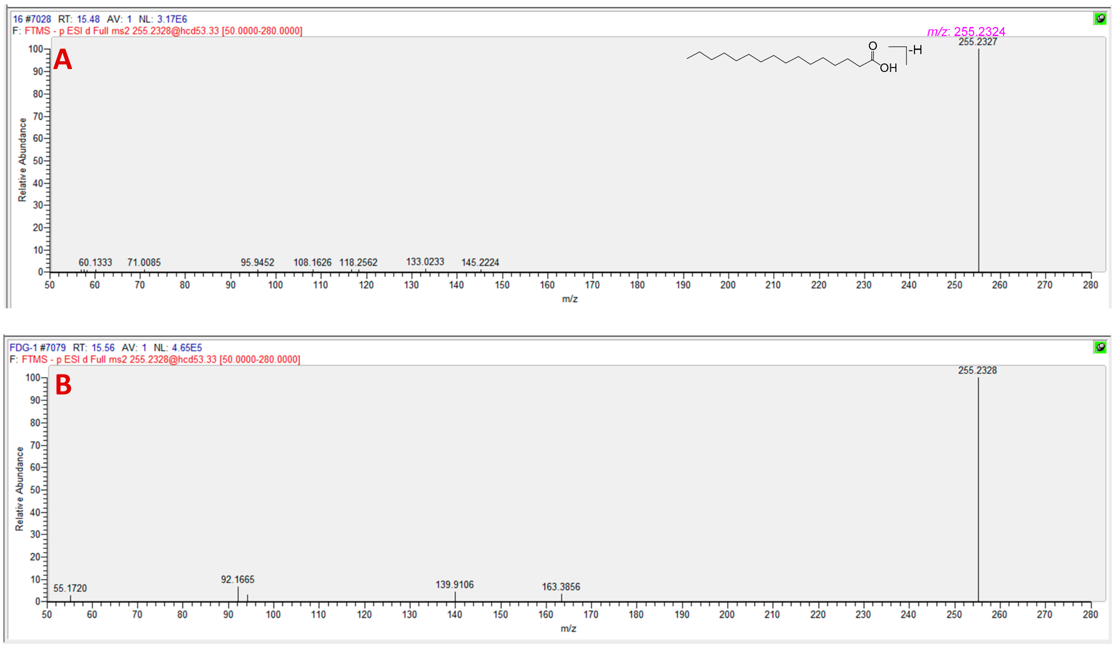

Figure S43. The main results of palmitic acid (Cas. 57-10-3, $C_{16}H_{32}O_2$). (A) The MS/MS fragments of standard palmitic acid with a retention time of 15.48 min. (B) The MS/MS spectra from chromatographic peak in the *Jatropha podagrica* fruits extract with a retention time of 15.56 min.

Note： The m/z values in purple are the calculated ones. The m/z calculation was based on the relative atomic masses of C (12.0000), H (1.007825), O (15.994915), and N (14.003074)[1]

Identification: As seen in Figure S43, the retention time, MS/MS spectra, and characteristic pears were highly similar. Thus, the chromatographic peaks in the *Jatropha podagrica* fruits extracts were identified as palmitic acid (Cas. 57-10-3).

Suppl. S2.44 Identification of oleic acid (Cas. 112-80-1, $C_{18}H_{34}O_2$, M.W. 282.468).

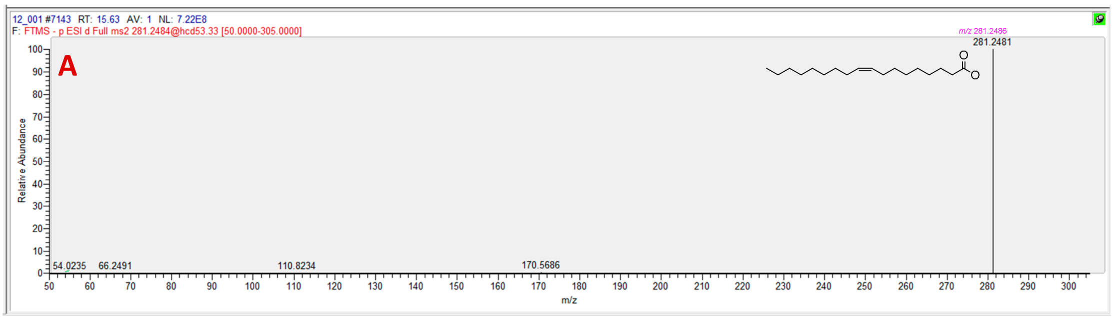

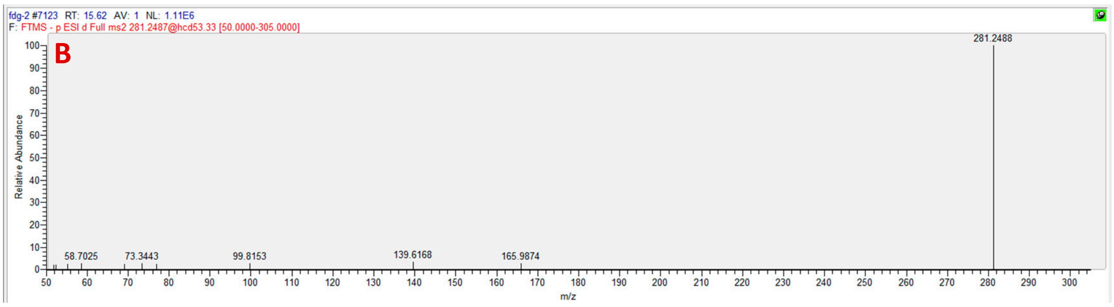

Figure S44. The main results of oleic acid (Cas. 112-80-1, $C_{18}H_{34}O_2$). (A) The MS/MS fragments of standard oleic acid with a retention time of 15.63 min. (B) The MS/MS spectra from chromatographic peak in the *Jatropha podagrica* fruits extract with a retention time of 15.62 min.

Note： The m/z values in purple are the calculated ones. The m/z calculation was based on the relative atomic masses of C (12.0000), H (1.007825), O (15.994915), and N (14.003074)[1]

Identification: As seen in Figure S44, the retention time, MS/MS spectra, and characteristic pears were highly similar. Thus, the chromatographic peaks in the *Jatropha podagrica* fruits extracts were identified as oleic acid (Cas. 112-80-1).

Suppl. S2.45 Identification of ethyl stearate (Cas. 111-61-5, $C_{20}H_{40}O_2$, M.W. 312.5).

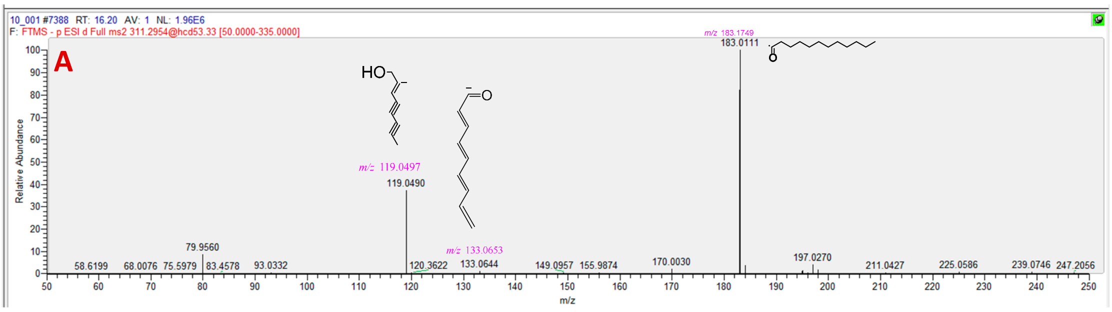

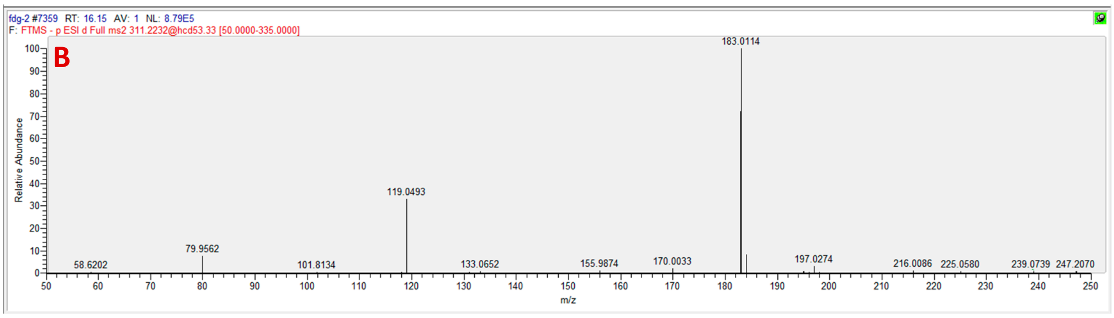

Figure S45. The main results of ethyl stearate (Cas. 111-61-5, $C_{20}H_{40}O_2$). (A) The MS/MS fragments of standard ethyl stearate with a retention time of 16.20 min. (B) The MS/MS spectra from chromatographic peak in the *Jatropha podagrica* fruits extract with a retention time of 16.15 min.

Note: The m/z values in purple are the calculated ones. The m/z calculation was based on the relative atomic masses of C (12.0000), H (1.007825), O (15.994915), and N (14.003074)[1]

Identification: As seen in Figure S45, the retention time, MS/MS spectra, and characteristic pears were highly similar. Thus, the chromatographic peaks in the *Jatropha podagrica* fruits extracts were identified as ethyl stearate (Cas. 111-61-5).

Suppl. S2.46 Identification of (+)-4-cholesten-3-one (Cas. 601-57-0, $C_{27}H_{44}O$, M.W. 384.65).

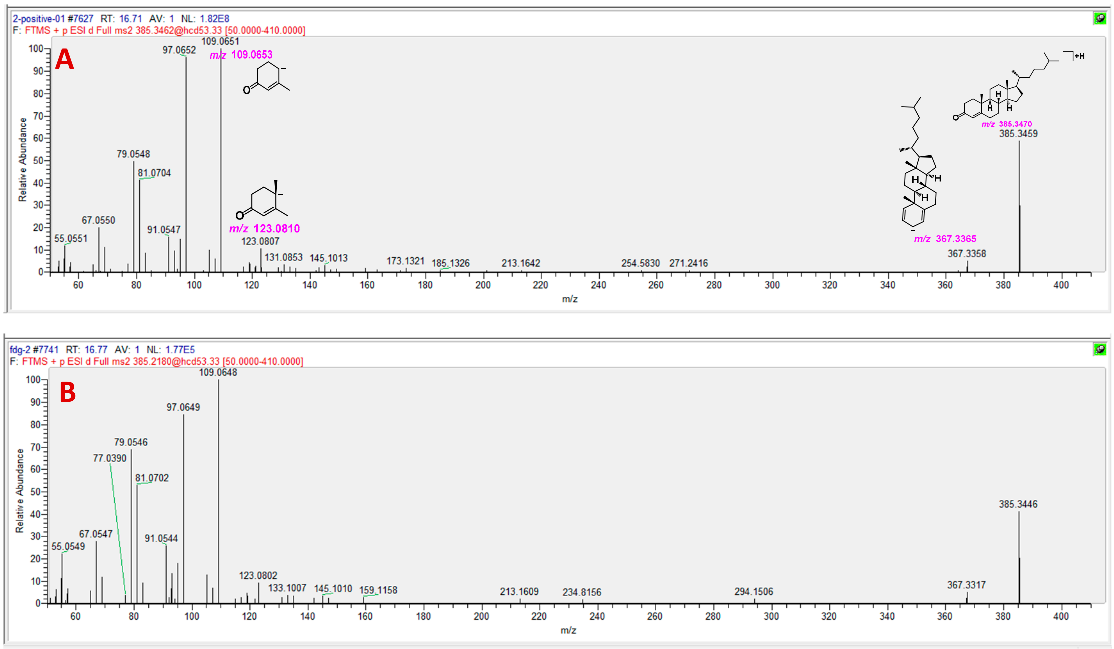

Figure S46. The main results of (+)-4-cholesten-3-one (Cas. 601-57-0, $C_{27}H_{44}O$). (A) The MS/MS fragments of standard (+)-4-cholesten-3-one with a retention time of 16.71 min. (B) The MS/MS spectra from chromatographic peak in the *Jatropha podagrica* fruits extract with a retention time of 16.77 min.

Note: The m/z values in purple are the calculated ones. The m/z calculation was based on the relative atomic masses of C (12.0000), H (1.007825), O (15.994915), and N (14.003074)[1]

Identification: As seen in Figure S46, the retention time, MS/MS spectra, and characteristic pears were highly similar. Thus, the chromatographic peaks in the *Jatropha podagrica* fruits extracts were identified as (+)-4-cholesten-3-one (Cas. 601-57-0).

References:

[1] Jürgen H. Gross. *Mass spectrometry*. 2013, Beijing: Science press.
